# Enzyme-Free Exponential Amplification via Growth and Scission of Crisscross Ribbons from Single-Stranded DNA Components

**DOI:** 10.1021/jacs.3c08205

**Published:** 2023-12-22

**Authors:** Anastasia Ershova, Dionis Minev, F. Eduardo Corea-Dilbert, Devon Yu, Jie Deng, Walter Fontana, William M. Shih

**Affiliations:** † Department of Cancer Biology, Dana-Farber Cancer Institute, Boston, Massachusetts 02215, United States; ‡ Wyss Institute for Biologically Inspired Engineering at Harvard University, Boston, Massachusetts 02115, United States; § Department of Biological Chemistry and Molecular Pharmacology, Harvard Medical School, Boston, Massachusetts 02115, United States; ∥ Department of Systems Biology, Harvard Medical School, Boston, Massachusetts 02115, United States

## Abstract

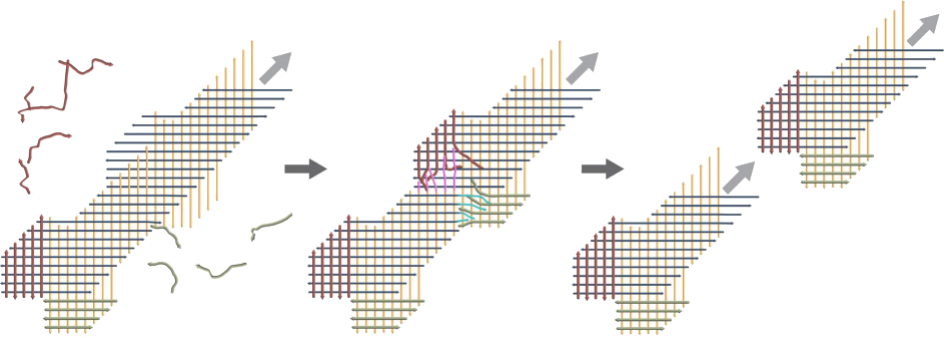

The self-assembly of DNA-based monomers into higher-order structures has significant potential for realizing various biomimetic behaviors including algorithmic assembly, ultrasensitive detection, and self-replication. For these behaviors, it is desirable to implement high energetic barriers to undesired spurious nucleation, where such barriers can be bypassed via seed-initiated assembly. Joint-neighbor capture is a mechanism enabling the construction of such barriers while allowing for algorithmic behaviors, such as bit-copying. Cycles of polymerization with division could accordingly be used for implementing exponential growth in self-replicating materials. Previously, we demonstrated crisscross polymerization, a strategy that attains robust seed-dependent self-assembly of single-stranded DNA and DNA-origami monomers via joint-neighbor capture. Here, we expand the crisscross assembly to achieve autonomous, isothermal exponential amplification of ribbons through their concurrent growth and scission via toehold-mediated strand displacement. We demonstrate how this crisscross chain reaction, or 3CR, can be used as a detection strategy through coupling to single- and double-stranded nucleic acid targets and introduce a rule-based stochastic modeling approach for simulating molecular self-assembly behaviors such as crisscross-ribbon scission.

## Introduction

DNA-based monomers can be programmed to undergo self-assembly into higher-order complexes via DNA hybridization. Examples of such processes include folding of DNA origami^1,2^ and polymerization via tile assembly^3−5^ or hybridization chain reaction (HCR).^6,7^ For exploring behaviors such as algorithmic assembly, ultrasensitive detection, and self-replication, desired growth typically is initiated by a provided seed, while the incidence of spurious nucleation can result in erroneous assemblies or false positives. Particularly for exponential amplification, management of spurious nucleation is of paramount importance, as otherwise, such events can lead to rapid consumption of all resources. Suppression of spurious nucleation is possible via two mechanisms that can be implemented individually or in tandem: kinetic trapping of monomers into inactive states (e.g., HCR) or the presence of a kinetic barrier to nucleation through requiring the stable binding of monomers to be dependent on the engagement with multiple previously captured neighbors (e.g., tile assembly).^8−14^

An interesting feature of joint-neighbor capture is the ability to support informationally rich behaviors such as algorithmic assembly.^3,8,12,13^ One notable algorithmic behavior is bit-copying, which can be used for self-replication. Exponential growth in self-replicating materials can be implemented through combining such growth with scission, for example, through the use of mechanical agitation of crystals formed from DNA tiles^14^ or heating of DNA-origami rafts.^15^ Such architectures can be of interest as model systems for the origins of life, as vehicles for the directed evolution of useful materials, and as strategies for creating adaptive behavior (e.g., B-cell and T-cell diversification and selective amplification for marshaling the body’s limited resources to maximize host defense).

Another use case for the growth and scission of DNA structures is ultrasensitive detection of analytes if the system can be programmed to have the presence of analyte trigger nucleation of growth. Furthermore, the enzyme-free nature of DNA self-assembly can offer potential advantages for detection, including intrinsically lower reagent and storage costs by only using DNA strands and buffers and no direct copying of the analyte as occurs in methods such as PCR. Thus, with a simple heat-denaturation step at the beginning of an amplification protocol, any contaminating amplification (i.e., potential false positives) from previous reaction runs can be destroyed. However, enzyme-free systems to date have exhibited limited performance, thereby motivating the continued investigation of alternate approaches.

Most natural and synthetic systems implementing joint-neighbor capture use monomers that only bind to the nearest neighbors, leading to seed dependence that is only possible under slow, near-reversible growth conditions.^3^ Conversely, crisscross polymerization is an architecture in which “slat” monomers are designed as linear arrangements of binding sites, enabling the engagement of neighbors that are not local in 2D to attain any arbitrary coordination number.^16,17^ For stable binding at the reversible temperature for growth, each slat needs to form a series of weak yet specific bonds with a number of other slats corresponding to the slats’ half-coordination number. Ribbon polymerization propagates by the sequential addition of alternating perpendicular slats as new binding sites are made available by each monomer addition. Such interactions with more than just nearest-neighbor slats allow for extreme levels of cooperativity via highly coordinated joint-neighbor capture. As a consequence, rapid growth can be attained under conditions with exceedingly low levels of spurious nucleation as any spuriously interacting slats do not have sufficient binding energy to initiate stable ribbon formation. Then the addition of a seed that preorganizes an initial set of high-coordination binding sites allows the system to bypass this large entropic barrier and thereby facilitate rapid ribbon assembly.

By coupling linear crisscross growth with toehold-mediated strand displacement (TMSD),^18,19^ we introduce an expanded strategy that allows for autonomous enzyme-free isothermal exponential amplification. This is achieved through concurrent growth and scission of crisscross ribbons, a process we have named crisscross chain reaction (3CR). Compared to linear growth alone, 3CR has the potential to attain higher sensitivity through generating greater amplification from a single seed. As a proof of concept, we couple 3CR amplification to the detection of nucleic acid biomarkers with a limit of detection of <100 fM after overnight assembly and highlight possible future directions to improve detection speed and sensitivity. We further show how graph-rewrite rule-based stochastic simulations^20,21^ can be used to study self-assembling systems like 3CR at a level of abstraction above specific monomer and sequence design while still allowing for the specification of programmable interactions at the level of individual binding sites.

## Results

### Exponential Amplification via Ribbon Growth and Scission

**Figure 1:**
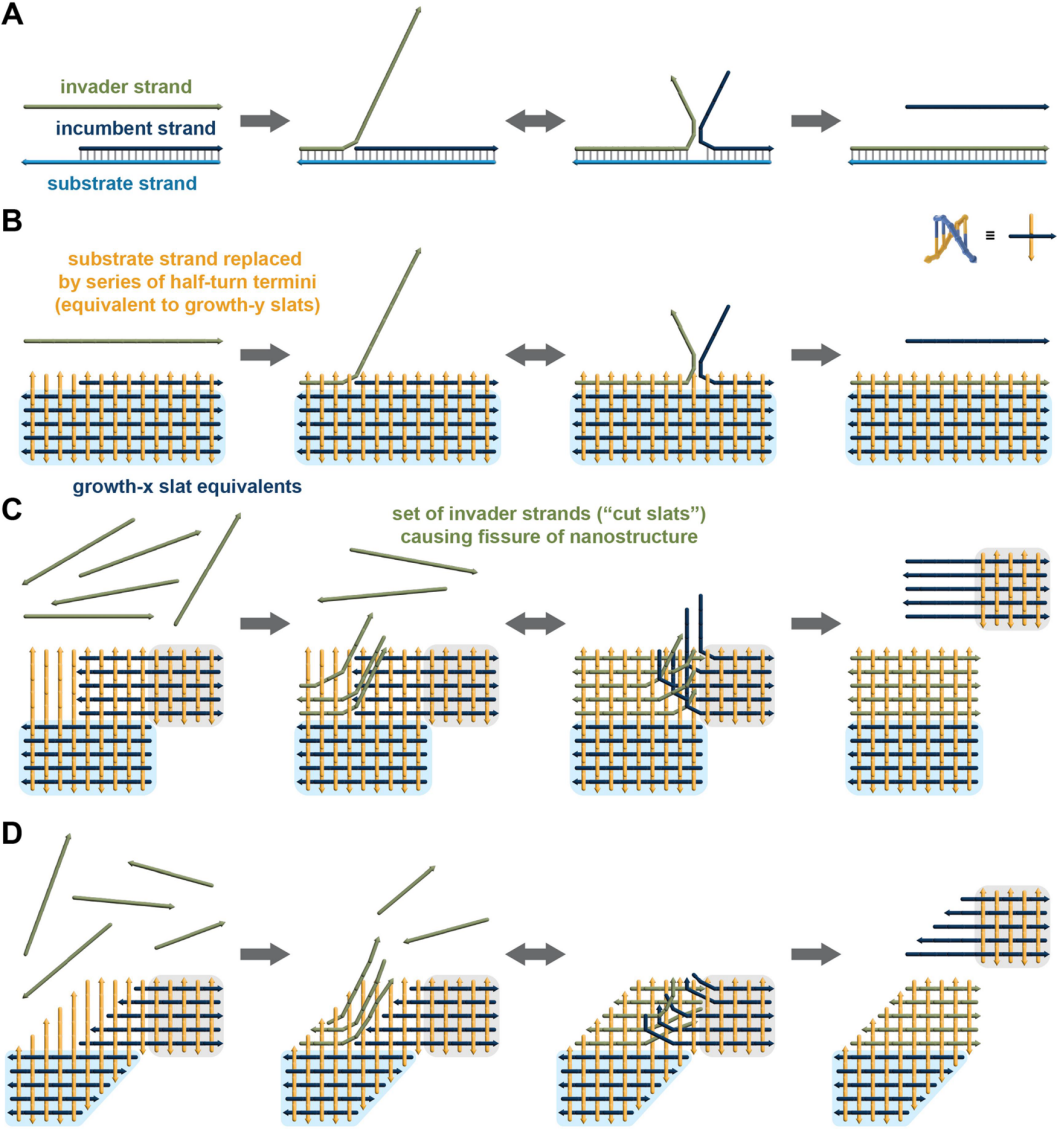
Scission of a finite crisscross DNA structure through toehold-mediated displacement by a set of invader (i.e., cut) strands. (A) Principle of classical toehold-mediated strand displacement. An invader strand (green) engages a toehold domain on a substrate strand (light blue) and then proceeds to liberate a bound incumbent strand (dark blue) through branch migration. (B) Strand displacement where the substrate strand, including its toehold domain, is functionally replaced by a series of half-turn (5 or 6 bp) strand termini arranged on the face of a crisscross structure. (C) Fissure of a crisscross nanostructure through toehold-mediated recruitment of a set of invader strands followed by joint branch migration. (D) Analogous fissure of a crisscross ribbon fragment exhibiting xy growth (i.e., alternating staggered x and y slats). Light blue and gray boxes in B–D outline regions not involved in the strand displacement. See Figure 2 for how such a scission can be coupled with growth.

**Figure 2:**
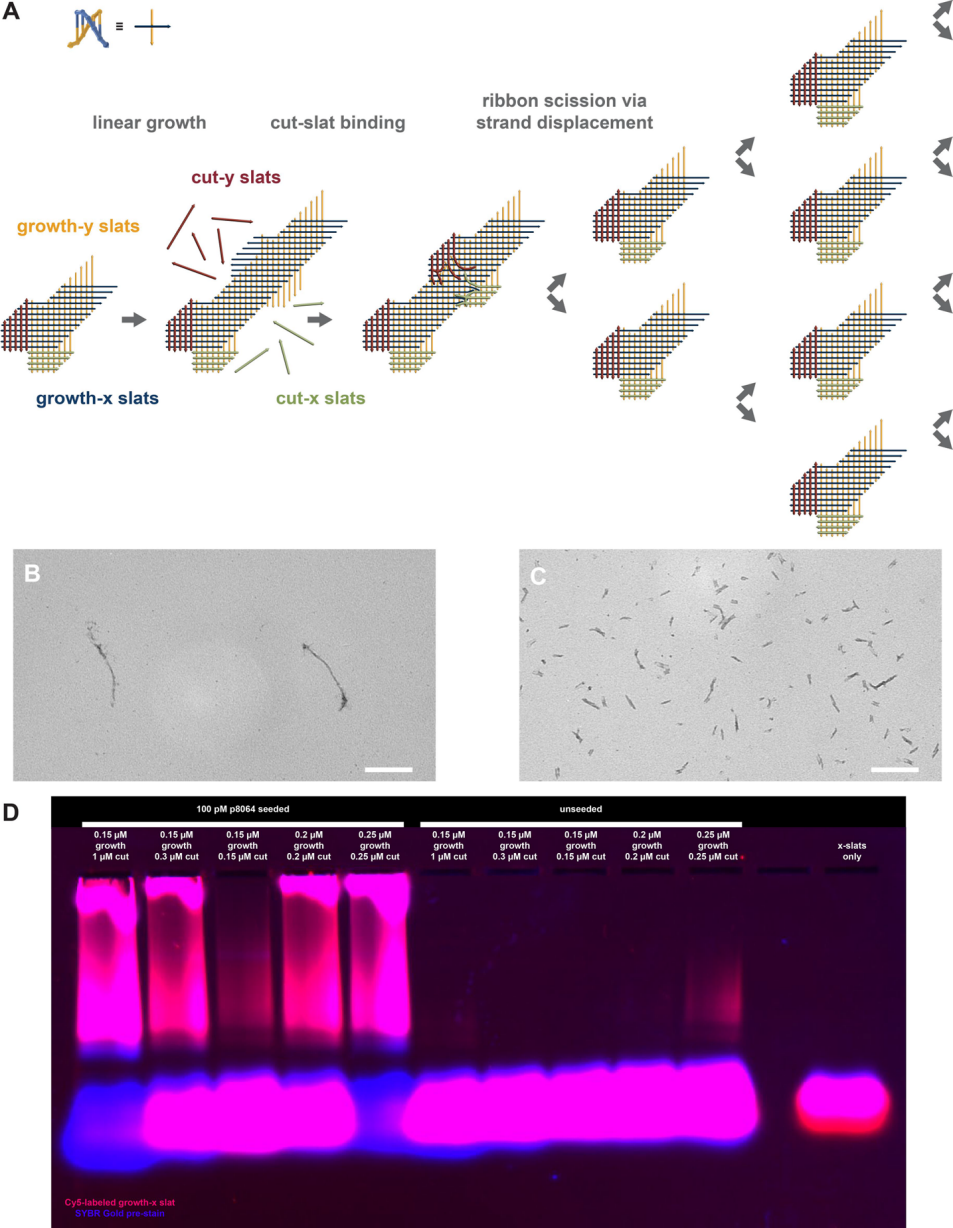
Principle of 3CR for exponential amplification of xy ribbons via isothermal growth and scission. (A) Schematic of the design for a v5 ribbon (detailed scadnano design in Supp. Figure 1C). Each intersection between a horizontal and vertical line represents a half-turn (5–6 base pairs) of dsDNA. Through linear growth, growth slats with single-stranded extensions are added to the ribbon in a specific order that allows cut slats to bind. Once bound, the cut slats compete with the growth slats via toehold-mediated strand displacement (see Supp. Movie 1 for a more in-depth view of ribbon scission via strand displacement). Once the cut slats had displaced the growth slats, the ribbon is severed into two fragments. Each of these fragments is capable of further growth and scission. (B) TEM image after ribbon growth without scission (mean length 409 nm, standard deviation 176 nm, based on 22 measurements). (C) TEM image after combined ribbon growth and scission (mean length of 44 nm, standard deviation of 19 nm, based on 226 measurements, corresponding to predominantly fully cut ribbons). Production of ribbons, long or short, is seed-dependent (see Figure 3). Note that linear ribbons appear twisted and irregular in width due to the use of 11 bp/turn and the presence of single-stranded extensions, which, when unbound, tend to cause aggregation. Scale bars: 200 nm. See “Assembly Reactions” for details of the conditions used. (D) Comparison of seeded and unseeded 3CR amplification at different slat concentrations with cy5-fluorophore 3′ labeling of the top x-slat from the repeat unit in A and Supp. Figure 1C (present at roughly 50% of the concentration of other growth slats). The red fluorescent signal (i.e., gel image captured with a red filter) is from this labeled x-slat, while the blue fluorescent signal (i.e., gel image captured with a blue filter) is from SYBR-Gold prestaining of the agarose gel. The fast-migrating bright species at the bottom of all agarose gels are unincorporated slats. See “Assembly Reactions” for details of conditions used.

Taking linear ribbons made of ssDNA slats^16^ as a starting point, we appended additional binding sites to each *x*- and *y*- “growth” slat that, upon ribbon assembly, form a parallel array of single-stranded extensions on the “west” and “south” sides of the ribbon, respectively. Just as a parallel array of growth slats of one orientation forms multistranded binding sites for growth slats of the perpendicular orientation, the parallel extensions can collectively form multistranded “toeholds” for joint capture of the initial set of domains of perpendicular “cut” slats. Cut slats captured in this way are then positioned for the competitive displacement of several segments of an existing growth slat. Thus, cut slats can invade the ribbon via TMSD (Figure 1) and are collectively able to sever the ribbon in two. This results in an additional growth front becoming available (Figure 2A, Supp. Figure 1 and Supp. Movie 1). Since both the growth slats and cut slats are present in a one-pot reaction, the 3CR system is, in principle, designed such that the growth/scission cycle repeats autonomously, resulting in an exponential amplification process that generates many short ribbon fragments following exposure to a single seed. Assuming 100% efficient scission, the 3CR system should result in a doubling of amplification (i.e., the mass of ribbons generated) following each instance of linear growth of a ribbon repeat unit. Given that the process of scission via TMSD follows the form of a random walk, increasing the relative binding strength of cut slats as compared to growth slats can introduce kinetic ratchets biasing the random walk, effectively shortening the time that it takes to resolve. For example, the introduction of unpaired nucleotides or guanine-thymine (G-T) wobble base pairs in growth slats can favor the binding of cut slats that form stronger guanine-cytosine (G-C) base pairs.

In order to achieve 3CR amplification with appreciable rates of growth and scission, we followed an iterative design pipeline of experimentally optimizing the slat sequences. We found that adding long extensions and wobbles on a set of 24 slats in a single design step would result in significant decreases in growth rate, even for a dozen or so variant sequence sets (data not shown). We hypothesized that long extensions may exhibit a sequence-dependent facility to interact with each other leading to kinetic traps that slow growth; furthermore, the introduction of wobbles could give rise to slow growth in a sequence-dependent fashion. To combat this, we decided on an iterative buildup of wobbles and slat extensions through a multistep design process.

First, we screened eight “v6” (i.e., monomer half-coordination number *n* = 6 and 12 binding sites in the core growth-slat region, as per the definitions in Minev et al.^16^) sequence variants each with 8 wobbles in the following context: “A/T GNG C/G A/T” in 6-nt binding sites, where N represents all four possible bases across from a G on the opposing strand (Supp. Figure 2). For the sequence variant with the fastest relative growth from this screen, we designed three possible 6-segment extensions for every one of the 24 growth slats (Supp. Figure 1A). We then screened all possible combinations of three extensions at a time to build up a set of 24 extension sequences that still maintained relatively fast growth kinetics (Supp. Figures 3–10). From here, we derived a v5 design (i.e., monomer half-coordination number *n* = 5 and 10 binding sites in the core growth-slat region) with 6-segment extensions by removing the two middle binding sites from every growth-slat (Supp. Figure 1B). While, in principle, v6 assembly could provide greater robustness to spurious nucleation by allowing growth at a higher temperature, we used the v5 design as our default for this study, reasoning that (a) faster scission kinetics are expected as fewer binding sites need to be displaced for a thinner ribbon (Supp. Figure 11) and (b) the shorter slat length enabled us to order strands from IDT at lower-cost synthesis scales. Using this set of growth slats, we screened cut slats that bind between 1 and 6 segments as toeholds; we found that 4-segment binding produced the strongest overall amplification (Supp. Figure 12). We then truncated the extensions to remove any binding sites that were not necessary for 4-segment cutting (Supp. Figure 1C–E). It is worth noting that in this screening procedure, we did not consider the rate of spurious nucleation, which we have also found to have a strong sequence dependence (Supp. Figure 13). Hence, a future direction to improve the limit of detection of the 3CR system would be to carry out more thorough sequence optimization considering both the rate of amplification and spurious nucleation simultaneously.

To aid the nucleation of cut-slat binding to the single-stranded extensions, we introduced “filler” slats that provide a stacking interface for cut-y-slat binding, thereby assisting the formation of the dense half-turn crossover pattern by the cut slats (Supp. Figure 1A,B and 14–16). The requirement for filler slats can be mitigated by lengthening the growth slats such that these slats themselves provide a stacking interface for the cut slats (Supp. Figures 1C and 11). In this case, lower rates of spurious nucleation may be expected as none of the slats engages more than one binding site on any given other slat (Supp. Figures 17–21). We note that implementations of 3CR with more rigid monomers such as DNA-origami slats^17^ may not require filler slats.

**Figure 3:**
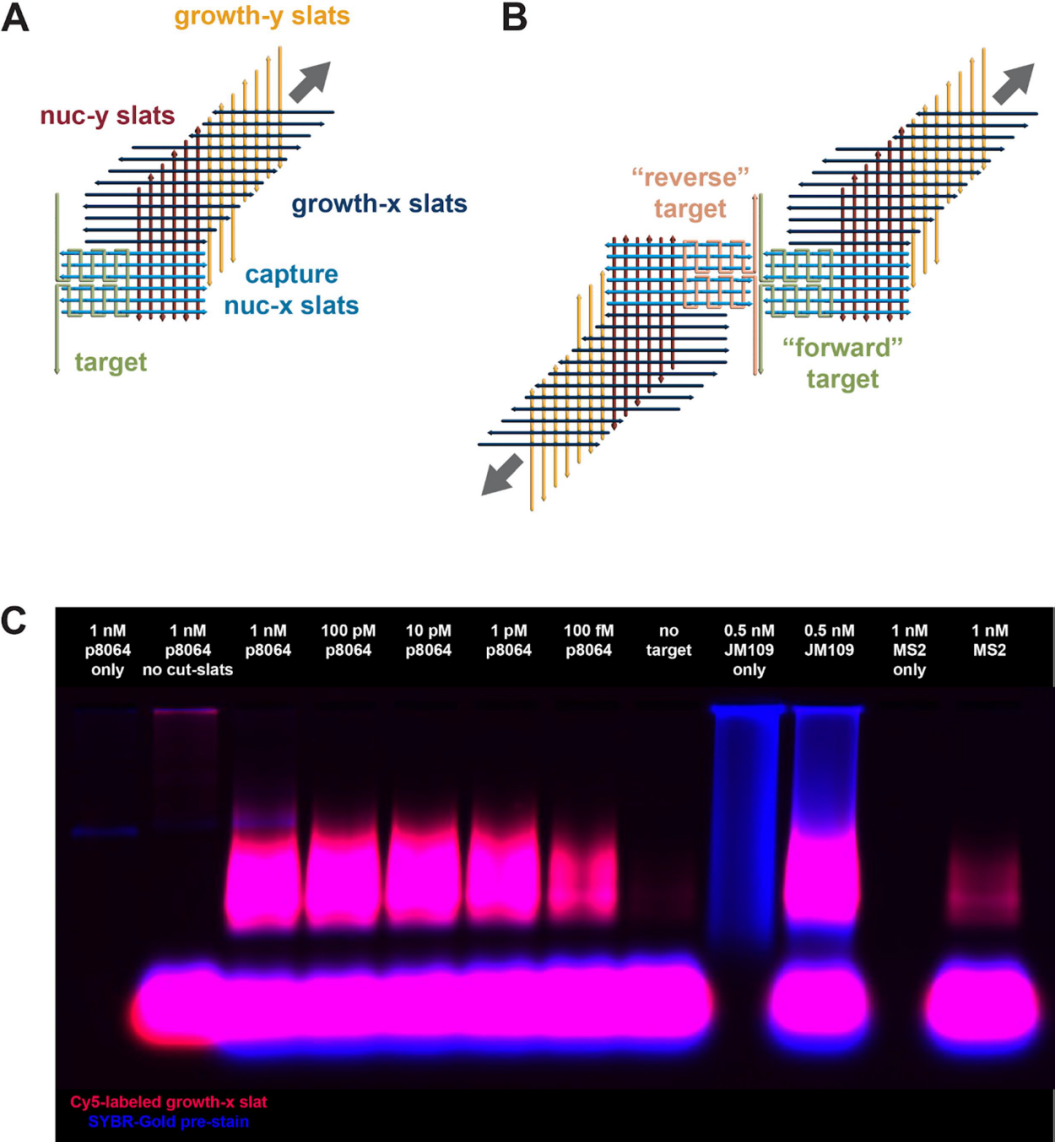
Target-dependent nanoseed formation leading to the 3CR exponential growth of v5 ribbons. (A) Design of nanoseed formation from ssDNA or ssRNA, with coupling to v5 crisscross growth and scission (cut slats omitted from the cartoon for clarity). As in Figure 2A, one intersection between a horizontal and vertical line represents a half-turn (5–6 base pairs) of DNA. (B) Design of twinned-nanoseed formation from dsDNA. It is likely that nanoseed formation only proceeds efficiently for targets that are kinetically trapped in single-stranded states, e.g., through denaturation followed by incomplete renaturation, and that thereby are available for sequestration by the capture slats. (C) 3CR detection of different targets using a v5 design (p8064 ssDNA, JM109 *E. coli* dsDNA, MS2 RNA). The red fluorescent signal (i.e., gel image captured with a red filter) is from the top x-slat in Supp. Figure 1C labeled with a Cy5-fluorophore on its 3′ end, while the blue fluorescent signal (i.e., gel image captured with a blue filter) is from SYBR-Gold prestaining of the agarose gel. The fast-migrating bright species at the bottom of all agarose gels are the unincorporated slats. See “Assembly Reactions” for details of conditions used.

Based on this optimization, we settled on the following default design for this work: “v5” ribbons with 4-segment extensions (Figure 2 and Supp. Figure 1C), 6 G-T wobble base pairs per repeat, gel-purified cut slats (Supp. Figure 22), and detection using an 11 bp/turn “sparse” crossover nanoseed (Figure 3, Supp. Figure 23, and discussion in Section “Detection of nucleic acid targets”, based on the design first introduced in Supp. Figure 40 from Minev et al.^16^) capturing the M13 bacteriophage-derived “p8064” ssDNA scaffold.

We were thus able to implement a system with a doubling rate of approximately every ∼2 h, providing significantly greater amplification than linear assembly alone (Supp. Figure 24). In some assembly conditions, the rate of ribbon assembly was sufficient to achieve depletion of growth slats, albeit with detectable levels of spurious nucleation (Figure 2D). By pregrowing ribbons in the absence of cut slats (Supp. Figure 25) and then diluting the ribbons 20-fold into reactions with variable concentrations of cut slats, we found that significant scission is possible in as little as 5 min, suggesting that growth is rate-limiting for this v5 3CR implementation (Supp. Figure 26). In contrast, for the parent v6 design, significantly slower scission rates were observed (Supp. Figure 27). Interestingly, for the v5 design, cut-y slats alone were sufficient for scission (at approximately half the rate of cut-y and cut-x slats together), while cut-x slats alone did not generate any detectable scission (Supp. Figures 26 and 28). This might be due to the asymmetry of this design with significantly more scission occurring in the *y*-direction, especially as this phenomenon was not observed in an alternative v6 3CR design (Supp. Figure 29).

### Detection of Nucleic Acid Targets

To demonstrate the ability of 3CR to detect nucleic acid biomarkers, we designed “capture” slats that bind to a target sequence to form a “nanoseed” (Figure 3A, B and Supp. Figure 30). This nanoseed can subsequently trigger exponential crisscross polymerization, as described above. For detecting double-stranded targets, we designed capture slats for both the forward and reverse target strands, with a 5-nucleotide stagger in between to reduce hybridization between the two sets of capture strands. Simultaneous capture of both target strands away from each other almost doubles the number of base pairs and therefore thermodynamically favors nanoseed formation over reannealing of the dsDNA target^22^ (Figure 3B).

By designing capture slats against different sequences, we were able to detect viral ssDNA (M13 bacteriophage) (Figures 2B, C and 3C), viral RNA (MS2, Figure 3C and Supp. Figure 31), sonicated bacterial genomic dsDNA (JM109 *E. coli*, Figure 3C and Supp. Figure 32), and viral dsDNA (lambda, Supp. Figure 33). Under the experimental conditions used in Figure 3C, the efficiency of JM109 dsDNA detection relative to that of p8064 ssDNA was ∼1% and of MS2 ssRNA was ∼0.01% (using UV-absorbance as quantification of starting target concentrations and ignoring potential sample degradation). In the case of JM109, the lower efficiency of detection could be due to the additional complexity of nanoseed formation from longer dsDNA and the potential interference of the sample with crisscross ribbon assembly. Further optimization of experimental conditions (e.g., via temperature ramps) and design changes could help boost dsDNA detection efficiency. For MS2 RNA, the low efficiency could be a byproduct of RNA degradation and inefficient capture, especially given that the nanoseed design and experimental conditions were set to work for DNA detection. Capture efficiency could potentially be improved through the design of adapter regions between RNA–DNA duplexes and the higher crossover density crisscross regions and experimental optimization at alternative buffer conditions favoring RNA stability. We note that with linear growth with a different nanoseed design capturing a longer target (408 nt c.f. 188 nt) and under different buffer conditions, we were able to detect MS2 ssRNA with ∼40% efficiency relative to that of p8064 ssDNA, including simultaneous detection of MS2 with p8064 to produce an additive signal (Supp. Figure 34), implying that there is potential for improvement of RNA detection with 3CR with further optimization. Furthermore, we note that due to the presence of detectable albeit low levels of spurious nucleation (“no target” lane in Figure 3C), any future detection applications should be calibrated against a no-target control to ensure detection above background system noise.

To simplify the gel-based analysis of the effect of design parameters on nanoseed formation and not growth, we used the linear v6.1 growth-slat sequences investigated previously,^16^ instead of exponential growth and scission. With the detection of linear lambda dsDNA in this scheme, we attained more robust detection of target sequences near the termini versus the middle of the genome (Supp. Figure 33). Interestingly, we also found that detection is possible even without denaturing the dsDNA target and by capturing only either the forward or reverse strand. We hypothesized that some fraction of the nominally dsDNA target actually is in a kinetically trapped ssDNA state, enabling capture-slat invasion without any prior denaturation. To explore further this hypothesis, we designed a synthetic sequence (Supp. Figure 35) and generated ssDNA, circular dsDNA plasmid, and linearized dsDNA plasmid versions. As expected, the detection efficiency of the dsDNA targets was nonzero but significantly lower than the ssDNA version. In order to induce kinetically trapped ssDNA states in the dsDNA target, we heat-denatured the linearized target at 85 °C and then rapidly quenched the reaction in ice, finding that this treatment rescues amplification.

By altering the density of crossovers in the nanoseed (Supp. Figure 30) and experimental conditions, we were able to adjust the tolerance of nanoseed formation to the presence of mismatches in the target sequence (Supp. Figure 36). Such tunable specificity could potentially be useful for the detection of rapidly evolving pathogens, overcoming a limitation of PCR where mutations in primer or probe binding sites result in significant decreases in sensitivity.^23,24^ For example, below the reversible temperature, we found that nanoseeds with “sparse” 1.5-turn crossovers can recognize a sequence with as low as ∼90% identity if the mismatches are evenly spread out across multiple slats, with a “dense” 0.5-turn crossover nanoseed recognizing sequences with as low as ∼95% identity. Closer to the reversible temperature, specificity increased up to ∼97% for sparse nanoseeds and ∼98.5% for dense nanoseeds (see Figure 3 from Minev et al.^16^ for characterization of spurious nucleation of the v6.1 linear-growth design used for these experiments).

For detection applications, some other desirable characteristics include the ability to detect shorter sequences, the ability to detect sequences from viral particles in complex biofluids, and the ability to increase signal strength by simultaneous detection of multiple regions. Along these lines, we report the following findings from three separate lines of experimentation: (1) we were able to truncate the capture slats by two binding sites each for the dense nanoseeds to detect a 126 nt ssDNA target sequence (c.f. the full-length 188 nt sequence; Supp. Figure 37); (2) we found that nanoseeds can form and nucleate ribbon assembly from intact M13 phage particles^25^ in biological media such as 10% fetal bovine serum or 2xYT microbial growth media (Supp. Figure 38), and (3) we found that detecting multiple regions on the *E. coli* genome at the same time produced an additive signal (Supp. Figure 39).

### Stochastic Simulations of Ribbon Scission

As exemplified in Method “Sequence design”, computational support is beneficial for designing complex self-assembly processes like 3CR. Computational strategies can likewise be helpful for illustrating the effects of higher-level design choices on the dynamical behavior of 3CR, for example, monomer coordination, toehold length, wobble strength, and wobble arrangement. However, the need to encode binding-site identities of many unique monomers and the stochastic nature of scission render approaches such as molecular dynamics impractical. We thus developed stochastic models at a higher level of abstraction to simulate crisscrossed ribbon scission. In this case, we do not aim for quantitative prediction of ssDNA assembly but rather to provide a framework for qualitatively exploring the parameters at play, especially as the 3CR strategy could in principle be implemented with other monomer types as well.

**Figure 4:**
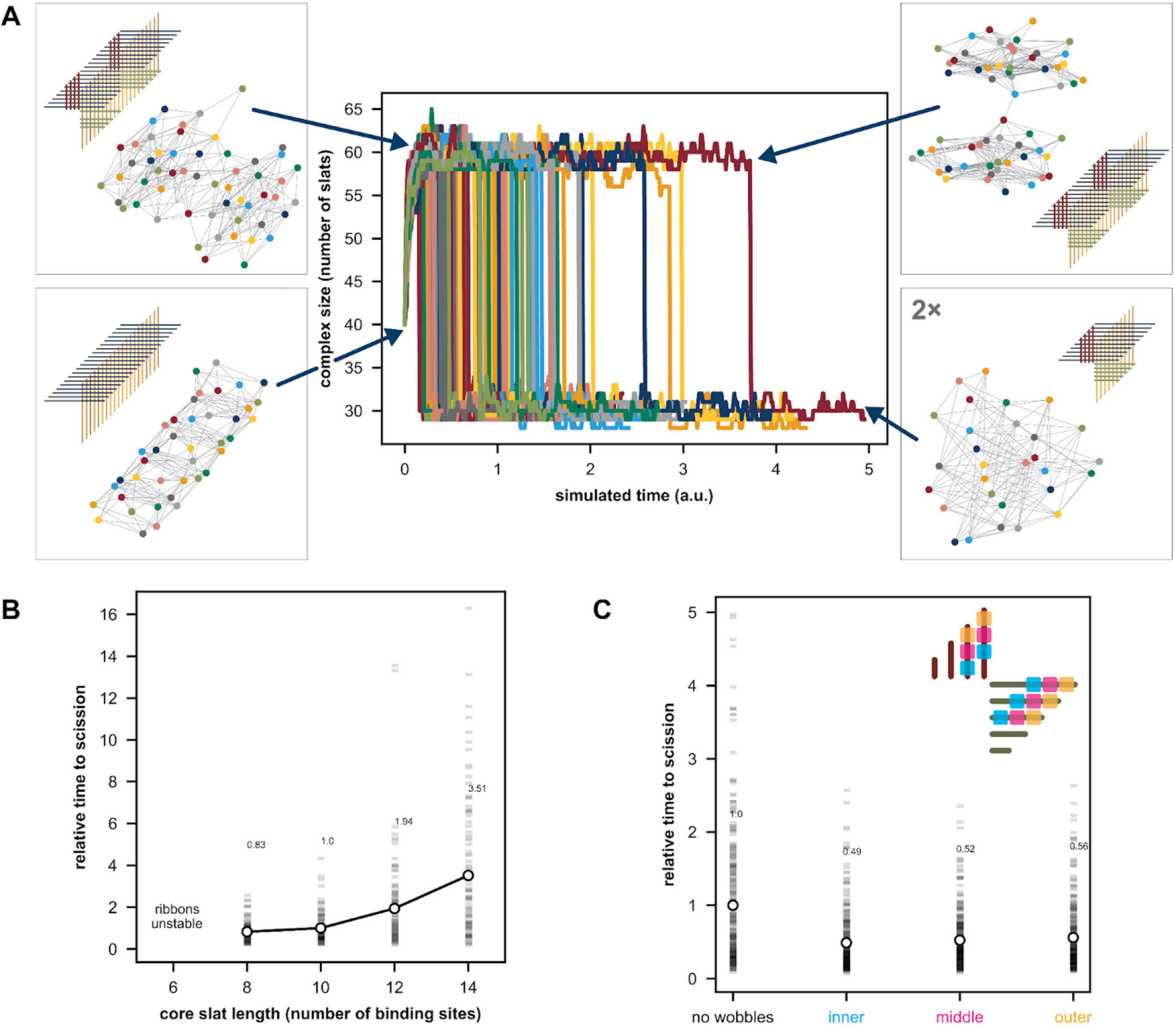
Kappa simulations of scission of a two-repeat ribbon, in the absence of growth, for core slat length 10 (i.e., v5), extension length 5 (i.e., five-segment toeholds), and no wobbles. (A) Plot of 100 simulation trajectories tracking the number of slats in the ribbon. Scission breaks the ribbon into two complexes of comparable size. The four example graphs, depicted in pop-out squares, demonstrate a sequential maturation from the initial ribbon with two repeat-units and no cut-slats bound, followed by increases in complex size until all cut-slats are captured by the extensions, followed by scission. In the graphs, colored circles each represent a single slat, and edges are the bonds between them. (B) Simulations showing mean time to scission versus slat length, corresponding to the ribbon width, with 5-segment toeholds and no wobbles (trajectories for the core slat length 10 shown in (A)). Every data point (white circle) is a mean of 100 simulations, with individual simulations represented as transparent gray rectangles. Time to scission was determined as the sharp decrease in complex size shown in (A) Data-points are annotated with the mean time to scission normalized to that for the core slat length of 10. (C) Kappa simulations with prebound cut-slats (core slat length 10, extension length 5, wobble strength 2/3) showing effect of arrangement of 5 wobble-sites as represented by the different colors. Every data-point is a mean of 300 simulations.

To this end, we used the Kappa platform for rule-based stochastic simulations^21^ (see discussion in Method “Kappa simulation design” and Supp. Figure 40 for details on how monomer geometry was accounted for in this purely graph-based framework). We designed a model consisting of a single ribbon with two repeat units of growth slats, with variable monomer coordination number (“core slat length”), extension length, and strength/arrangement of “wobble” binding sites (i.e., binding sites where growth-slat binding is made weaker than cut-slat binding), and a 100-fold excess of each cut-slat. Running 100 simulations per tested condition, we detected ribbon scission as a sharp decrease in the maximum complex size within the simulation (Figure 4A). We found that increasing the slat length decreases the scission rate due to the larger amount of simultaneous TMSD that needs to take place (Figure 4B). We also explored the changes in scission rate for different extension lengths (Supp. Figure 41A), wobble counts and strengths (Supp. Figure 41B,C), and the different designs with either more cut-x or else more cut-y slats (Supp. Figure 41D). By initializing the model with cut slats prebound to the ribbon and running 300 simulations per condition, we furthermore tested the effect of wobble positions on the scission rate (Figure 4C and Supp. Figure 42).

In addition, to validate our mechanistic understanding of how crisscross ribbon growth and scission occur and aid the interpretation of agarose-gel data, we designed a simple stochastic model that considers linear growth as a continuous process and cutting as single stochastic events occurring at fixed ribbon intervals (Supp. Figure 43). These simulation results are in general alignment with the morphology of the cut-ribbon bands seen in gels throughout this work and can help rationalize to what degree growth and scission respectively are limiting in each case.

## Conclusions

Through the elongation of DNA slats to contain toehold domains, we expanded linear crisscross ribbon assembly to an exponential regime by implementing simultaneous growth and scission and showed how such an approach can be used for the detection of different nucleic acid targets. We also presented a general strategy using rule-based modeling to simulate molecular self-assembly behaviors such as crisscross ribbon scission. This modeling approach can likely be expanded to simulate complex information-bearing interactions such as crosstalk and algorithmic assembly for a broad range of monomer designs. While the implementation in this work acts as a proof of concept of the 3CR strategy, future directions to enable the use of 3CR as a low-cost ultrasensitive diagnostic could involve the improvement of the amplification rate and limit of detection via further sequence optimization to incorporate more wobbles while maintaining fast growth and undetectable spurious nucleation. Furthermore, fluorescence/colorimetric readouts in solution based on the conversion of single-stranded DNA to double-stranded DNA can expand the utility of 3CR as a detection method. The DNA-only cycling-free nature of crisscross ribbon growth and scission could also serve as a basis for designing more complex self-replication behaviors, potentially spawning diverse ribbon morphologies from different seeds and incorporating directed evolution (Supp. Figure 44).

## Materials and Methods

### Sequence Design

Ribbon architectures were designed using scadnano,^26^ and corresponding slat sequences were generated using custom Python scripts. All sequences used in this work are provided in Supp. Table 1. Sequences were generally designed to contain relatively isoenergetic binding sites and minimal self-structure of each slat as assessed using NUPACK.^27,28^ For linear growth, growth-slat sequences for v6.1 from Minev et al.^16^ were used, while for scission growth, growth-slat sequences were optimized as described below. Sequences in the region coupling nuc-x to nuc-y slats were designed to minimize the slat self-structure, have relative isoenergetic binding sites, and keep GC-content within 45–50% and avoid 6-nt repeats. Target sequences were screened in 188-nt windows for self-structure using the NUPACK mfe function, and windows with the lowest self-structure were used to design the corresponding nuc-x slats. For dsDNA targets like lambda and JM109 *E. coli*, the reverse-complement of the window 5-nt upstream of the initial sequence was used to enable the capture of both the “forward” and “reverse” strands.

### Denaturing Polyacrylamide Gel Electrophoresis (PAGE) Purification

DNA oligonucleotides were purchased from Integrated DNA Technologies (IDT) and resuspended in water. Slats of the same type (e.g., x, y, nuc-x) and of the same length were pooled for combined purification. Cut slats and nucleic slats of different lengths were individually purified. Pools were mixed with at least the same volume of 95% formamide, 0.025% (w/v) bromophenol blue, and 5 mM EDTA loading buffer. The SequaGel UreaGel System (National Diagnostics) was used to prepare 15% denaturing PAGE gels in empty plastic 1.5 mm minigel cassettes (Invitrogen Novex). Empty gels were prerun for 1 h at 300 V in 0.5× TBE buffer (45 mM Tris, 45 mM boric acid, 0.78 mM EDTA), and then samples were loaded and run at 300 V for at least 35 min. Bands of the correct molecular weight were excised using UV shadowing, crushed with a pestle, and shaken at 1500 rpm in 500 μL of 1× TE buffer (5 mM Tris 1 mM EDTA) overnight. Extracts were separated by centrifugation in Freeze N’ Squeeze tubes (Bio-Rad, 732-6166); 2.5–3 volumes of 100% ice-cold ethanol and 0.1 volume of 3 M sodium acetate were added to the samples, followed by mixing by inversion and incubation for 15 min at −80 °C. Samples were precipitated in a refrigerated centrifuge, washed twice with 70% ice-cold ethanol, dried in air, and resuspended in water. The final yield was determined by using a Nanodrop 2000c spectrophotometer (Thermo Scientific).

### Assembly Reactions

Assembly reactions were typically performed in 10 μL volumes containing 0.1–1 μM each of purified slats, 12–20 mM MgCl_2_, Tris-EDTA buffer (5 mM Tris 1 mM EDTA, pH 8), 0.01% Tween, and up to 1 nM of the target, and incubated overnight using a PTC-225 Peltier Thermal Cycler (MJ Research) or a Tetrad 2 Peltier thermal cycler (Bio-Rad). Reactions containing DNA targets were first denatured at 85 °C for 5 min followed by a suitable isothermal growth temperature. 65 °C was used instead of 85 °C for RNA targets. Overnight incubations were used as shorter incubations (e.g., 1.5 h as in Supp. Figure 17) did not generate a strong signal in the 3CR system, motivating future directions exploring ways of increasing the amplification rate.

The same reactions were used for Figures 2B, C, and 3C using the design from Supp. Figure 1C. These were performed at 20 mM Mg^2+^, 5 mM Tris, 1 mM EDTA, pH 8.0, 0.01% Tween-20, 65 °C for 5 min and then 48 °C overnight for ∼22 h using 0.1 μM/nuc-slat, 0.15 μM per growth slat, 1 μM/cut slat, and variable target concentrations. Growth only (i.e., linear amplification) versus growth plus scission (i.e., exponential amplification) ribbons at 1 nM p8064 were used for Figure 2. MS2 RNA was purchased from Sigma-Aldrich and diluted in water. JM109 gDNA was prepared as described below, and nuc-x slats for target capture against both forward and reverse strands of “gene 2” from Supp. Figure 39 were used. For Figure 2D, reactions were likewise performed at 20 mM Mg^2+^, 5 mM Tris, 1 mM EDTA, pH 8.0, 0.01% Tween-20, 65 °C for 5 min and then 48 °C overnight for ∼21 h using 0.15–0.25 μM per growth slat (the cy5-labeled growth-x slat being at roughly 50% of the concentration of the other growth slats), 0.05 μM/nuc-slat, 0.15–1 μM per cut-slat, and 100 pM p8064 target.

### Agarose Gel Electrophoresis

Ribbon assembly reactions were characterized by agarose gel electrophoresis. Ultrapure agarose (Life Technologies) was melted in 0.5× TBE buffer (45 mM Tris, 45 mM boric acid, 0.78 mM EDTA, ∼ 0.4× SYBR Gold, 12 mM MgCl_2_) at a concentration of 0.5% (w/v) for linear reaction characterization or 1.5% (w/v) for scission reaction characterization. Gels were covered with aluminum foil during solidification and running to lessen exposure to ambient light; 1–4 μL of samples was combined with 5–10 μL agarose loading buffer (5 mM Tris, 1 mM EDTA, 30% (w/v) glycerol, 0.025% (w/v) xylene cyanol). For reactions containing Cy5-labeled x slats, a loading buffer without xylene cyanol was used to avoid background fluorescence. Electrophoresis was performed at 55–60 V for 2–3.5 h using the Thermo Scientific Owl EasyCast B2 or D3-14 system. Gel images were captured with a GE Typhoon FLA 9500 fluorescent imager set at SYBR-Gold parameters and 300 V and adjusted using FIJI ImageJ^29^ with the “Minimum” and “Maximum” sliders under “Brightness/Contrast” and “Despeckle”.

### Transmission Electron Microscopy

Samples were diluted 1:40 for reactions in Figure 2 and 1:20 for reactions in Supp. Figures 14 and 25 in 12 mM MgCl_2_ 0.7x TE buffer. FCF400-CU-50 grids (Fisher Scientific) were negatively glow-discharged at 15 mA for 25 s in a PELCO easiGlow; 4 μL of the sample was applied to a grid, incubated for 2 min, and wicked off using Whatman paper (Fisher Scientific). 4 μL of 2% aqueous filtered uranyl formate was immediately added and wicked off. Imaging was performed at 80–120 kV on a JEOL JEM 1400 plus microscope. Ribbon lengths were measured by using the “segmented line” tool in FIJI ImageJ.

### JM109 *E. coli* gDNA Preparation

2xYT medium (Fisher Scientific) was inoculated using JM109 stock and cultured on a shaker overnight at 200 rpm and 37 °C. The QIAprep Spin Miniprep kit (Qiagen) was used to lyse the cells and pellet the genomic DNA. Prior to the addition of neutralizing buffer N3 and subsequent steps, the lysate was sonicated using the 1000 bp protocol in an M220 focused ultrasonicator (Covaris). With the current designs, we found that miniprep with sonication (shortening the dsDNA fragment length to enable easier invasion by capture slats, as well as allowing gDNA fragments to pass through the column filter) was necessary for JM109 detection (Supp. Figure 45). The final sample was resuspended in water, and the yield was quantified using a Nanodrop 2000c spectrophotometer (Thermo Scientific).

### Fluorophore Conjugation

In order to aid gel readout of ribbons formed when detecting targets of similar migration (e.g., JM109 *E. coli* gDNA or lambda DNA), we used fluorophore-conjugated x slats. For the 3CR design from Supp. Figure 1C, we purchased one x slat (the top one from the scadnano diagram) with a 1T linker and Cy5 fluorophore on its 3′ end from IDT with HPLC purification. For linear crisscross analysis, we conjugated Atto647 NHS esters (Sigma-Aldrich) to all 12 amino-modified v6.1 y slats (purchased from IDT). 1:50 ratio of PAGE-purified oligonucleotide to dye in 1 M NaHCO_3_ buffer was shaken at 2000 rpm for 2 h at 22 °C in the dark, purified using NAP-5 columns, and concentrated using a Speedvac. The yield was quantified using a Nanodrop 2000c spectrophotometer (Thermo Scientific).

### Kappa Simulation Design

In the rule-based stochastic simulation approach used by the Kappa platform, monomers are conceptualized as agents with a number of sites that represent distinct interaction capabilities such as binding to other agents, allowing for the connection of agents into site graphs. In the graph, the nodes are the agents and the edges are the bonds between agents. A rule can be viewed as a graph-rewrite directive, acting as a mapping of one site graph to another. The specification of rules is related to the specification of the actual molecular associations simply by defining the reaction rates of interactions between specified pairs of binding sites. These rules are then implemented by the simulator as a continuous-time Monte Carlo Gillespie simulation.

A major difference between a site-graph representation and a physical monomer assembly is the lack of information about monomer geometry within the graph. While this allows for intrinsically faster simulation time scales than is possible with approaches such as molecular dynamics^30−33^ and does not require building a purpose-built simulator for new assembly architectures (c.f. xgrow^34^ for square-tile assembly, and SlatTAS^35^ for nonscission crisscross), as far as the graph is concerned, the same binding site on a slat at any location within a crisscross ribbon is equivalent as they are part of the same complex. However, due to the physical dimensions of a given monomer, it is far more likely for two binding sites in close proximity to interact than it is for ones separated by several repeat units of slats. To overcome this constraint, we programmatically generated rules that include a three-point constraint checking for the local environment of any possible intracomplex interaction (Supp. Figure 40). This context ensures that bonds between slats that are already in the ribbon complex can only form if the two slats are in physical proximity to each other by checking that they are connected to each other within two slats.

We note that specific estimates of the kinetics of toehold-mediated cut-slat recruitment are confounded by the choice of the relative intra- and intercomplex on-rates (see discussion in Method “Kappa Simulation Implementation”). Furthermore, for the ssDNA-slat implementation experimentally demonstrated in this work, cut-slat recruitment (in particular cut-y) is likely to be cooperative due to some spatial fixation of extensions by the binding of any one cut-slat and also the creation of a stacking interface that would aid binding. These constraints are specific to the choice of monomer: for example, cut-slat recruitment for scission with origami slats^17^ is less likely to be cooperative due to the higher rigidity of monomers and greater independence of the binding sites.

### Kappa Simulation Implementation

Custom Python scripts were used to systematically generate ribbon descriptions and rules for Kappa simulation input files (available at https://github.com/aersh/3cr). Representations of crisscross ribbons of different widths with different extension lengths were programmatically generated by using matrix operations. All possible combinations of the three-point constraint for intracomplex rules were generated through a series of conditional statements going through all possible traversals for binding of both growth-slats to each other and of cut-slats to growth-slats. For simulations without cut slats prebound (as in Figure 4A, B) off-rates were set at 1, intercomplex on-rate at 0.04, and intracomplex on-rate at 40. The ratio α of intra- to intercomplex (1 M free strand concentration) on-rate corresponds to the loss of positional entropy upon capture of free monomers from solution. Typically, for DNA helix initiation, an α of 20 would be expected; however, a low value of α yielded simulations with multiple cut slats bound simultaneously to the same extension, which would be unrealistic in a physical system, while with significantly higher values of α, the scission rate was dependent solely on the toehold length. Thus, we settled on an α of 1000 as it yielded the qualitatively closest results to experimental reality. The need for these assumptions about an α value is mitigated by designing a model with prebound cut-slats where only intracomplex interactions are possible. Furthermore, the specific α value for an experimental system would depend greatly on the specific choice of monomer (e.g., up to a limit of 10^8^ for succinic acid ring closure to succinic anhydride^36^). As a result, our choice of α of 1000 is taken as a representative of a generic monomer type and is not meant to strictly correspond to the ssDNA slats used experimentally in this work. The ratio between the on-rate and off-rate was determined empirically by simulations such that the ribbon complex itself was relatively stable throughout the duration of the simulations. For simulations with cut slats prebound (as in Figure 4C), the intercomplex on-rate was set at 0.05 and the off-rate at 0.8 to ensure that cut slats remain bound throughout. On-rates for wobble binding sites were scaled by a factor of 2/3 and off-rates by a factor of 1.5. KaSim v4.1 was used to run simulations locally on a 2019 iMac.

## Supporting Information Available

Scadnano design schematics; additional agarose gel and negative-stain TEM data; and Kappa simulation constraints and simulated data (PDF)
Spreadsheet of all DNA sequences used in this work (XLSX)
Animation illustrating process of crisscross ribbon scission, with ribbon growth omitted for clarity. Slat types are colored as in Figure 2 (MP4)

## References

[ref1] Rothemund P. W. Folding DNA to Create Nanoscale Shapes and Patterns. Nature 2006, 440 (7082), 297–302. 10.1038/nature04586. 16541064

[ref2] Douglas S. M.; Dietz H.; Liedl T.; Hogberg B.; Graf F.; Shih W. M. Self-Assembly of DNA into Nanoscale Three-Dimensional Shapes. Nature 2009, 459 (7245), 414–418. 10.1038/nature08016. 19458720 PMC2688462

[ref3] Evans C. G.; Winfree E. Physical Principles for DNA Tile Self-Assembly. Chem. Soc. Rev. 2017, 46 (12), 3808–3829. 10.1039/C6CS00745G. 28489096

[ref4] Mohammed A. M.; Schulman R. Directing Self-Assembly of DNA Nanotubes Using Programmable Seeds. Nano Lett. 2013, 13 (9), 4006–4013. 10.1021/nl400881w. 23919535

[ref5] Zhang Y.; Reinhardt A.; Wang P.; Song J.; Ke Y. Programming the Nucleation of DNA Brick Self-Assembly with a Seeding Strand. Angew. Chem., Int. Ed. Engl. 2020, 59 (22), 8594–8600. 10.1002/anie.201915063. 32043698

[ref6] Dirks R. M.; Pierce N. A. Triggered Amplification by Hybridization Chain Reaction. Proc. Natl. Acad. Sci. U. S. A. 2004, 101 (43), 15275–15278. 10.1073/pnas.0407024101. 15492210 PMC524468

[ref7] Ang Y. S.; Yung L.-Y. L. Rational Design of Hybridization Chain Reaction Monomers for Robust Signal Amplification. Chem. Commun. 2016, 52 (22), 4219–4222. 10.1039/C5CC08907G. 26912178

[ref8] Barish R. D.; Schulman R.; Rothemund P. W. K.; Winfree E. An Information-Bearing Seed for Nucleating Algorithmic Self-Assembly. Proc. Natl. Acad. Sci. U. S. A. 2009, 106 (15), 6054–6059. 10.1073/pnas.0808736106. 19321429 PMC2660060

[ref9] Schulman R.; Winfree E. Programmable Control of Nucleation for Algorithmic Self-Assembly. SIAM Journal on Computing 2010, 39 (4), 1581–1616. 10.1137/070680266.

[ref10] Jacobs W. M.; Reinhardt A.; Frenkel D. Rational Design of Self-Assembly Pathways for Complex Multicomponent Structures. Proc. Natl. Acad. Sci. U. S. A. 2015, 112 (20), 6313–6318. 10.1073/pnas.1502210112. 25941388 PMC4443370

[ref11] Reinhardt A.; Ho C. P.; Frenkel D. Effects of Co-Ordination Number on the Nucleation Behaviour in Many-Component Self-Assembly. Faraday Discuss. 2016, 186, 215–228. 10.1039/C5FD00135H. 26762705

[ref12] Winfree E. Algorithmic Self-Assembly of DNA: Theoretical Motivations and 2D Assembly Experiments. J. Biomol. Struct. Dyn. 2000, 17 (Suppl 1), 263–270. 10.1080/07391102.2000.10506630. 22607433

[ref13] Woods D.; Doty D.; Myhrvold C.; Hui J.; Zhou F.; Yin P.; Winfree E. Diverse and Robust Molecular Algorithms Using Reprogrammable DNA Self-Assembly. Nature 2019, 567 (7748), 366–372. 10.1038/s41586-019-1014-9. 30894725

[ref14] Schulman R.; Yurke B.; Winfree E. Robust Self-Replication of Combinatorial Information via Crystal Growth and Scission. Proc. Natl. Acad. Sci. U. S. A. 2012, 109 (17), 6405–6410. 10.1073/pnas.1117813109. 22493232 PMC3340064

[ref15] He X.; Sha R.; Zhuo R.; Mi Y.; Chaikin P. M.; Seeman N. C. Exponential Growth and Selection in Self-Replicating Materials from DNA Origami Rafts. Nat. Mater. 2017, 16 (10), 993–997. 10.1038/nmat4986. 28920942

[ref16] Minev D.; Wintersinger C. M.; Ershova A.; Shih W. M. Robust Nucleation Control via Crisscross Polymerization of Highly Coordinated DNA Slats. Nat. Commun. 2021, 12 (1), 1741 10.1038/s41467-021-21755-7. 33741912 PMC7979912

[ref17] Wintersinger C. M.; Minev D.; Ershova A.; Sasaki H. M.; Gowri G.; Berengut J. F.; Corea-Dilbert F. E.; Yin P.; Shih W. M. Multi-Micron Crisscross Structures Grown from DNA-Origami Slats. Nat. Nanotechnol. 2023, 18, 281–289. 10.1038/s41565-022-01283-1. 36543881 PMC10818227

[ref18] Zhang D. Y.; Seelig G. Dynamic DNA Nanotechnology Using Strand-Displacement Reactions. Nat. Chem. 2011, 3 (2), 103–113. 10.1038/nchem.957. 21258382

[ref19] Srinivas N.; Ouldridge T. E.; Šulc P.; Schaeffer J. M.; Yurke B.; Louis A. A.; Doye J. P. K.; Winfree E. On the Biophysics and Kinetics of Toehold-Mediated DNA Strand Displacement. Nucleic Acids Res. 2013, 41 (22), 10641–10658. 10.1093/nar/gkt801. 24019238 PMC3905871

[ref20] Danos V.; Feret J.; Fontana W.; Harmer R.; Krivine J. Rule-Based Modelling of Cellular Signalling. In CONCUR 2007 – Concurrency Theory; Caires L., Vasconcelos V. T., Eds.; Lecture Notes in Computer Science; Springer: Berlin, Heidelberg, 2007; pp 17–41, 10.1007/978-3-540-74407-8_3.

[ref21] Boutillier P.; Maasha M.; Li X.; Medina-Abarca H. F.; Krivine J.; Feret J.; Cristescu I.; Forbes A. G.; Fontana W. The Kappa Platform for Rule-Based Modeling. Bioinformatics 2018, 34 (13), i583–i592. 10.1093/bioinformatics/bty272. 29950016 PMC6022607

[ref22] Högberg B.; Liedl T.; Shih W. M. Folding DNA Origami from a Double-Stranded Source of Scaffold. J. Am. Chem. Soc. 2009, 131 (26), 9154–9155. 10.1021/ja902569x. 19566089 PMC2724999

[ref23] Süß B.; Flekna G.; Wagner M.; Hein I. Studying the Effect of Single Mismatches in Primer and Probe Binding Regions on Amplification Curves and Quantification in Real-Time PCR. J. Microbiol. Methods 2009, 76 (3), 316–319. 10.1016/j.mimet.2008.12.003. 19135484

[ref24] Zimmermann F.; Urban M.; Krüger C.; Walter M.; Wölfel R.; Zwirglmaier K. In Vitro Evaluation of the Effect of Mutations in Primer Binding Sites on Detection of SARS-CoV-2 by RT-QPCR. Journal of Virological Methods 2022, 299, 114352 10.1016/j.jviromet.2021.114352. 34748815 PMC8570391

[ref25] Nickels P. C.; Ke Y.; Jungmann R.; Smith D. M.; Leichsenring M.; Shih W. M.; Liedl T.; Högberg B. DNA Origami Structures Directly Assembled from Intact Bacteriophages. Small 2014, 10 (9), 1765–1769. 10.1002/smll.201303442. 24532395

[ref26] Doty D.; Lee B. L.; Stérin T. Scadnano: A Browser-Based, Scriptable Tool for Designing DNA Nanostructures. In 26th International Conference on DNA Computing and Molecular Programming (DNA 26); Geary C., Patitz M. J., Eds.; Leibniz International Proceedings in Informatics (LIPIcs); Schloss Dagstuhl–Leibniz-Zentrum für Informatik: Dagstuhl, Germany, 2020; Vol. 174, p 9:1–9:17, 10.4230/LIPIcs.DNA.2020.9.

[ref27] Zadeh J. N.; Steenberg C. D.; Bois J. S.; Wolfe B. R.; Pierce M. B.; Khan A. R.; Dirks R. M.; Pierce N. A. NUPACK: Analysis and Design of Nucleic Acid Systems. J. Comput. Chem. 2011, 32 (1), 170–173. 10.1002/jcc.21596. 20645303

[ref28] Fornace M. E.; Huang J.; Newman C. T.; Porubsky N. J.; Pierce M. B.; Pierce N. A. NUPACK: Analysis and Design of Nucleic Acid Structures, Devices, and Systems ChemRxiv 2022, 10.26434/chemrxiv-2022-xv98l, November 10 (accessed 2023–12–03).

[ref29] Schindelin J.; Arganda-Carreras I.; Frise E.; Kaynig V.; Longair M.; Pietzsch T.; Preibisch S.; Rueden C.; Saalfeld S.; Schmid B.; et al. Fiji: An Open-Source Platform for Biological-Image Analysis. Nat. Methods 2012, 9 (7), 676–682. 10.1038/nmeth.2019. 22743772 PMC3855844

[ref30] Ouldridge T. E.; Louis A. A.; Doye J. P. K. Structural, Mechanical, and Thermodynamic Properties of a Coarse-Grained DNA Model. J. Chem. Phys. 2011, 134 (8), 085101 10.1063/1.3552946. 21361556

[ref31] Šulc P.; Romano F.; Ouldridge T. E.; Rovigatti L.; Doye J. P. K.; Louis A. A. Sequence-Dependent Thermodynamics of a Coarse-Grained DNA Model. J. Chem. Phys. 2012, 137 (13), 135101 10.1063/1.4754132. 23039613

[ref32] Poppleton E.; Romero R.; Mallya A.; Rovigatti L.; Šulc P. OxDNA.Org: A Public Webserver for Coarse-Grained Simulations of DNA and RNA Nanostructures. Nucleic Acids Res. 2021, 49 (W1), W491–W498. 10.1093/nar/gkab324. 34009383 PMC8265093

[ref33] Maffeo C.; Aksimentiev A. MrDNA: A Multi-Resolution Model for Predicting the Structure and Dynamics of DNA Systems. Nucleic Acids Res. 2020, 48 (9), 5135–5146. 10.1093/nar/gkaa200. 32232413 PMC7229838

[ref34] DNA Lab: Xgrow Tile Assembly Simulator. https://www.dna.caltech.edu/Xgrow/ (accessed Sep 14, 2023).

[ref35] Doty D.; Fleming H.; Hader D.; Patitz M. J.; Vaughan L. A. Accelerating Self-Assembly of Crisscross Slat Systems. In 29th International Conference on DNA Computing and Molecular Programming (DNA 29); Leibniz International Proceedings in Informatics (LIPIcs); 2023; Vol. 276, pp 7:1–7:23, 10.4230/LIPIcs.DNA.29.7.

[ref36] Page M. I.; Jencks W. P. Entropic Contributions to Rate Accelerations in Enzymic and Intramolecular Reactions and the Chelate Effect. Proc. Natl. Acad. Sci. U. S. A. 1971, 68 (8), 1678–1683. 10.1073/pnas.68.8.1678. 5288752 PMC389269

