# Supplementary material for: Enzyme-Free Exponential Amplification via Growth and Scission of Crisscross Ribbons from Single-Stranded DNA Components
Source: J Am Chem Soc. 2023 Dec 22;146(1):218–27. doi: 10.1021/jacs.3c08205 (PMC10785819; doi:10.1021/jacs.3c08205)
Supplement: Supplementary file 1 — ja3c08205_si_001.pdf [file ja3c08205_si_001.pdf]

Supporting information for:

Enzyme-free exponential amplification via growth and scission of crisscross ribbons from single-stranded DNA components

Anastasia Ershova[1,2,3,#], Dionis Minev[1,2,3,5,#], F. Eduardo Corea-Dilbert[1,6], Devon Yu[1], Jie Deng[1,2,3], Walter Fontana[4], and William M. Shih[1,2,3,*]

[1]Department of Cancer Biology, Dana-Farber Cancer Institute, Boston, MA, USA
[2]Wyss Institute for Biologically Inspired Engineering at Harvard University, Boston, MA, USA
[3]Department of Biological Chemistry and Molecular Pharmacology, Harvard Medical School, Boston, MA, USA
[4]Department of Systems Biology, Harvard Medical School, Boston, MA, USA
[5]Current address: CATALOG, Boston, MA, USA
[6]Current address: Geisel School of Medicine at Dartmouth, Hanover, NH, USA
[#]These authors contributed equally: Anastasia Ershova, Dionis Minev
*e-mail: William_Shih@dfci.harvard.edu

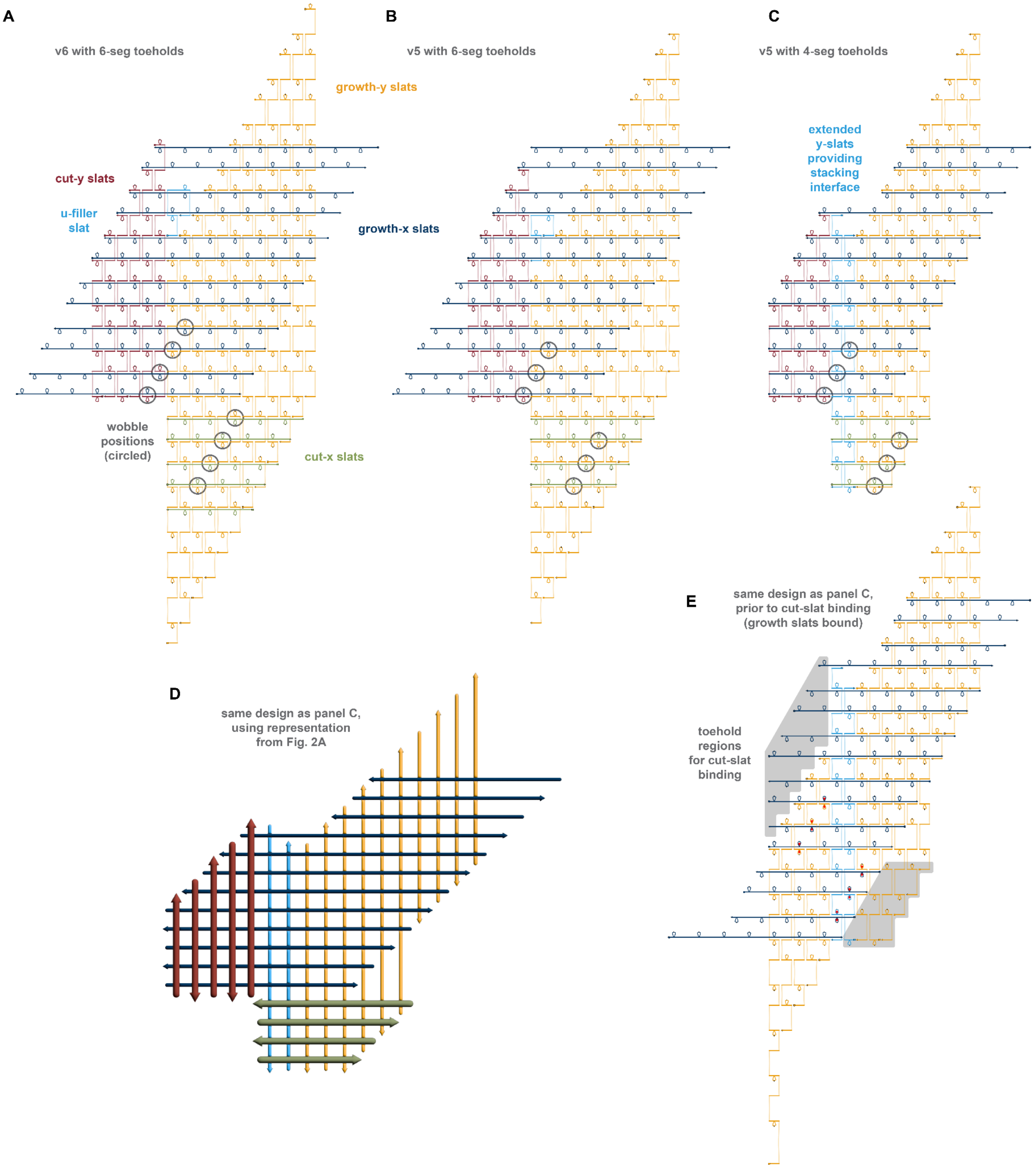

Supplementary Figure 1: scadnano designs of the 3CR system. Segments without insertions are 5 nt, segments with insertions are 6 nt, yielding 11 bp/turn overall. **A**, v6 design with 6-segment toeholds for cut-slats and 8 wobbles. **B**, v5 design with 6-segment toeholds derived from **A** by removing the two middle binding sites of each growth-slat. **C**, v5 design derived from **B** by truncating growth-slats to only contain binding sites for 4-segment toeholds, and lengthening two growth-y slats to provide stacking interface instead of using a u-filler (as shown

in light blue). Unless otherwise noted, design **B** was used as the default in this work. The “4seg u-filler” design (e.g. as in Supplementary Figures 14C and D) uses growth-slats as in **C** except the light blue slats are shorter to allow for the u-filler from **B** to bind instead. Note some regions of the slats remain unpaired, and are necessary to link the toeholds to the core of the growth slats. **D**, Representation of design from **C** using abstraction from Figure 2A. Note this is identical to the design in Figure 2A, except the extended y-slats are highlighted in light blue. **E**, Design from **C** with growth slats bound instead of cut slats, highlighting toehold positions and wobbles (red).

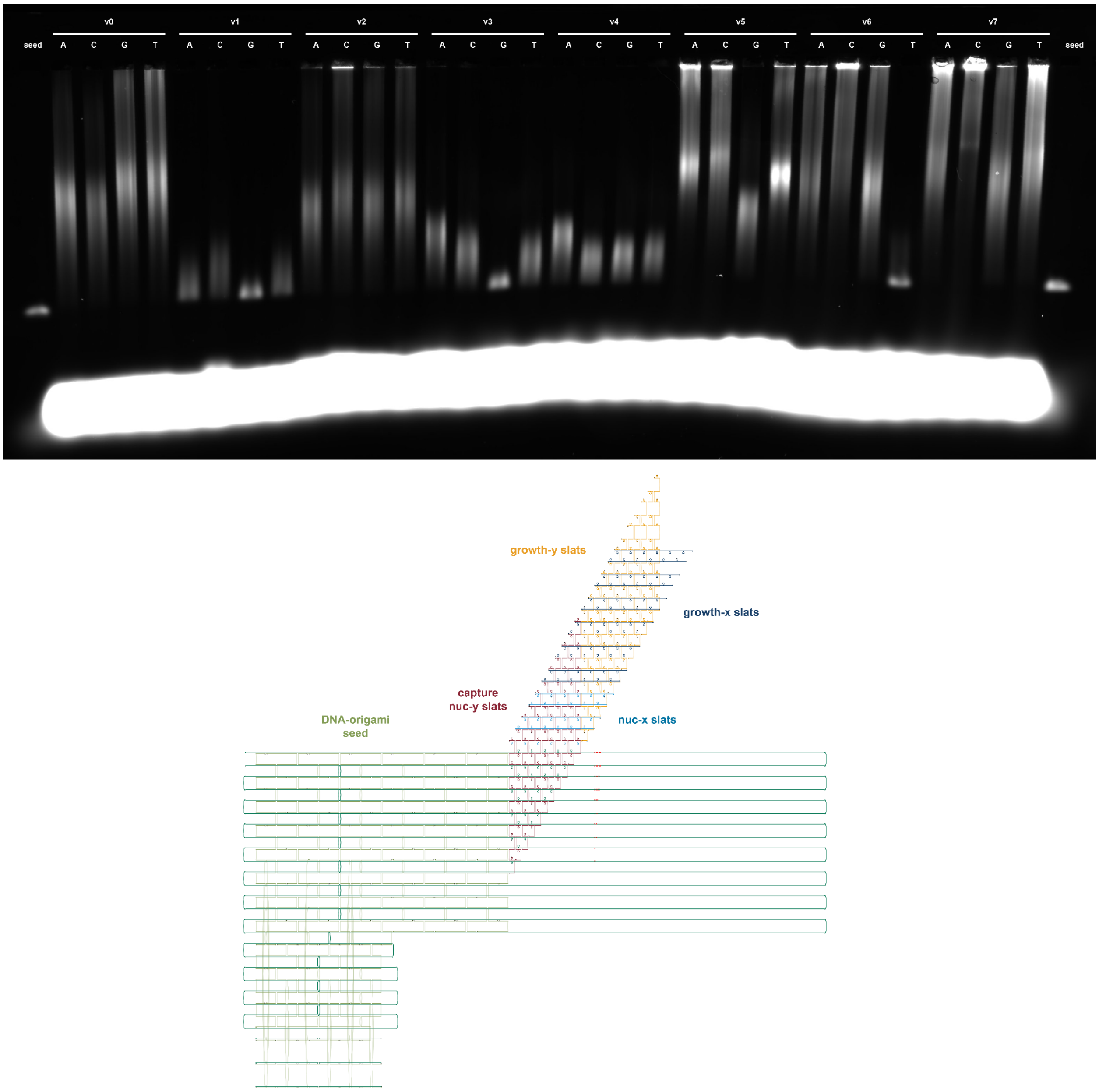

Supplementary Figure 2: Top: screen of 8 different v6 growth-sequence variants with either A, C, G, or T at each of 8 wobble locations shown in Supplementary Figure 1A (C being the Watson-Crick base-pair). v7 with "T" was chosen for further screening of extension sequences, as the slower gel migration suggests the fastest growth rate out of all the wobble conditions tested. Reactions were performed at 14 mM Mg^{2+}, 4 mM Tris, 0.8 mM EDTA, pH 8.0, 0.01% Tween-20, 46 °C overnight using 0.5 µM/slat and 1 nM DNA-origami seed[1]. Bottom: scadnano diagram of DNA-origami seed from Minev et al (Supplementary Figure 13)[1] coupled with v6 growth. Please refer to the main text results section "Detection of nucleic acid targets" for details on how crisscross growth is coupled to different seeds. Note: unlike the nanoseed designs presented elsewhere in this work, initial engagement with the target is achieved through "nuc-y" slats instead of "nuc-x" slats for this DNA-origami seed.

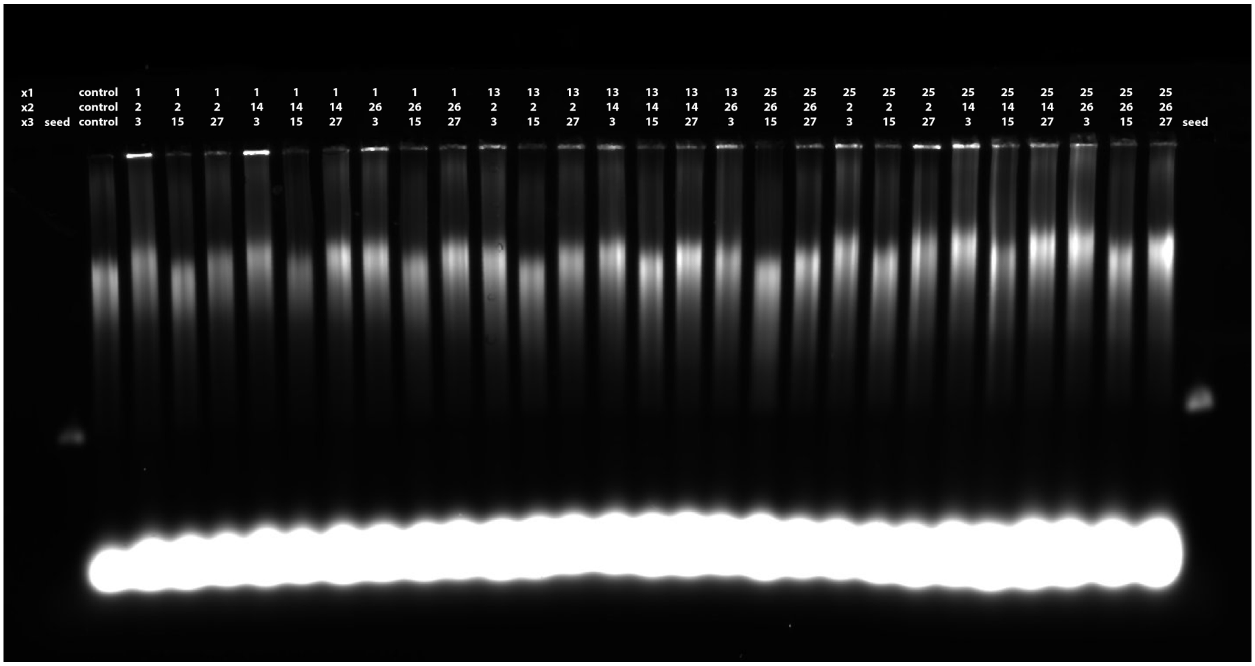

Supplementary Figure 3: Screen of 6seg extensions on growth slats x1–3 for v6 design from Supplementary Figure 2. Combination 13/14/27 was chosen to proceed to the next round of screening. Reactions were performed at 14 mM Mg^{2+}, 4 mM Tris, 0.8 mM EDTA, pH 8.0, 0.01% Tween-20, 46 °C overnight using 0.5 μM/slat and 1 nM DNA-origami seed[1].

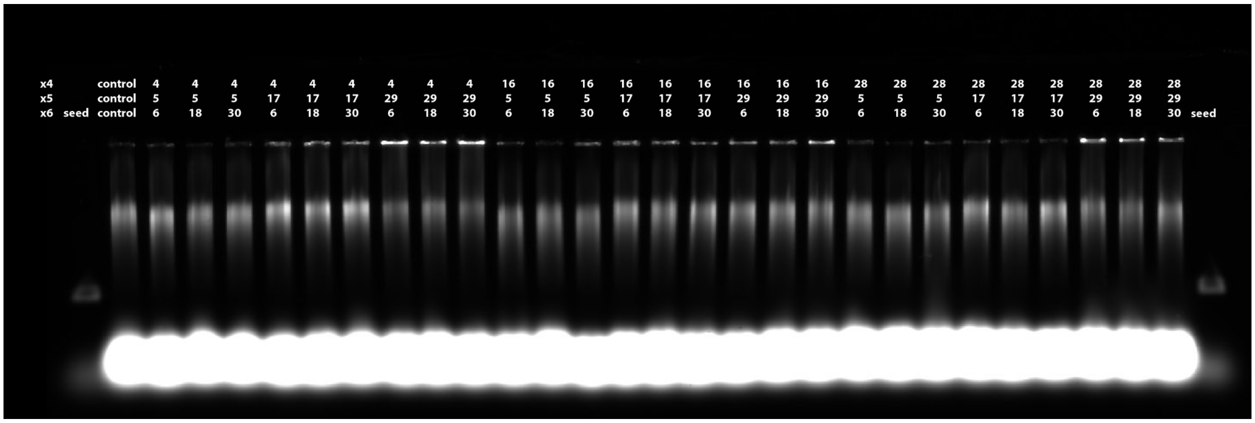

Supplementary Figure 4: Screen of 6seg extensions on growth slats x4–6 for v6 design from Supplementary Figure 3. Combination 13/14/27/28/17/6 was chosen to proceed to the next round of screening. Reactions were performed at 14 mM Mg^{2+}, 4 mM Tris, 0.8 mM EDTA, pH 8.0, 0.01% Tween-20, 46 °C overnight using 0.5 μM/slat and 1 nM DNA-origami seed[1].

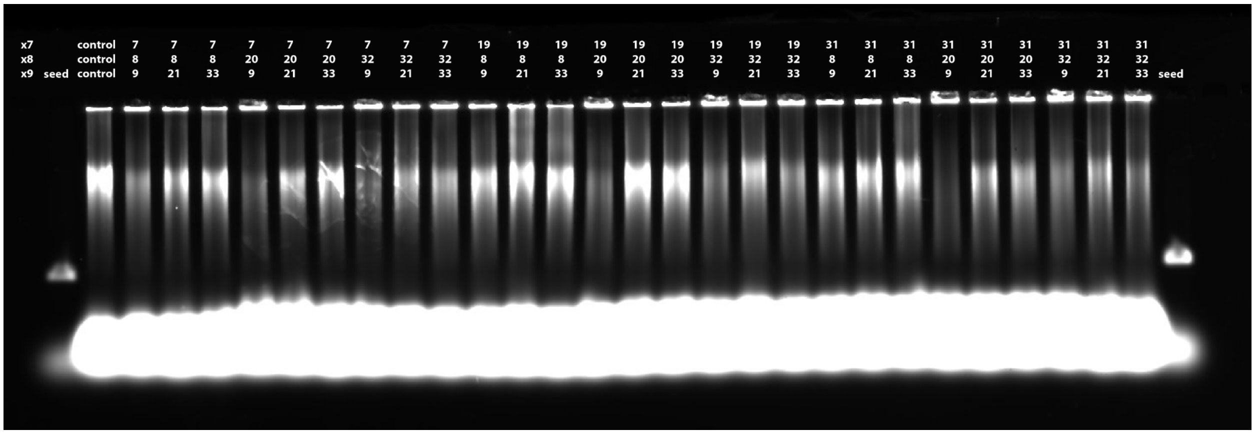

Supplementary Figure 5: Screen of 6seg extensions on growth slats x7–9 for v6 design from Supplementary Figure 4. Combination 13/14/27/28/17/6/19/20/21 was chosen to proceed to the next round of screening. Reactions were performed at 14 mM Mg^{2+}, 4 mM Tris, 0.8 mM EDTA, pH 8.0, 0.01% Tween-20, 46 °C overnight using 0.5 µM/slat and 1 nM DNA-origami seed[1].

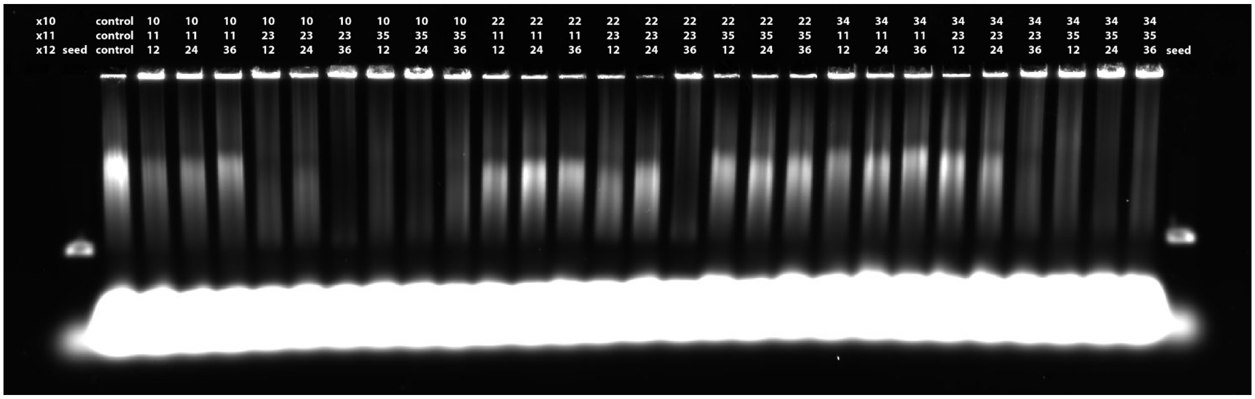

Supplementary Figure 6: Screen of 6seg extensions on growth slats x10–12 for v6 design from Supplementary Figure 5. Combination 13/14/27/28/17/6/19/20/21/34/11/36 was chosen to proceed to the next round of screening. Reactions were performed at 14 mM Mg^{2+}, 4 mM Tris, 0.8 mM EDTA, pH 8.0, 0.01% Tween-20, 46 °C overnight using 0.5 µM/slat and 1 nM DNA-origami seed[1].

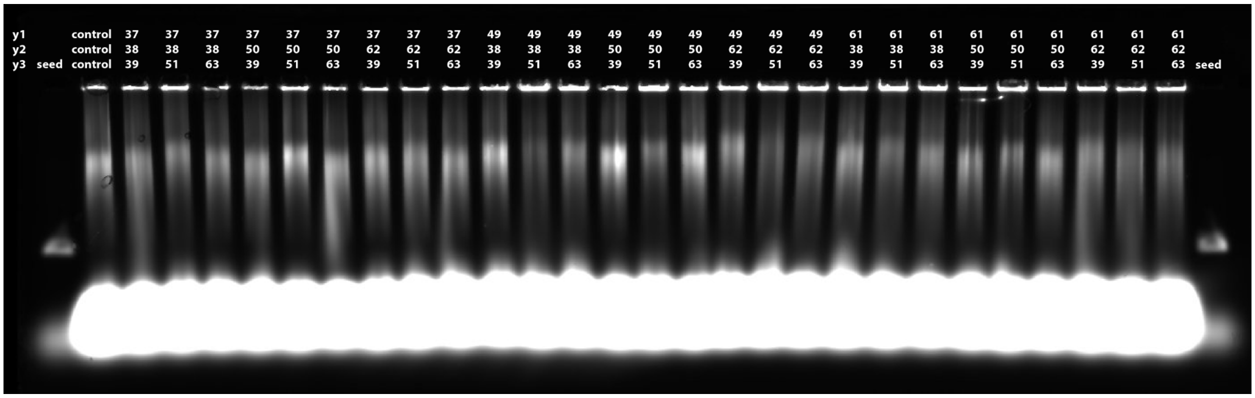

Supplementary Figure 7: Screen of 6seg extensions on growth slats y1–3 for v6 design from Supplementary Figure 6. Combination 13/14/27/28/17/6/19/20/21/34/11/36/37/50/51 was chosen to proceed to the next round of screening. Reactions were performed at 14 mM Mg^{2+}, 4 mM Tris, 0.8 mM EDTA, pH 8.0, 0.01% Tween-20, 46 °C overnight using 0.5 µM/slat and 1 nM DNA-origami seed[1].

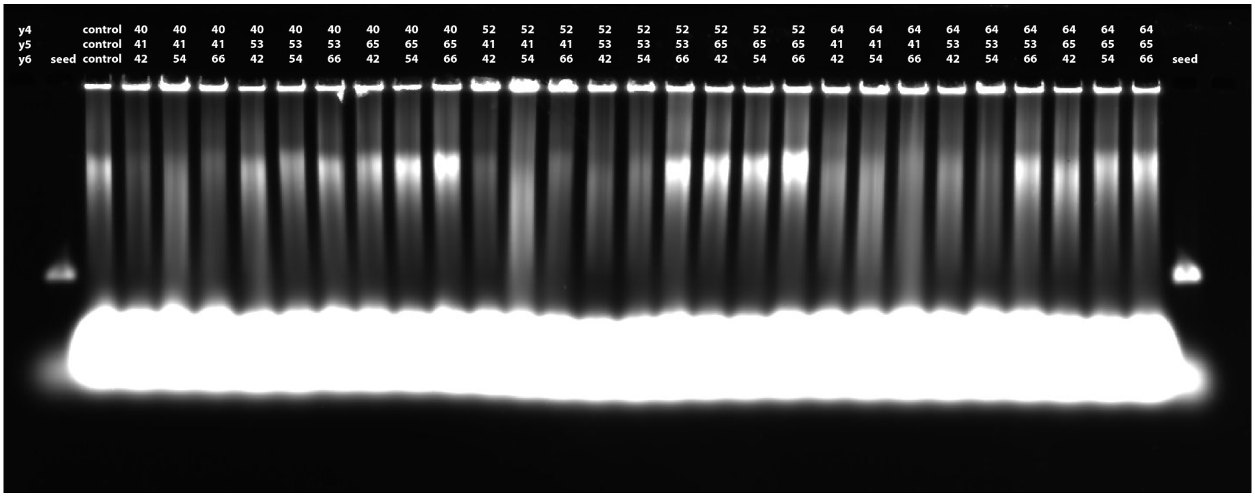

Supplementary Figure 8: Screen of 6seg extensions on growth slats y4–6 for v6 design from Supplementary Figure 7. Combination 13/14/27/28/17/6/19/20/21/34/11/36/37/50/51/52/65/66 was chosen to proceed to the next round of screening. Reactions were performed at 14 mM Mg^{2+}, 4 mM Tris, 0.8 mM EDTA, pH 8.0, 0.01% Tween-20, 46 °C overnight using 0.5 µM/slat and 1 nM DNA-origami seed[1].

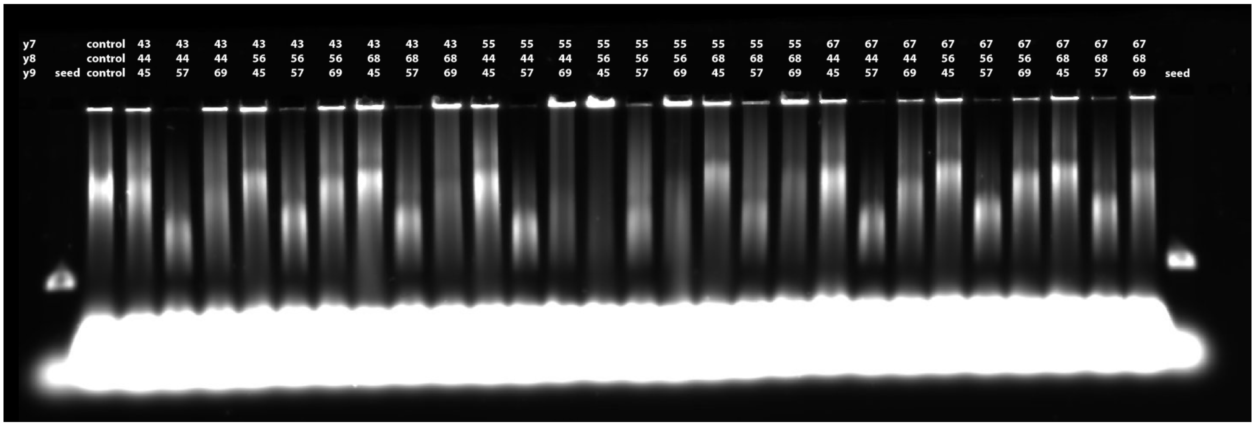

Supplementary Figure 9: Screen of 6seg extensions on growth slats y7–9 for v6 design from Supplementary Figure 8.

Combination 13/14/27/28/17/6/19/20/21/34/11/36/37/50/51/52/65/66/43/68/45 was chosen to proceed to the next round of screening. Reactions were performed at 14 mM Mg^{2+}, 4 mM Tris, 0.8 mM EDTA, pH 8.0, 0.01% Tween-20, 46 °C overnight using 0.5 µM/slat and 1 nM DNA-origami seed[1].

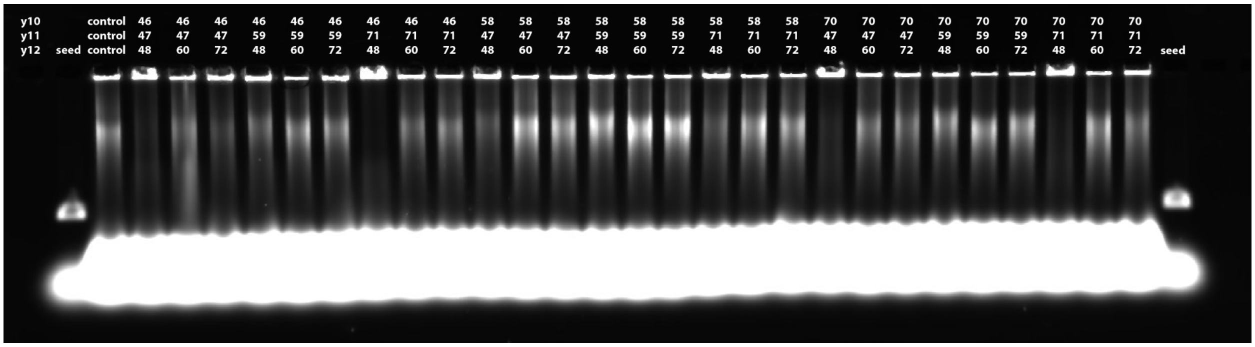

Supplementary Figure 10: Screen of 6seg extensions on growth slats y10–12 for v6 design from Supplementary Figure 9.

Combination 13/14/27/28/17/6/19/20/21/34/11/36/37/50/51/52/65/66/43/68/45/58/59/60 was chosen to proceed to the next round of screening. Reactions were performed at 14 mM Mg^{2+}, 4 mM Tris, 0.8 mM EDTA, pH 8.0, 0.01% Tween-20, 46 °C overnight using 0.5 µM/slat and 1 nM DNA-origami seed[1].

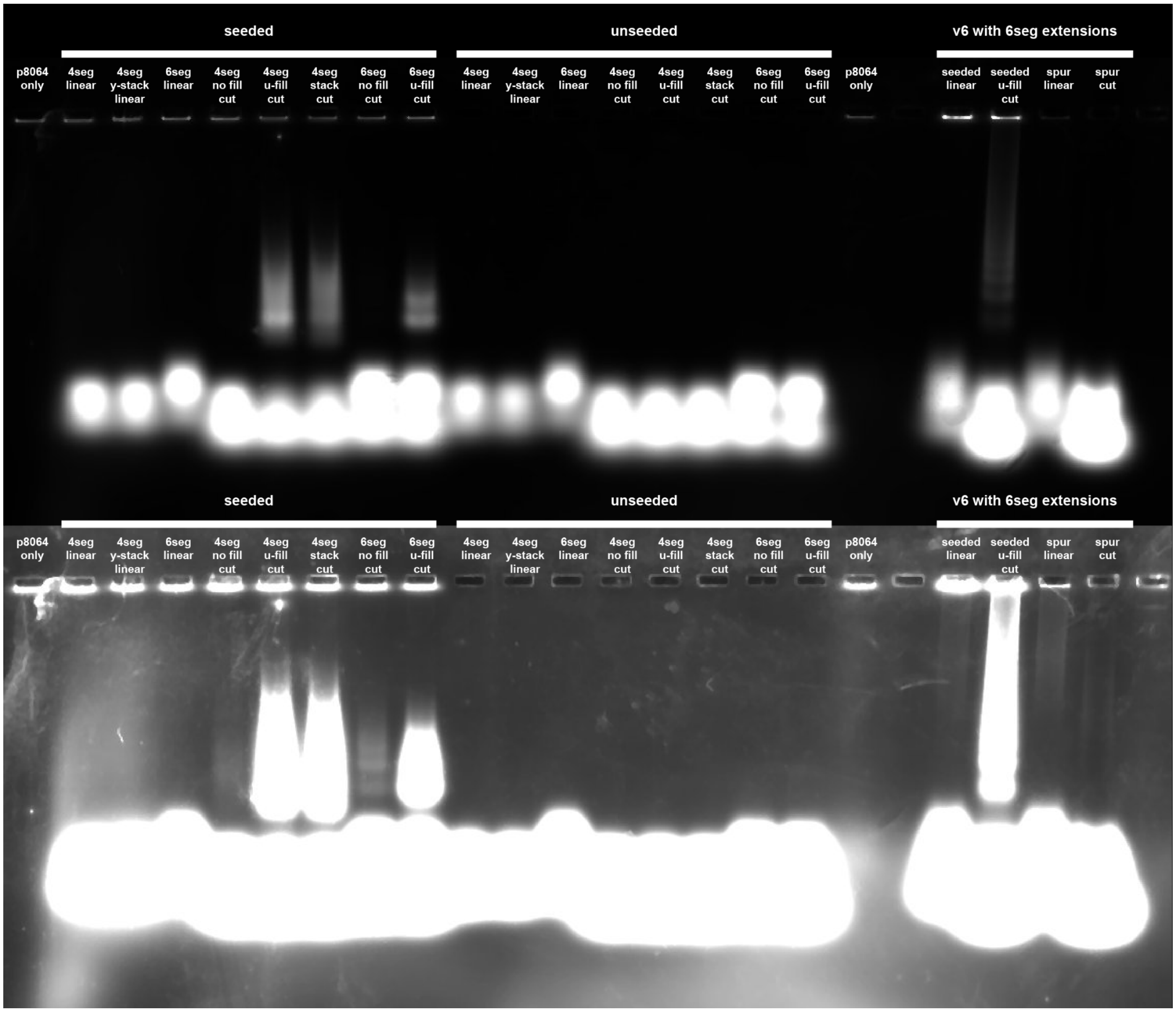

Supplementary Figure 11: Left: v5 3CR scission observed with either full-length growth-slats (with 6-segment extensions as in Supplementary Figure 1B), truncated growth-slats (with 4-segment extensions), or truncated growth-slats with y-stacking and 4-segment extensions (as in Supplementary Figure 1C). Under these conditions, scission with 4-segment extensions appeared to have greater amplification, with comparable rates for the u-fill and stack designs. This is likely due to faster ribbon growth with the shorter extensions, as the 6-segment designs appear to have more complete scission (sharper, faster-migrating bands), and more scission without any fillers/stacking. Right: comparison to v6 scission (as in Supplementary Figure 1A), which showed slower rate of scission and more spurious nucleation. Top and bottom are images of the same gel at different contrasts. Reactions were performed at 20 mM Mg^{2+}, 5 mM Tris, 1 mM EDTA, pH 8.0, 0.01% Tween-20, 85 °C for 5 minutes then 48 °C overnight for ~18 hours using 0.1 μM/nuc-slat, 0.15 μM per growth- and u-fill slat, 1 μM/cut-slat, and 1 nM p8064 target.

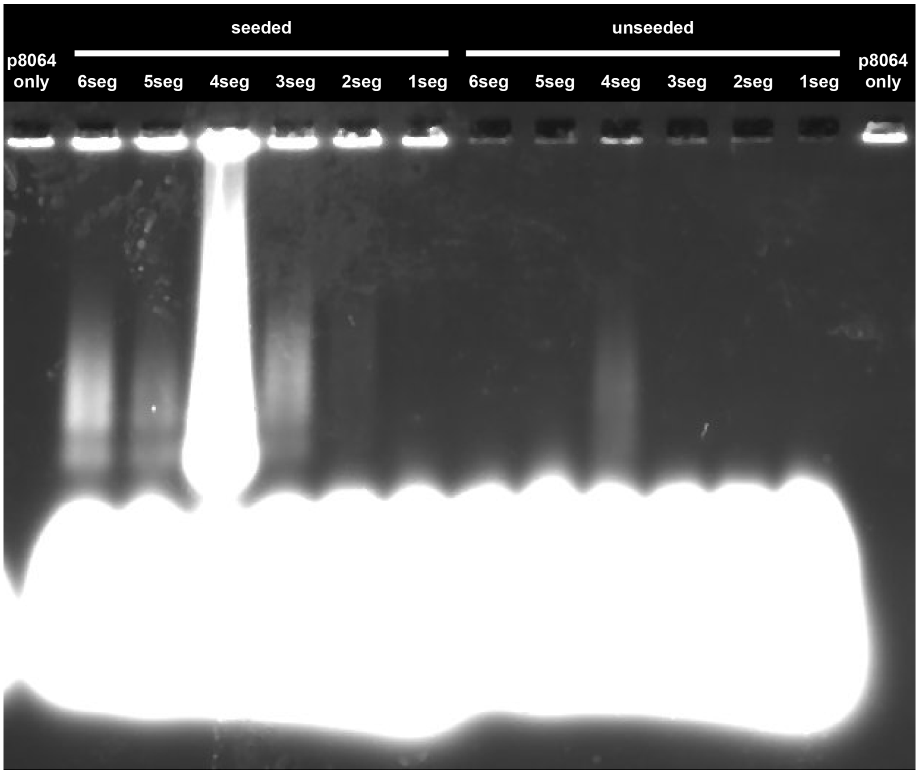

Supplementary Figure 12: Screen of unpurified cut-slats that bind to 1, 2, 3, 4, 5 or 6 segments from the full-length 6seg extensions of v5 3CR. Note longer cut-slats are more likely to have truncated by-products of oligo synthesis that may compete with full-length binding. Reactions were performed at 20 mM Mg^{2+}, 5 mM Tris, 1 mM EDTA, pH 8.0, 0.01% Tween-20, 85 °C for 5 minutes then 48 °C overnight for ~21 hours using 0.2 μM/growth-slat, 0.2 μM/u-filler, 0.1 μM/nuc-slat, 1 μM/cut-slat, and 1 nM p8064 target.

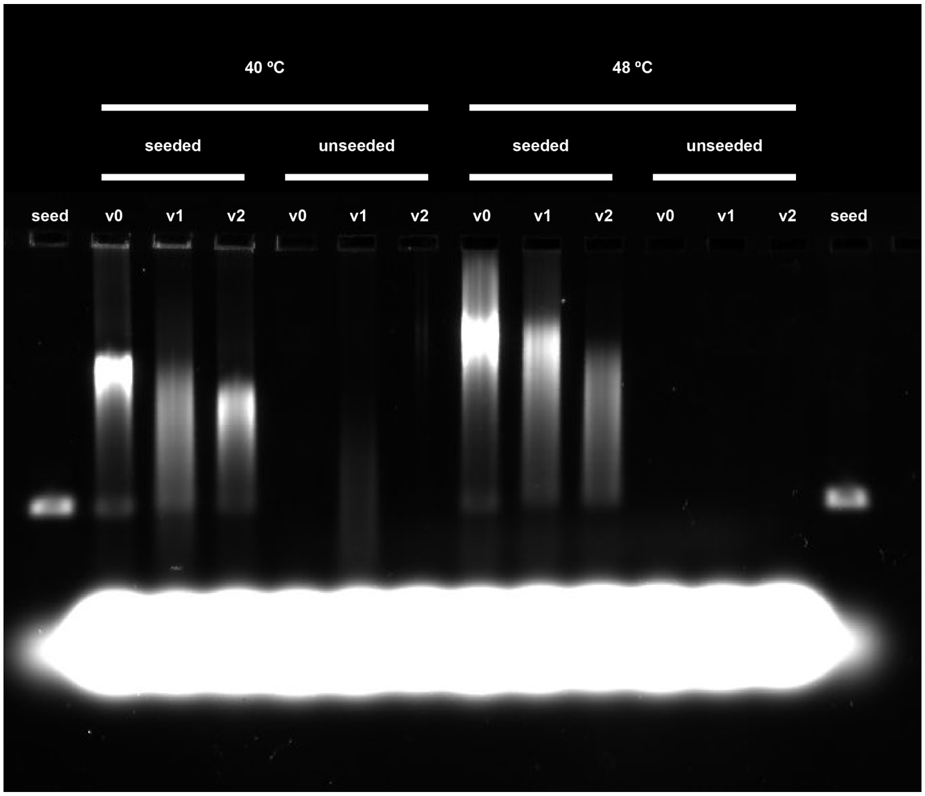

Supplementary Figure 13: Screening of seeded and spurious linear growth for three new sets of v5 sequences containing 6 wobbles and 4-segment extensions. Even though v1 grows more slowly than v0, it still has more detectable spurious nucleation at 40 °C. Reactions were performed at 16 mM Mg^{2+}, 5 mM Tris, 1 mM EDTA, pH 8.0, 40/48 °C overnight using 0.25 μM/nuc-slat, 0.85 μM/growth-slat and 1 nM DNA-origami seed[1].

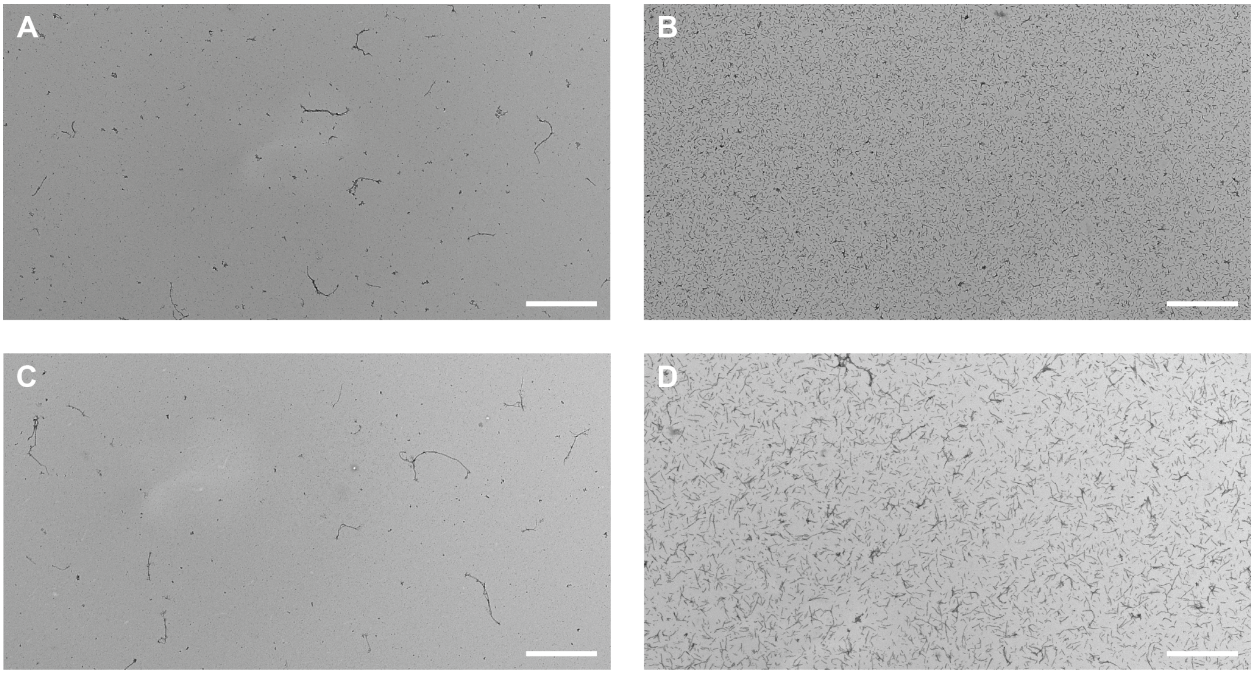

Supplementary Figure 14: Demonstration of increased amplification in the presence of cut slats. TEM images of **A** growth without cut slats and **B** growth + scission of v5 design with 6-segment extensions and u-filler (as in Supplementary Figure 1B). **C** growth without cut slats and **D** growth + scission of v5 design with 4-segment extensions and u-filler (growth-slats as in Supplementary Figure 1C and u-filler as in Supplementary Figure 1B). Scale bars are 1 µm. Reactions were performed at 20 mM Mg^{2+}, 5 mM Tris, 1 mM EDTA, pH 8.0, 0.01% Tween-20, 85 °C for 5 minutes then 48 °C overnight for ~21 hours using 0.1 µM/nuc-slat, 0.2 µM per growth- and u-fill slat, 1 µM/cut-slat and 1 nM p8064. In order to prevent p8064 aggregation and get distinct ribbons in imaging, p8064 was added directly to the reactions on the thermocycler block at the beginning of the 48 °C incubation instead of along with all the other reactants before the 85 °C denaturation step (as in the rest of the experiments in this work). Experiments in Figures 2 and 3 used 65 ºC denaturation to avoid aggregation issues (see Supplementary Figure 24 for a comparison). Note that ribbons without cut slats do not have a passivating polyT brush, meaning they are likely to stick together or aggregate.

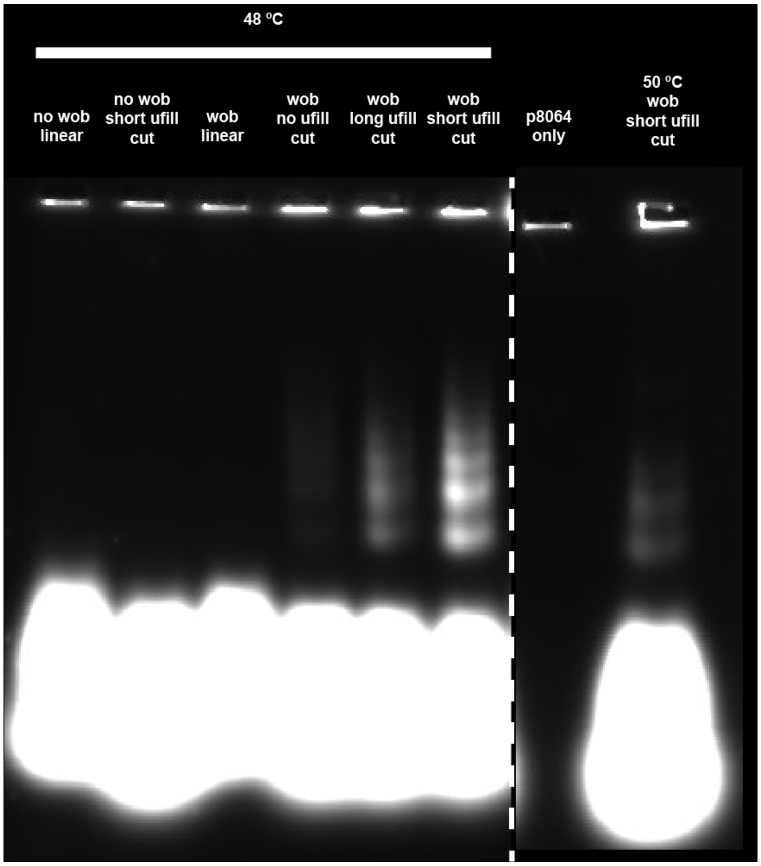

Supplementary Figure 15: Effect of the presence of wobbles and u-filler on v5 3CR amplification. “Short” corresponds to the 5-segment u-filler depicted in Supplementary Figure 1. The “long” u-filler is extended vertically by two helices to bind an additional two binding sites on two of the x-slat extensions for a total of 9-segment binding. Given that the “long” u-filler resulted in less amplification and is expected to generate more spurious nucleation than the shorter version, it was not used in any other experiment shown. 48 °C was chosen as the default temperature based on this experiment and Supplementary Figure 23. Reactions were performed at 20 mM Mg^{2+}, 5 mM Tris, 1 mM EDTA, pH 8.0, 0.01% Tween-20, 85 °C for 5 minutes then 48/50 °C overnight for ~20 hours using 0.25 μM per growth-, nuc-, and u-fill slat, 1 μM/cut-slat, and 1 nM p8064 target.

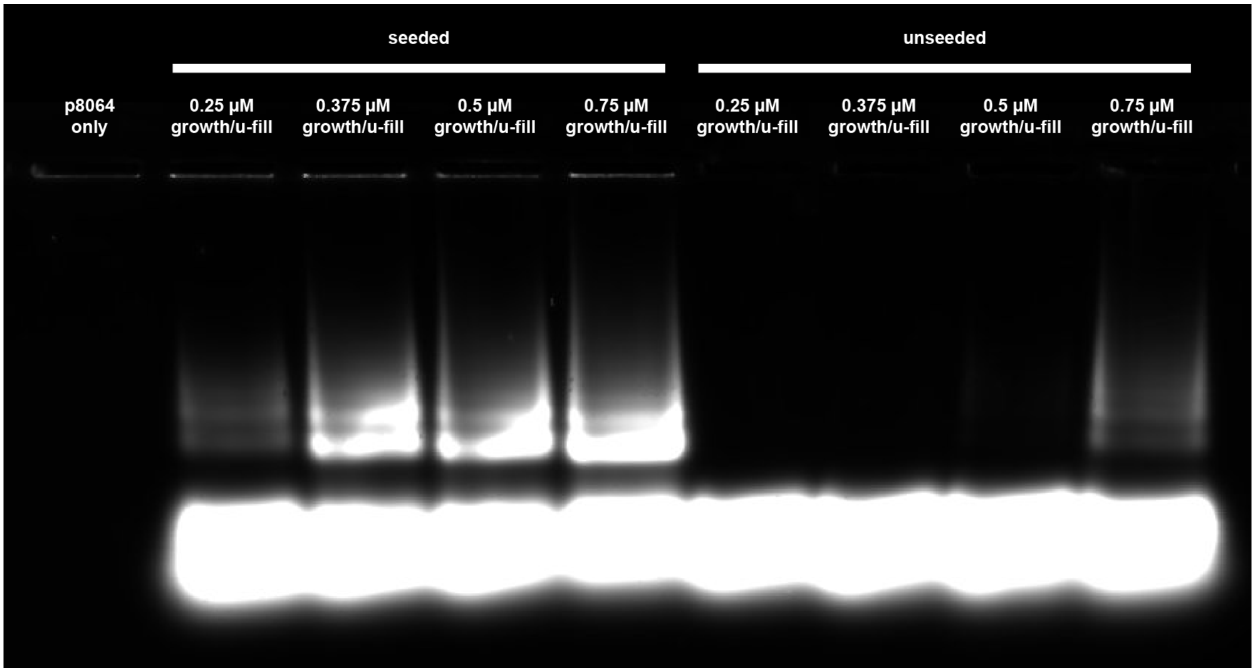

Supplementary Figure 16: Effect of growth- and u-filler slat concentration on assembly rate and spurious nucleation of v5 3CR (Supplementary Figure 1B), showing both greater amplification and spurious nucleation at higher slat concentrations. Reactions were performed at 20 mM Mg^{2+}, 5 mM Tris, 1 mM EDTA, pH 8.0, 0.01% Tween-20, 85 °C for 5 minutes then 48 °C overnight for ~20 hours using 0.25 µM/nuc-slat, 1 µM/cut-slat, and 1 nM p8064 target.

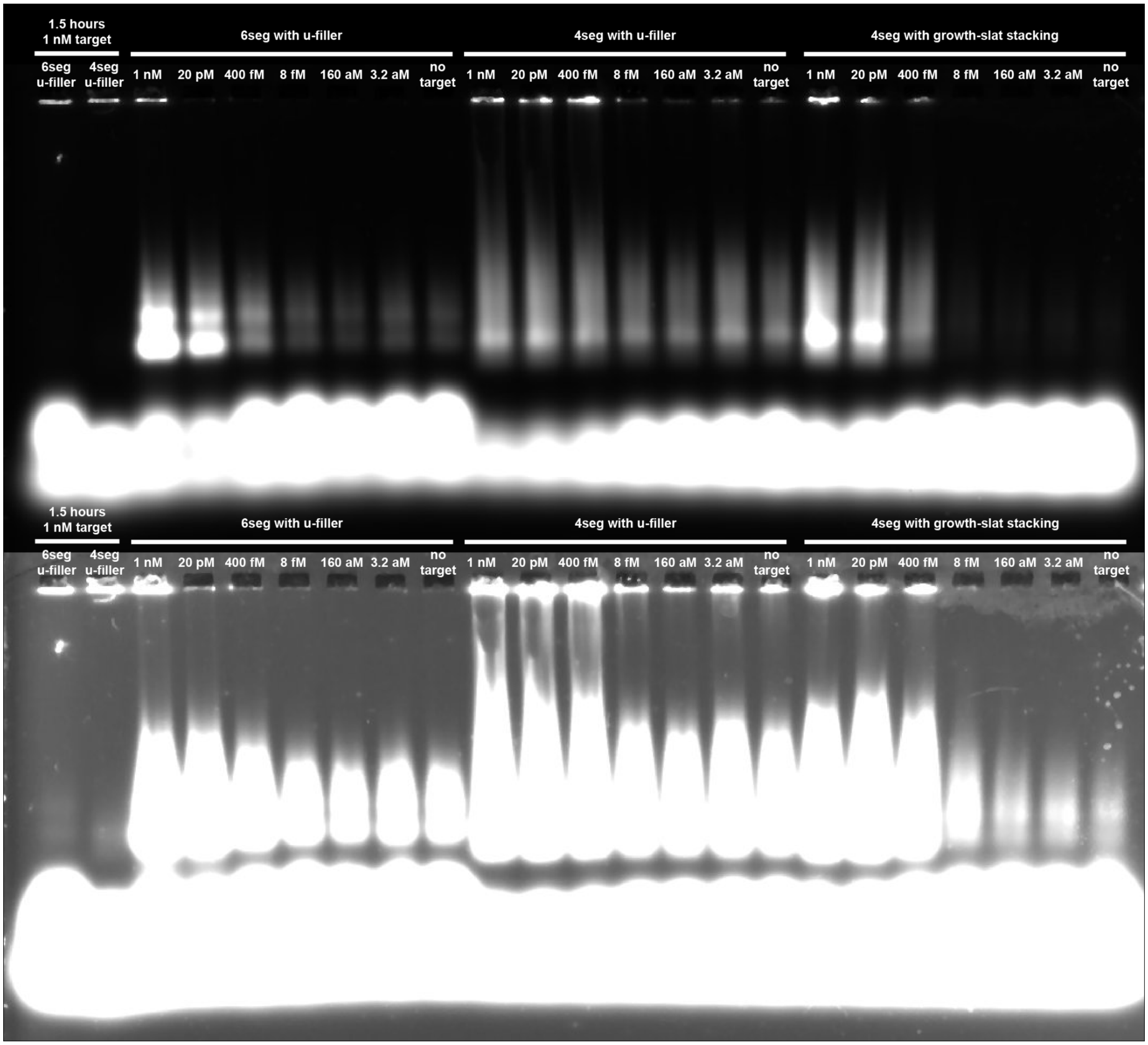

Supplementary Figure 17: Left: shorter incubations (1.5 hours) for v5 3CR produce only a very faint signal, hence overnight incubations were used throughout this work. Remainder of gel: overnight assembly. When setting up this experiment, these reactions were at room temperature for longer than most other experiments presented, likely explaining the greater amount of spurious nucleation and suggesting that 85 °C for 5 minutes is insufficient to destroy all spurious nuclei. However, we can see that the growth-slat stacking design (Supplementary Figure 1C) has lower rates of spurious nucleation than the designs using u-fillers. Top and bottom are images of the same gel at different contrasts. Reactions were performed at 20 mM Mg^{2+}, 5 mM Tris, 1 mM EDTA, pH 8.0, 0.01% Tween-20, 85 °C for 5 minutes then 48 °C overnight for ~21 hours using 0.1 μM/nuc-slat, 0.2 μM per growth- and u-fill slat, 1 μM/cut-slat and variable p8064 target concentrations.

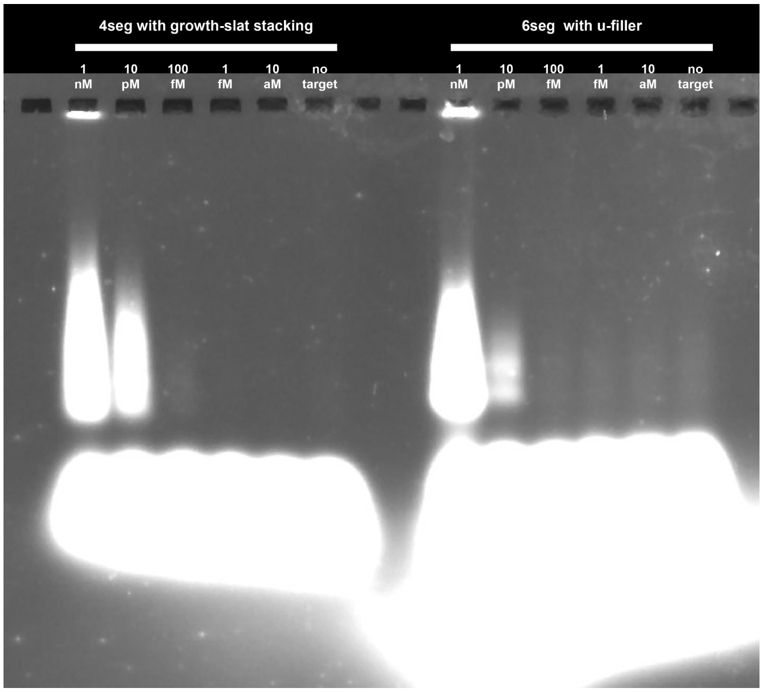

Supplementary Figure 18: Limit of detection of growth-slat stacking design (Supplementary Figure 1C) and 6seg u-filler design (Supplementary Figure 1B) after 19 hour assembly with 0.15 μM/slat is < 10 pM. Note two nights' assembly under these conditions without seed yielded significant spurious nucleation (not shown). Reactions were performed at 20 mM Mg^{2+}, 5 mM Tris, 1 mM EDTA, pH 8.0, 0.01% Tween-20, 85 °C for 5 minutes then 48 °C overnight for ~19 hours using 0.15 μM per growth-, nuc- and u-fill slat, 1 μM/cut-slat and variable p8064 target concentrations.

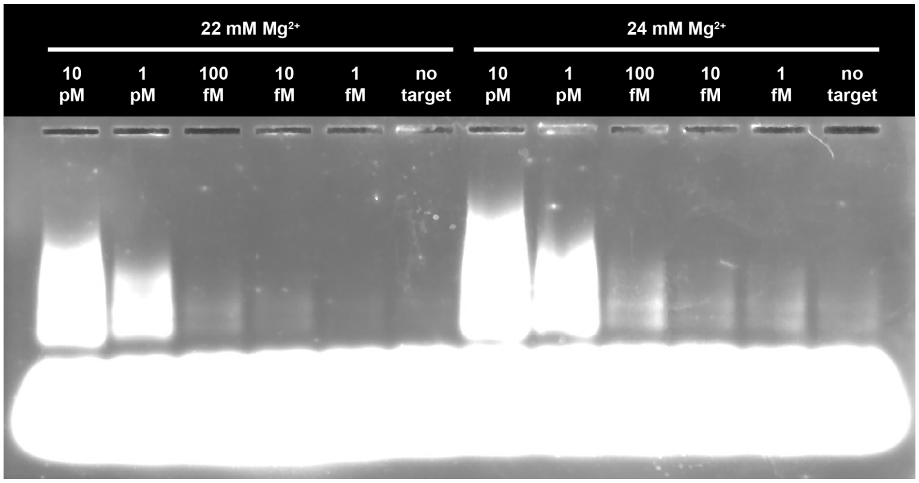

Supplementary Figure 19: Limit of detection of growth-slat stacking design (Supplementary Figure 1C) after 17 hour assembly with 0.15 μM/slat and higher magnesium concentrations is < 1 pM. Reactions were performed at 22–24 mM Mg^{2+}, 5 mM Tris, 1 mM EDTA, pH 8.0, 0.01% Tween-20, 85 °C for 10 minutes then 48 °C overnight for ~17 hours using 0.15 μM per growth-, nuc- and u-fill slat, 1 μM/cut-slat and variable p8064 target concentrations.

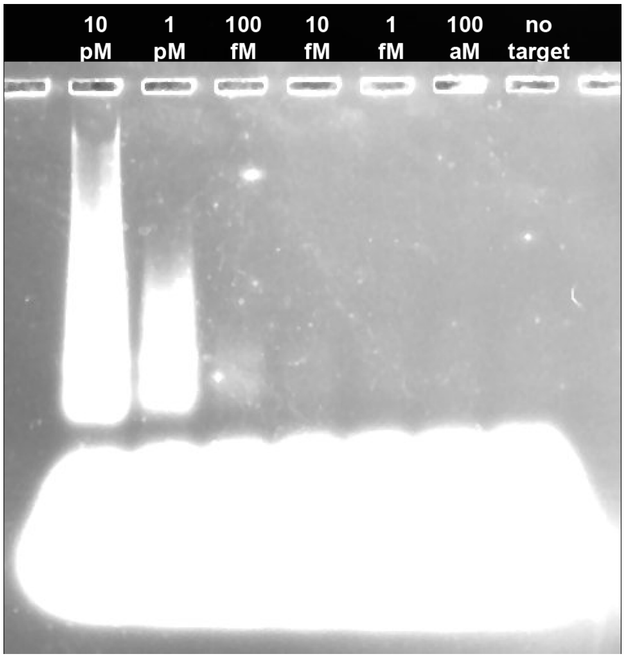

Supplementary Figure 20: Limit of detection of growth-slat stacking design (Supplementary Figure 1C) after 39 hour assembly with 0.1 μM/slat is < 1 pM. Reactions were performed at 20 mM Mg^{2+}, 5 mM Tris, 1 mM EDTA, pH 8.0, 0.01% Tween-20, 85 °C for 10 minutes then 48 °C overnight for ~39 hours using 0.1 μM per growth- and nuc- slats, 1 μM/cut-slat and variable p8064 target concentrations.

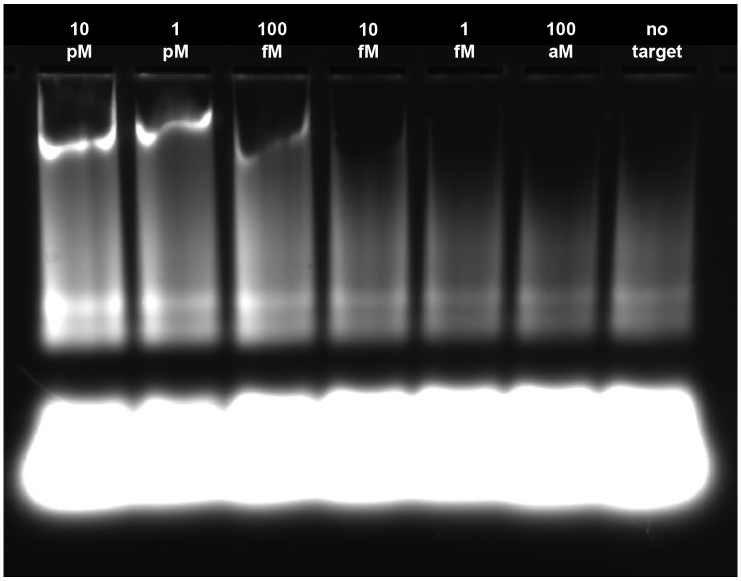

Supplementary Figure 21: Limit of detection of growth-slat stacking design (Supplementary Figure 1C) after 66 hour assembly with 0.1 μM/slat is < 1 pM with more significant spurious nucleation visible than after two nights under the same conditions as in Supplementary Figure 13. Reactions were performed at 20 mM Mg^{2+}, 5 mM Tris, 1 mM EDTA, pH 8.0, 0.01% Tween-20, 85 °C for 10 minutes then 48 °C overnight for ~66 hours using 0.1 μM per growth- and nuc- slats, 1 μM/cut-slat and variable p8064 target concentrations.

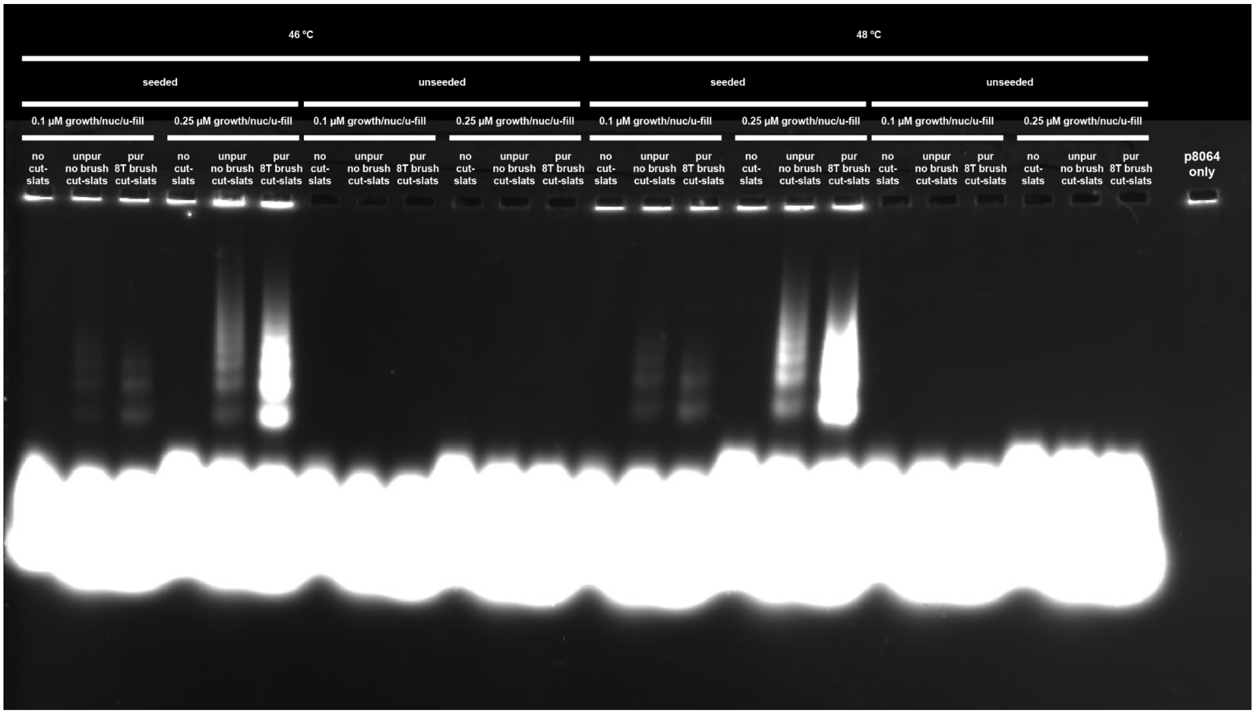

Supplementary Figure 22: Effect of slat concentration and temperature on v5 3CR, as well as PAGE purification and inclusion of an 8T-brush on cut-slats. While scission is detected with the unpurified cut-slats (with significantly more amplification at higher slat concentrations), the PAGE-purified slats with a T brush are significantly more effective, without detectable spurious nucleation, at both temperatures tested. Unless otherwise noted, PAGE-purified cut-slats were used throughout this work. Reactions were performed at 20 mM Mg^{2+}, 5 mM Tris, 1 mM EDTA, pH 8.0, 0.01% Tween-20, 85 °C for 5 minutes then 46/48 °C overnight for ~20 hours using 0.1/0.25 µM per growth-, nuc-, and u-fill slat, 1 µM/cut-slat, and 1 nM p8064 target.

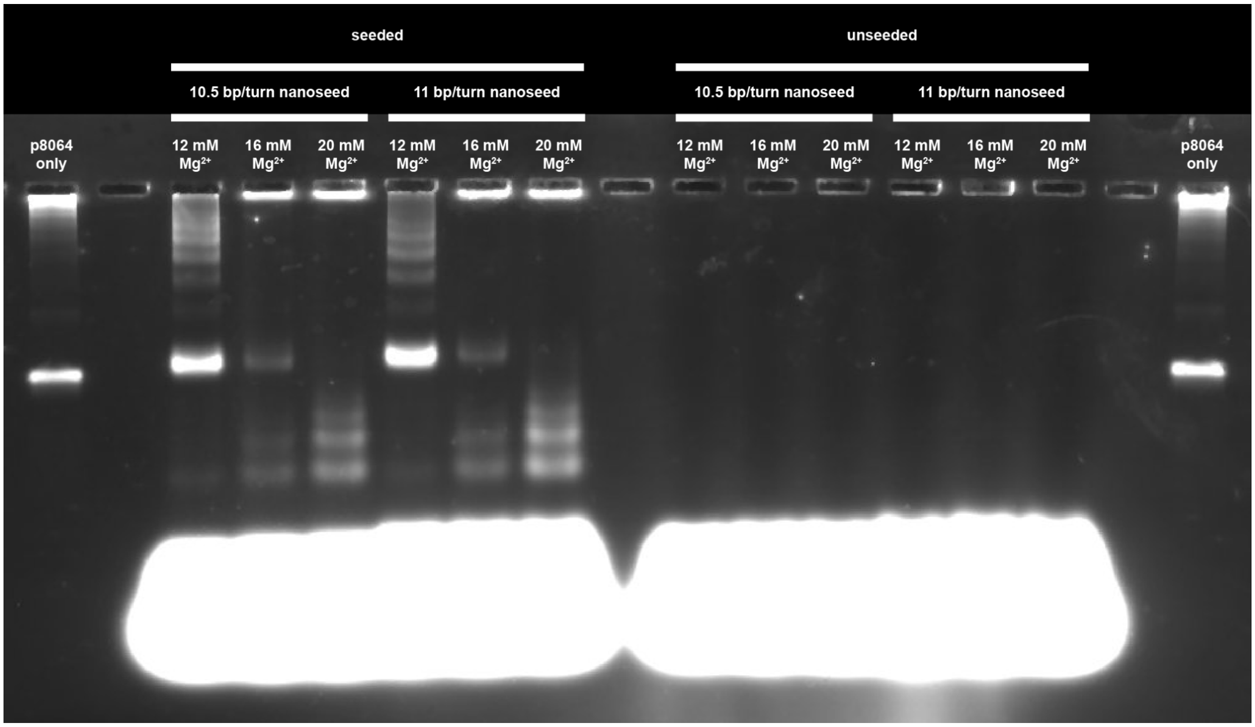

Supplementary Figure 23: Selection of nanoseed design (10.5 bp/turn for minimal twisting, c.f. 11 bp/turn for potentially faster assembly). v5 3CR (i.e. growth + scission) was seeded from either 10.5 bp/turn or 11 bp/turn nanoseeds detecting p8064 scaffold at different magnesium concentrations. Both designs gave rise to amplification, with greater degrees of scission seen at higher magnesium concentrations. The 11 bp/turn nanoseed was chosen for all experiments unless otherwise noted. Reactions were performed at 12-20 mM Mg^{2+}, 5 mM Tris, 1 mM EDTA, pH 8.0, 0.01% Tween-20, 85 °C for 5 minutes then 46 °C overnight for ~21 hours using 0.1 µM per growth-, nuc-, and u-fill slat, 1 µM/cut-slat, and 1 nM p8064 target. The p8064 only condition was incubated with 12 mM Mg^{2+}, hence the aggregation seen here is lower than subsequent experiments where 20 mM Mg^{2+} was used.

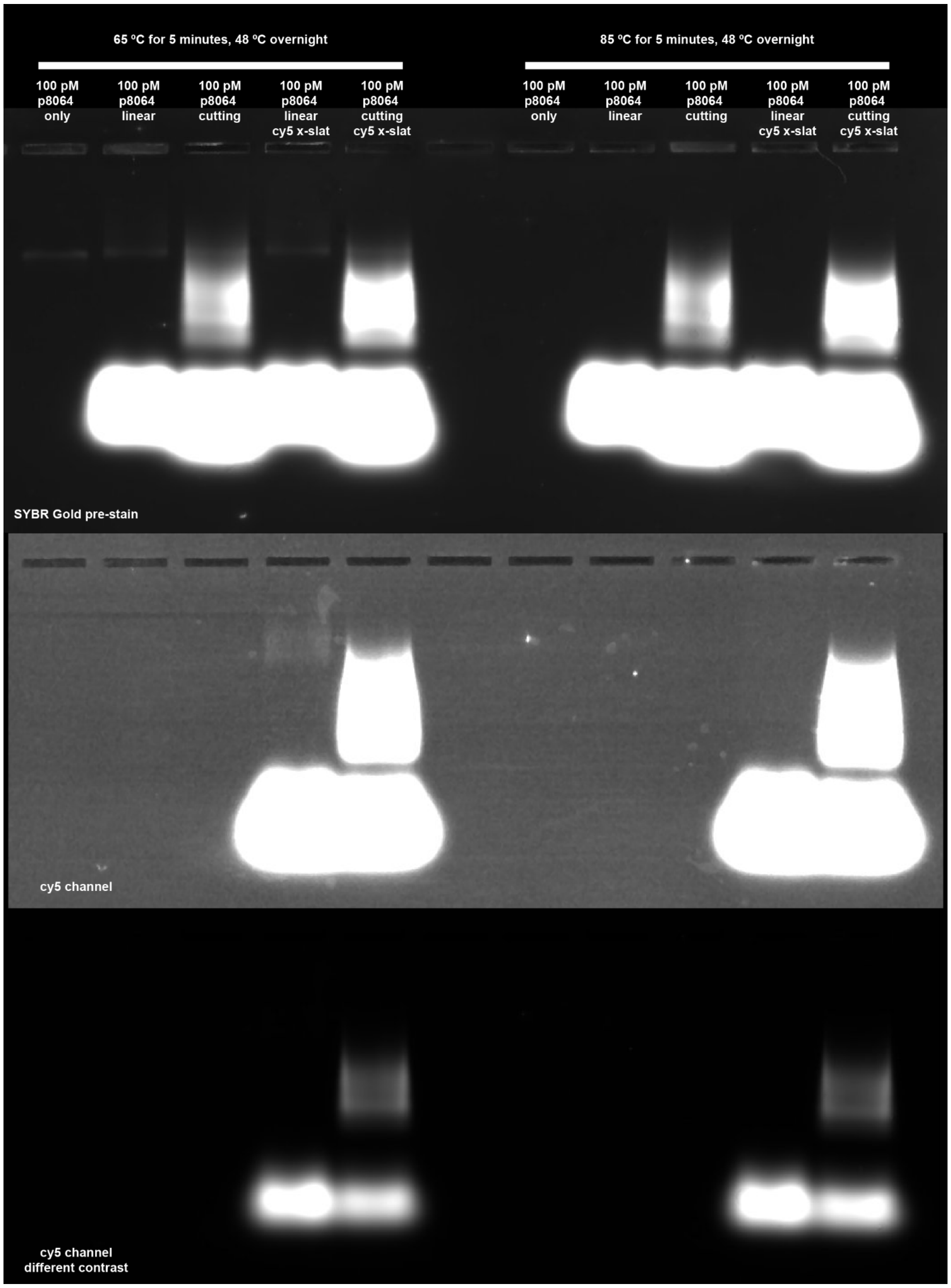

Supplementary Figure 24: Comparison of linear and exponential amplification for design from Supplementary Figure 1C with and without cy5-fluorophore 3’ labeling of the top x-slat (also used in Figure 2D and Figure 3). Denaturation at 85 °C induced aggregation of 100 pM p8064 at these experimental conditions while denaturation at 65 °C did not (however using 1 nM p8064 as in Figure 3C still showed significant aggregation). ImageJ quantification of cy5 intensity of linear vs cutting conditions corresponds to a ~233x increase in signal intensity, corresponding to ~8 doublings (i.e. 1 doubling every 2 hours). Based on the ribbon growth-rate for these reactions (~200 nm linear ribbons), this corresponds to the growth-rate being rate-limiting (see stochastic simulations for further discussion). Reactions were performed at 20 mM Mg^{2+}, 5 mM Tris, 1 mM EDTA, pH 8.0, 0.01% Tween-20, 65/85 °C for 5 minutes then 48 °C overnight for ~19 hours using 0.15 μM per growth and nuc-slat, 1 μM/cut-slat and 100 pM p8064 target.

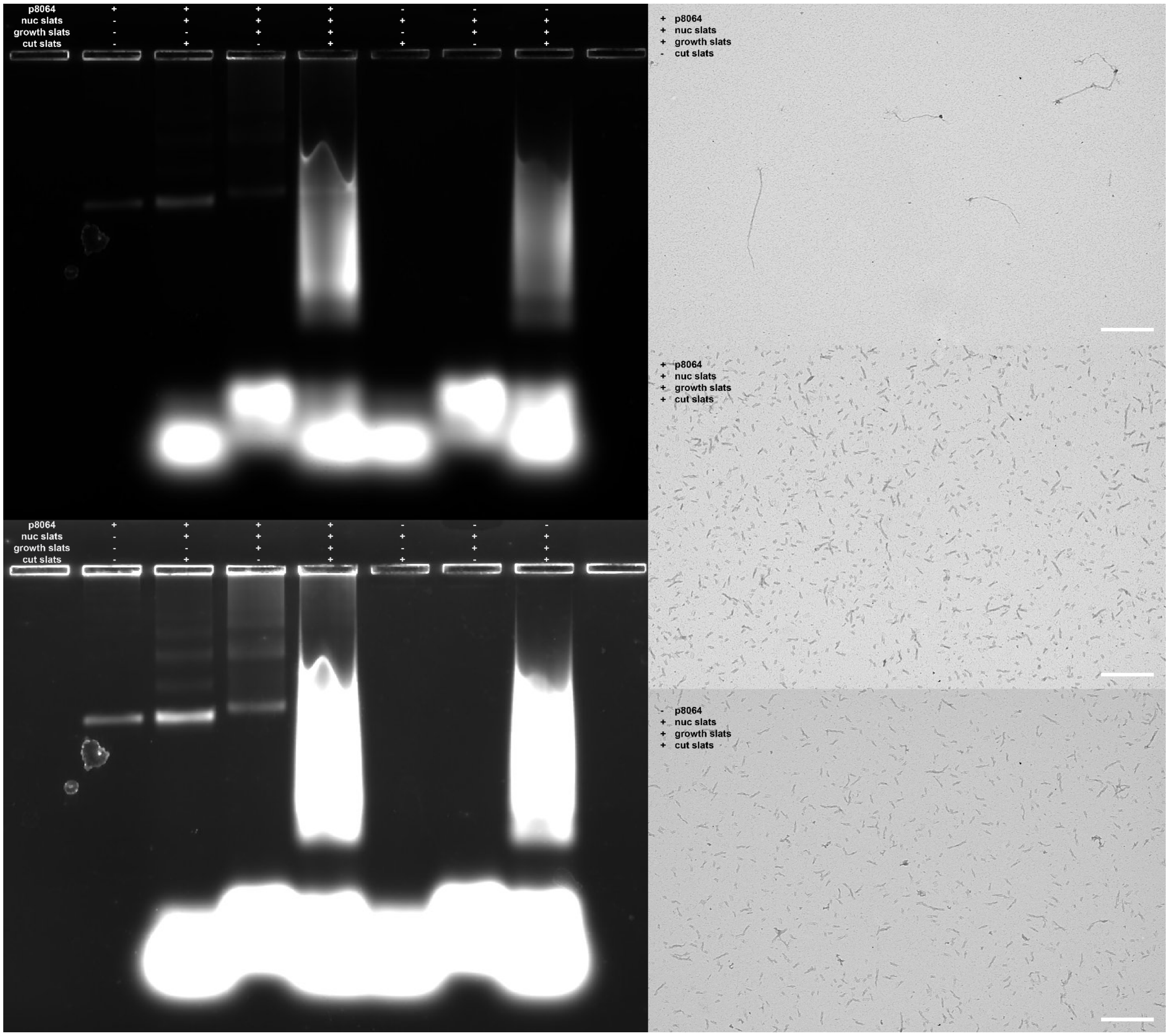

Supplementary Figure 25: 3CR detection with/without different reaction components using the design from Supplementary Figure 1C, with gel characterization (left) and TEM imaging (right). Reactions were performed at 20 mM Mg^{2+}, 5 mM Tris, 1 mM EDTA, pH 8.0, 0.01% Tween-20, 65 °C for 5 minutes then 48 °C overnight for ~20.5 hours using 0.15 µM/growth-slat, 0.1 µM/nuc-slat, 1 µM/cut-slat and 1 nM p8064. Scale bars are 400 nm. We note that greater spurious nucleation was detected in this experiment relative to other ones at similar reaction conditions (e.g. Figure 2D and Figure 3C). We believe that this is due to the longer-term storage of the PAGE-purified slats in this experiment (~7 months), as we have found that older slat stocks can be more prone to such behavior.

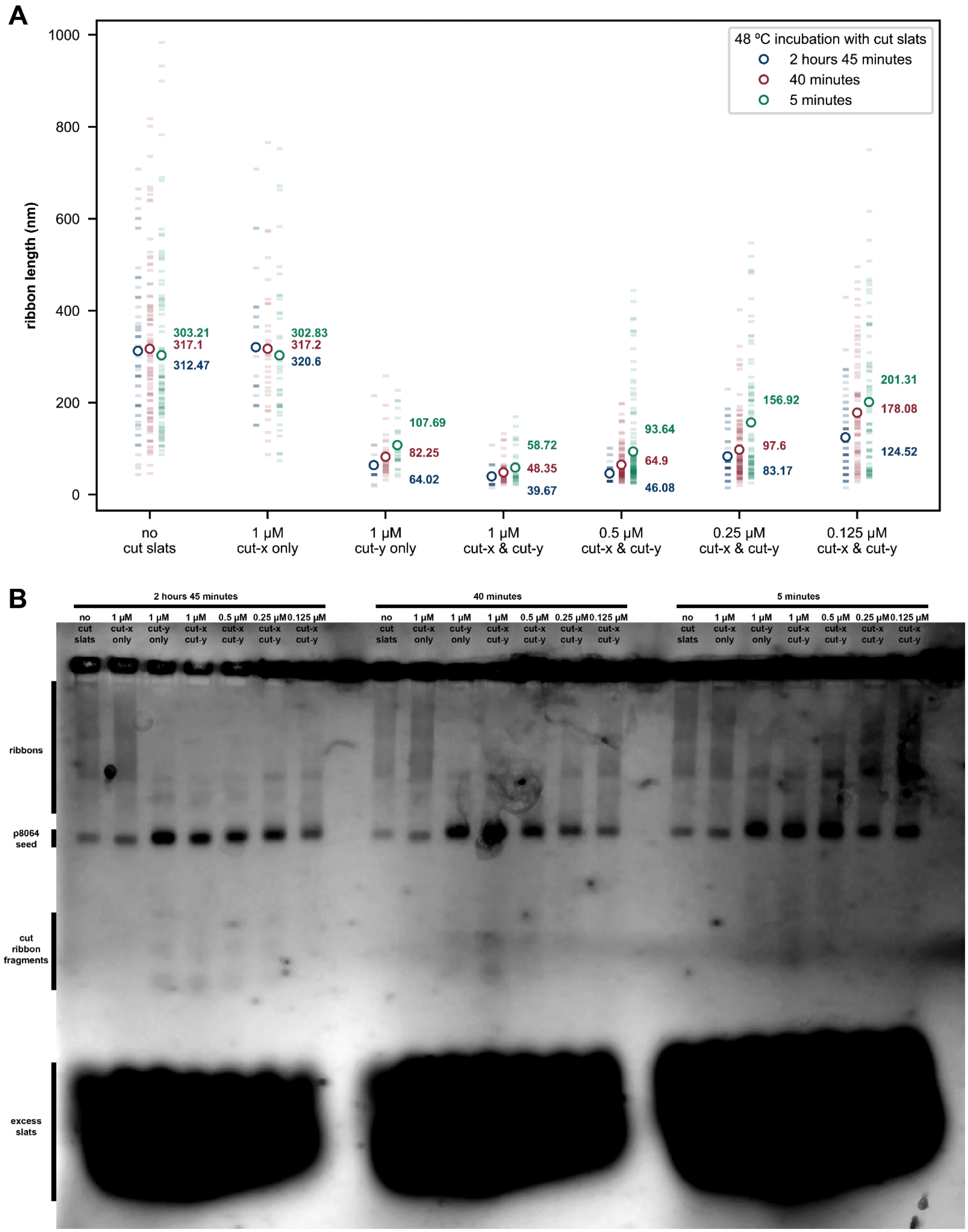

Supplementary Figure 26: Dilution of no-cut-slat seeded reactions from Supplementary Figure 25 into reactions containing variable concentrations of cut slats for different lengths of time to elucidate scission kinetics. Specifically, the linear ribbons were diluted 1:20 (i.e. an absolute

concentration of 50 pM p8064, 7.5 nM growth slats, 5 nM nuc slats) into the same reaction buffer (20 mM Mg^{2+}, 5 mM Tris, 1 mM EDTA, pH 8.0, 0.01% Tween-20) with cut slats added, and then placed back into the thermocycler at 48 ºC. **A**, Quantification of ribbon lengths from TEM images, measured using the FIJI segmented line tool, with an average of ~60 measurements per condition. Circles and annotations represent the mean length for each condition, and transparent rectangles each individual data point. **B**, Agarose gel of the samples from **A**. Due to potential error of length measurement of the shortest ribbon fragments and potential impact of the reactions sitting at room temperature for a few minutes while setting up the gel and staining the TEM grids, we suggest interpreting this data qualitatively and not quantitatively. As expected, longer incubations and higher cut-slat concentrations resulted in greater degrees of scission. It is notable that even for the shortest incubation and lowest slat concentrations tested (5 minutes and 0.125 μM), significant scission was observed, with the mean length being ~⅔ of the no-cut-slat control. We further note the presence of scission with 1 μM cut-y only (at a rate comparable to 0.25-0.5 μM of both cut-x and cut-y), but not 1 μM cut-x only (see Supplementary Figure 28 for discussion).

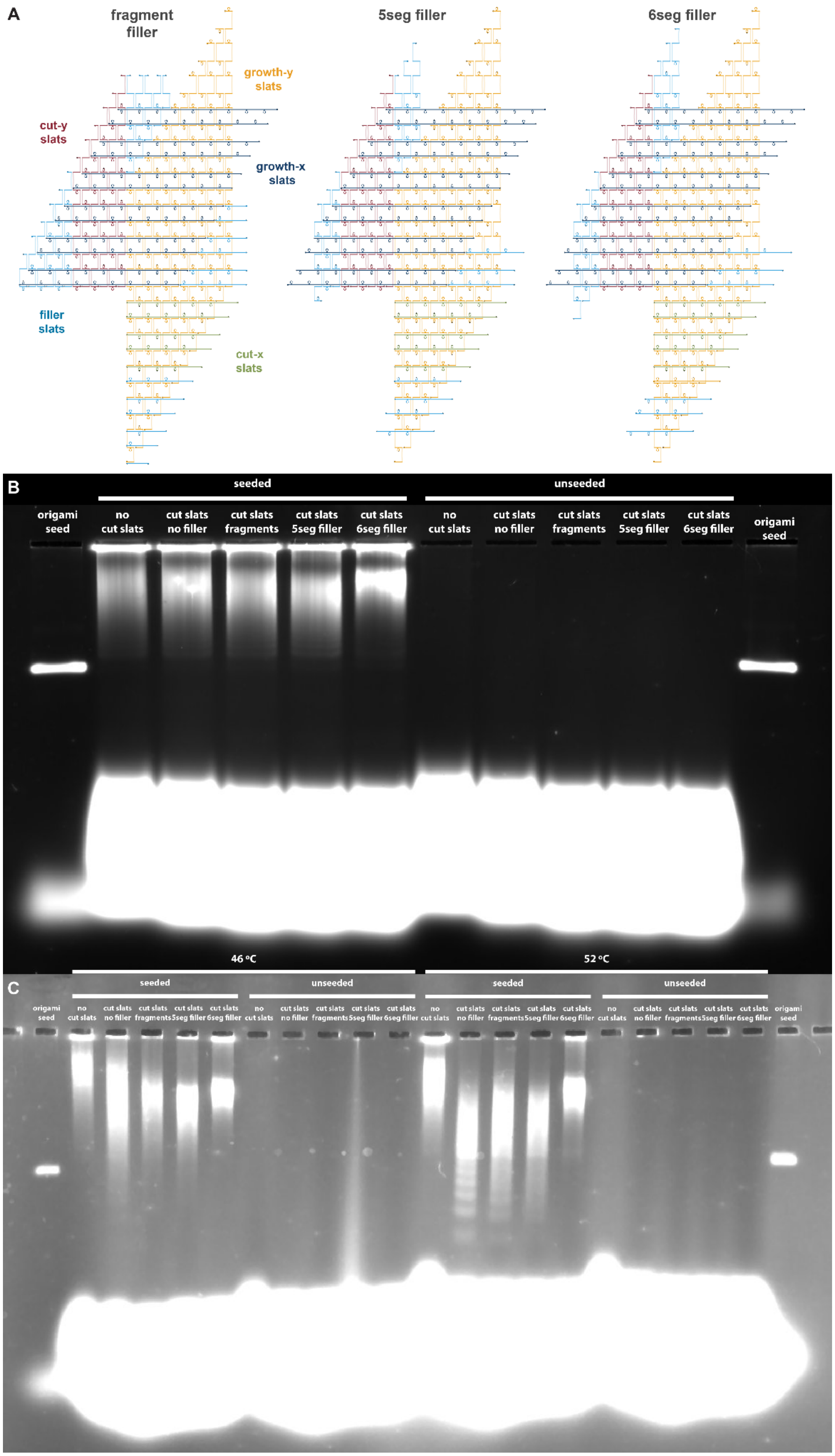

Supplementary Figure 27: 3CR detection with different reaction components using the v6 design from Supplementary Figure 1A. **A**, scadnano designs of Supplementary Figure 1A with different linear “filler” slats bound to the parts of extensions that do not have cut slats bound. Filler and cut slats all have an 8T brush. **B**, Assemblies after overnight growth showing significant linear growth, but no obvious scission (no bands appearing that migrate faster than the seed). Reactions were performed at 12 mM Mg^{2+}, 5 mM Tris, 1 mM EDTA, pH 8.0, 0.01% Tween-20, 85 °C for 5 minutes then 46 °C overnight using 0.1667 µM/growth-slat, 0.25 µM/nuc-slat, 0.25 µM/cut-slat and filler-slat and 1 nM DNA-origami seed[1]. **C**, Reactions from **B** were diluted 1:10 in reaction buffer with 16 mM Mg^{2+} and 1 µM of cut- and filler- slats and incubated for another night. Stronger scission is observed in conditions where the extensions are less occupied by filler slats.

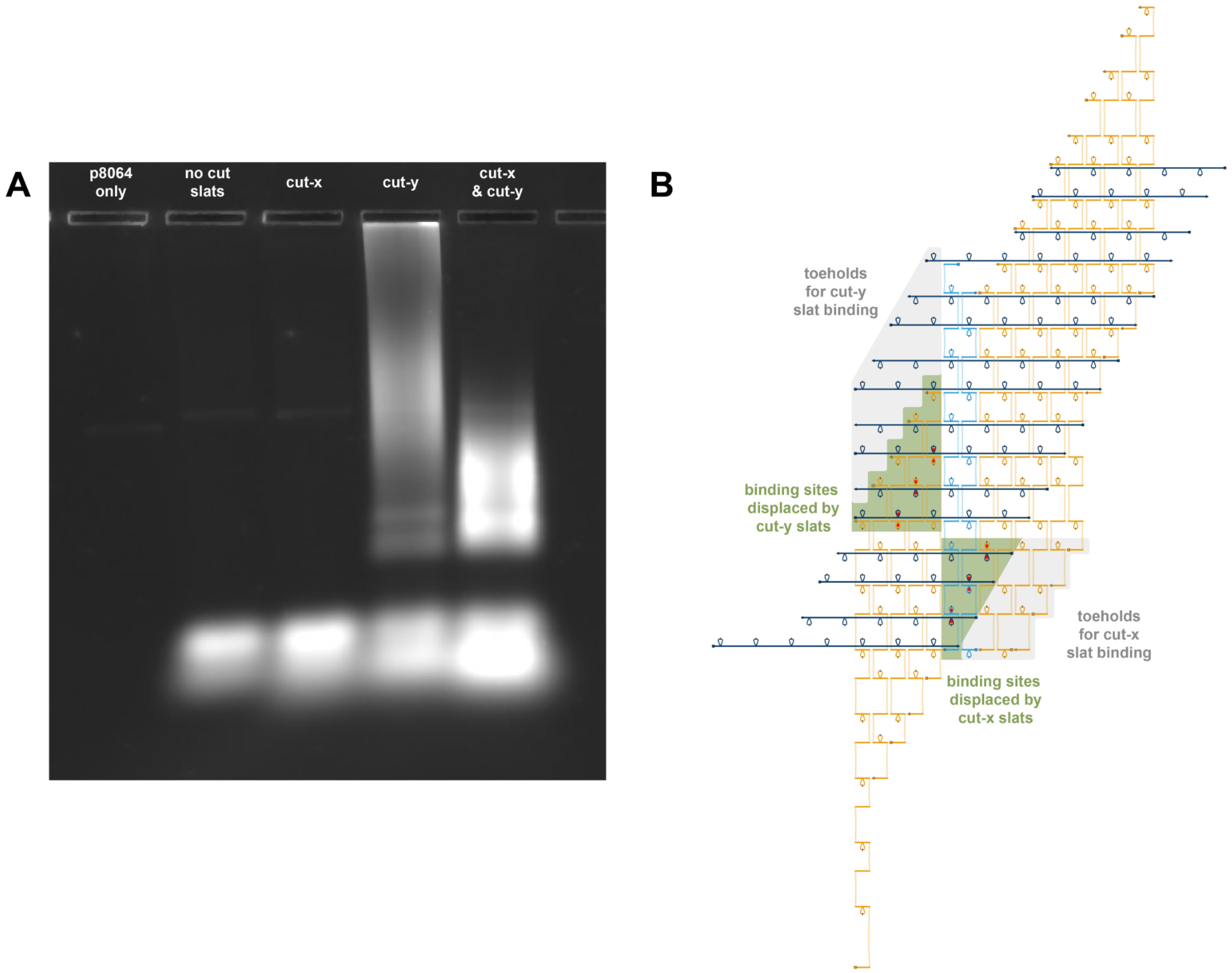

Supplementary Figure 28: A, 3CR detection with/without different cut slats using the design from Supplementary Figure 1C. As in Supplementary Figure 26, there is significant scission with cut-y only but not cut-x only, albeit at a slower rate than both cut-x and cut-y. Reactions were performed at 20 mM Mg^{2+}, 5 mM Tris, 1 mM EDTA, pH 8.0, 0.01% Tween-20, 65 °C for 5 minutes then 48 °C overnight for ~17.5 hours using 0.15 µM/growth-slat, 0.1 µM/nuc-slat, 1 µM/cut-slat and 100 pM p8064. **B**, Reproduction of Figure 1E (ribbon design prior to cut-slat binding), highlighting in green the binding sites designed to be displaced by cut slats. In particular, cut-y slats need to displace 15 binding sites in total, of which 3 have wobbles (in red), and out of the non-wobble binding sites, 5 have insertions (i.e. are 6 nt instead of 5 nt). In contrast, cut-x slats need to displace 10 binding sites in total, of which 3 have wobbles, and out of the non-wobble binding sites, 1 has an insertion. One possibility is that under these reaction conditions, the cut-x displacement triangle is sufficiently unstable (due to fewer/weaker binding sites), such that the steric crowding induced by cut-y displacement is enough to cause non-cut-slat-mediated unbinding of the growth-x slat segments and therefore scission. In contrast, for thicker v6 ribbons (such as the one in Supplementary Figure 29), cut-slat binding in both directions is necessary to displace all the growth-slat binding sites.

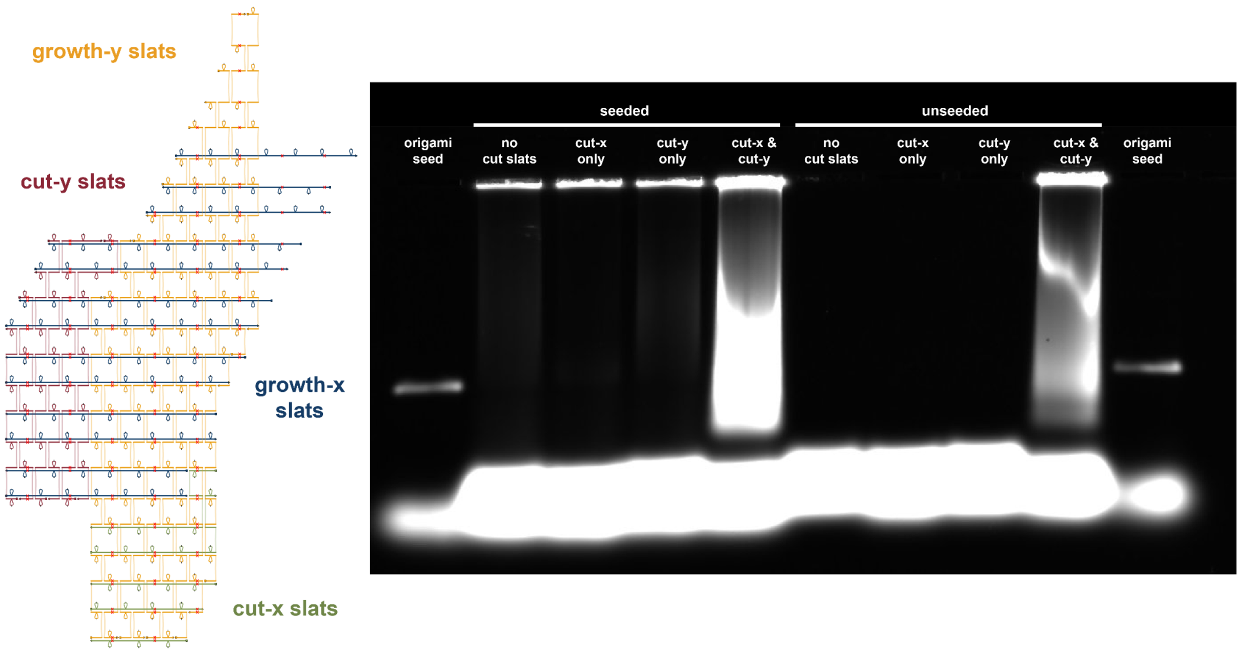

Supplementary Figure 29: scadnano design (left) and gel results following overnight growth (right) of an alternative v6 3CR design with cut slats that straddle multiple extensions and variable length growth slats. Significant additional amplification (and spurious nucleation) was observed upon addition of both cut-x and cut-y slats, with cut-x only or cut-y only looking more similar to the no cut slats condition. Reactions were performed at 14 mM Mg^{2+}, 5 mM Tris, 1 mM EDTA, pH 8.0, 0.01% Tween-20, 52 °C overnight using 0.5 μM/growth- and nuc-slat, 1 μM/cut-slat and 1 nM DNA-origami seed[1].

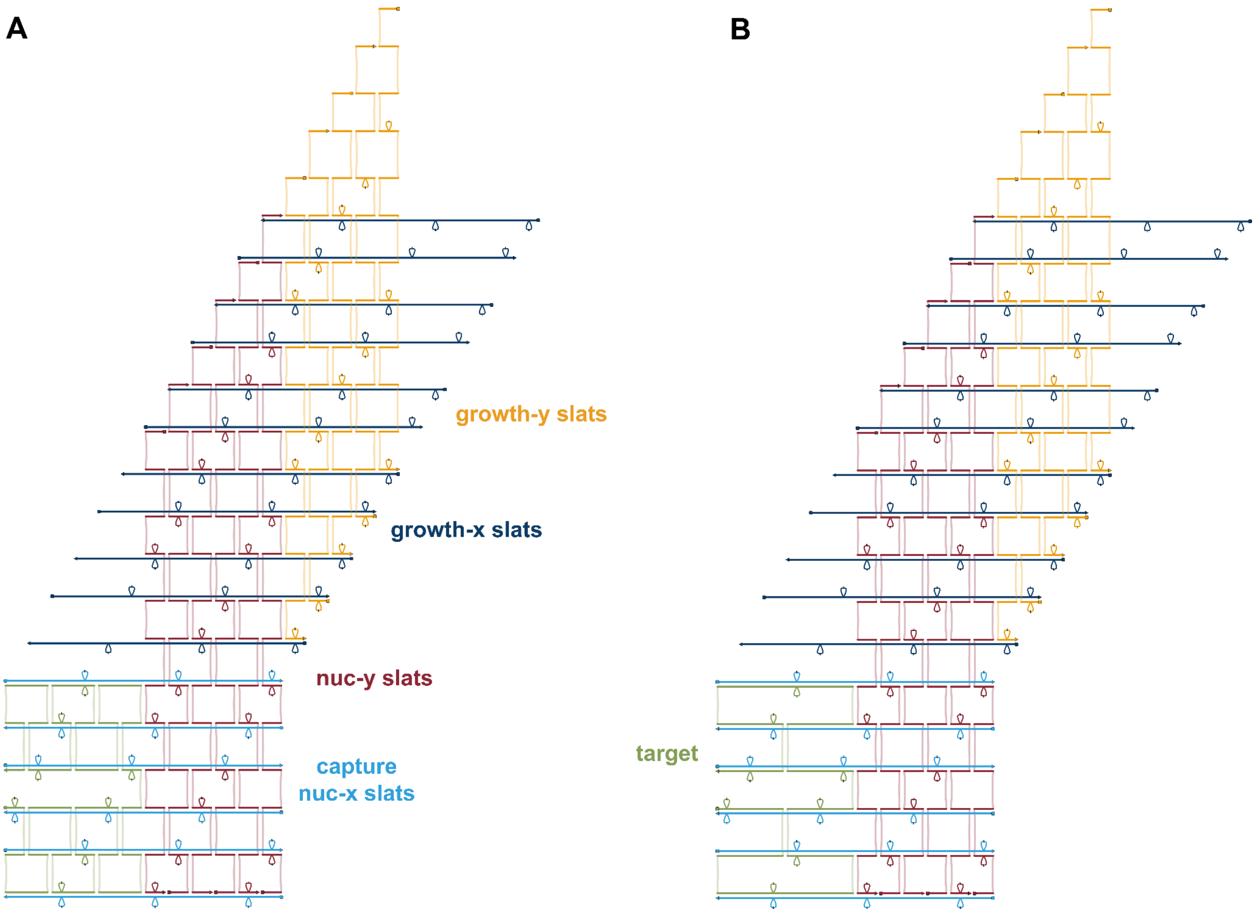

Supplementary Figure 30: scadnano designs of dense half-turn (**A**) and sparse 1.5-turn (**B**) crossover nanoseeds. 10.5 bp/turn detecting a 188 nt target and coupling with linear v6 growth-slats is shown, however the same architecture was used for both 11 bp/turn and 3CR assembly (detecting a 198 nt target sequence). Separate nuc-x and nuc-y slats were used to enable a plug-and-play approach of simply swapping the capture nuc-x slats to detect different targets (nuc-x slats are the same length so can be pool-purified, while each nuc-y slat was individually purified).

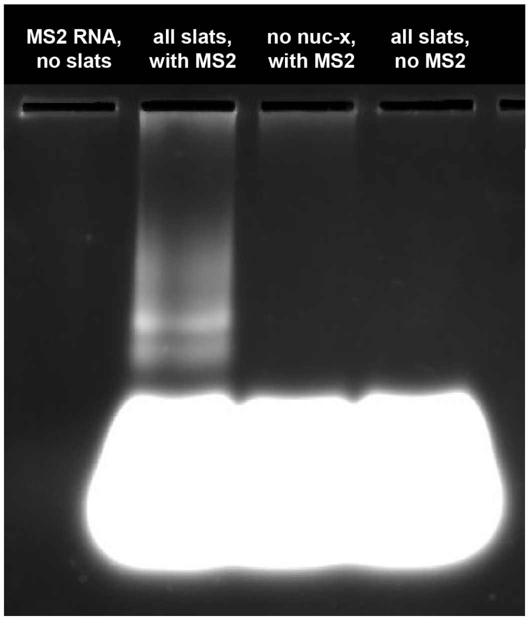

Supplementary Figure 31: 3CR detection of ~1 nM MS2 RNA using design from Supplementary Figure 1C. Reactions were performed at 20 mM Mg^{2+}, 5 mM Tris, 1 mM EDTA, pH 8.0, 0.01% Tween-20, 65 °C for 5 minutes then 48 °C overnight for ~17 hours using 0.15 μM per growth and nuc slats, 1 μM/cut-slat and ~1 nM MS2 RNA.

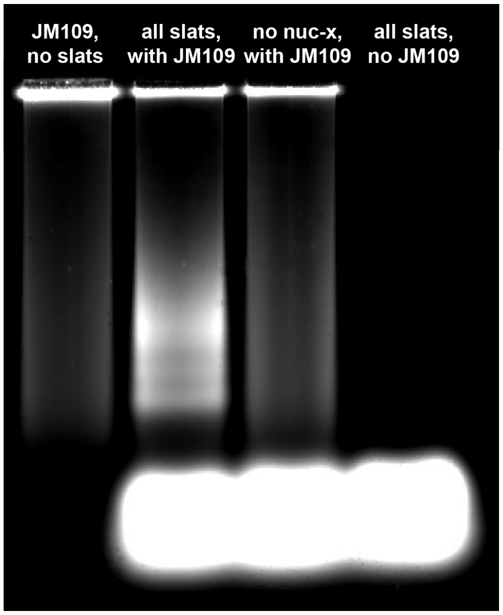

Supplementary Figure 32: 3CR detection of ~0.5 nM JM109 *E. coli* gDNA using design from Supplementary Figure 1C. Reactions were performed at 20 mM Mg^{2+}, 5 mM Tris, 1 mM EDTA, pH 8.0, 0.01% Tween-20, 85 °C for 10 minutes then 48 °C for ~20 hours using 0.15 μM per growth and nuc slats, 1 μM/cut-slat and ~0.5 nM purified JM109 gDNA with capture nuc-x slats against both forward and reverse strands of “gene 2” from Supplementary Figure 38.

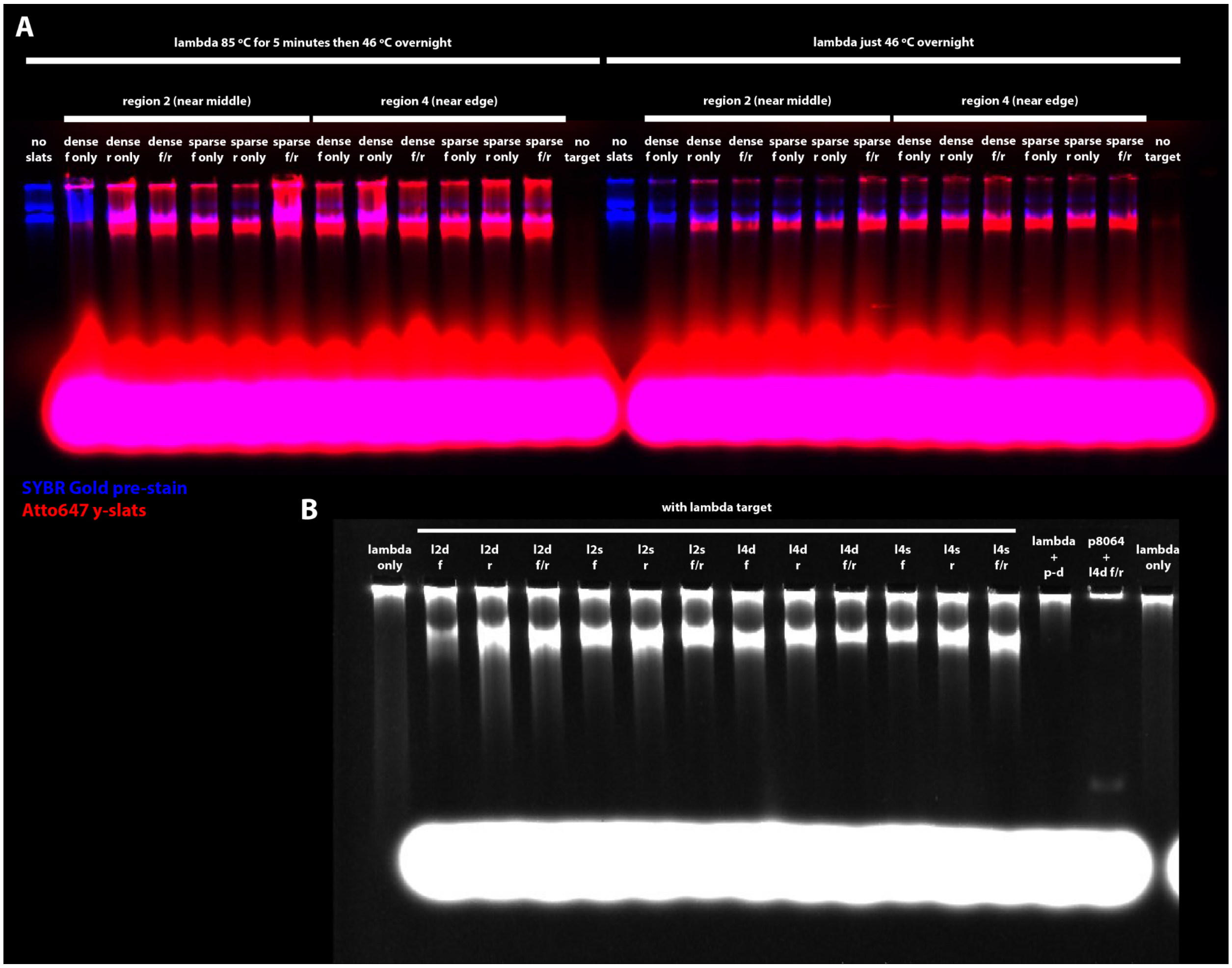

Supplementary Figure 33: A, Variability in dsDNA detection using v6.1 linear growth. Robust detection was observed upon capture of just the forward ("f") or reverse ("r") target strand for both sparse and dense nanoseeds, with stronger signal upon capture of both forward and reverse nanoseeds and more reliable detection of the target region closer to the edge of the lambda sequence. While less robust with more spurious nucleation, detection was possible even without the initial 85 °C denaturation step. Reactions were performed at 12 mM Mg^{2+}, 3.5 mM Tris, 0.7 mM EDTA, pH 8.0, 0.01% Tween-20, 85 °C for 5 minutes then 46 °C overnight using 0.5 μM/slat and ~0.8 nM lambda dsDNA target (New England Biolabs). **B**, Specificity of detection using v6.1 linear growth. The controls on the right show that in the presence of growth slats, p8064 capture slats (p-d), and lambda target there is no detectable ribbon formation. Likewise, in the presence of growth slats, lambda capture slats (l4d f/r, i.e. forward and reverse detection of lambda region 4 in a dense nanoseed), and p8064 target there is no detectable ribbon formation. Reactions were performed at 16 mM Mg^{2+}, 3.5 mM Tris, 0.7 mM EDTA, pH 8.0, 0.01% Tween-20, 85 °C for 5 minutes then 48 °C overnight using 0.5 μM/slat and ~1 nM lambda dsDNA target.

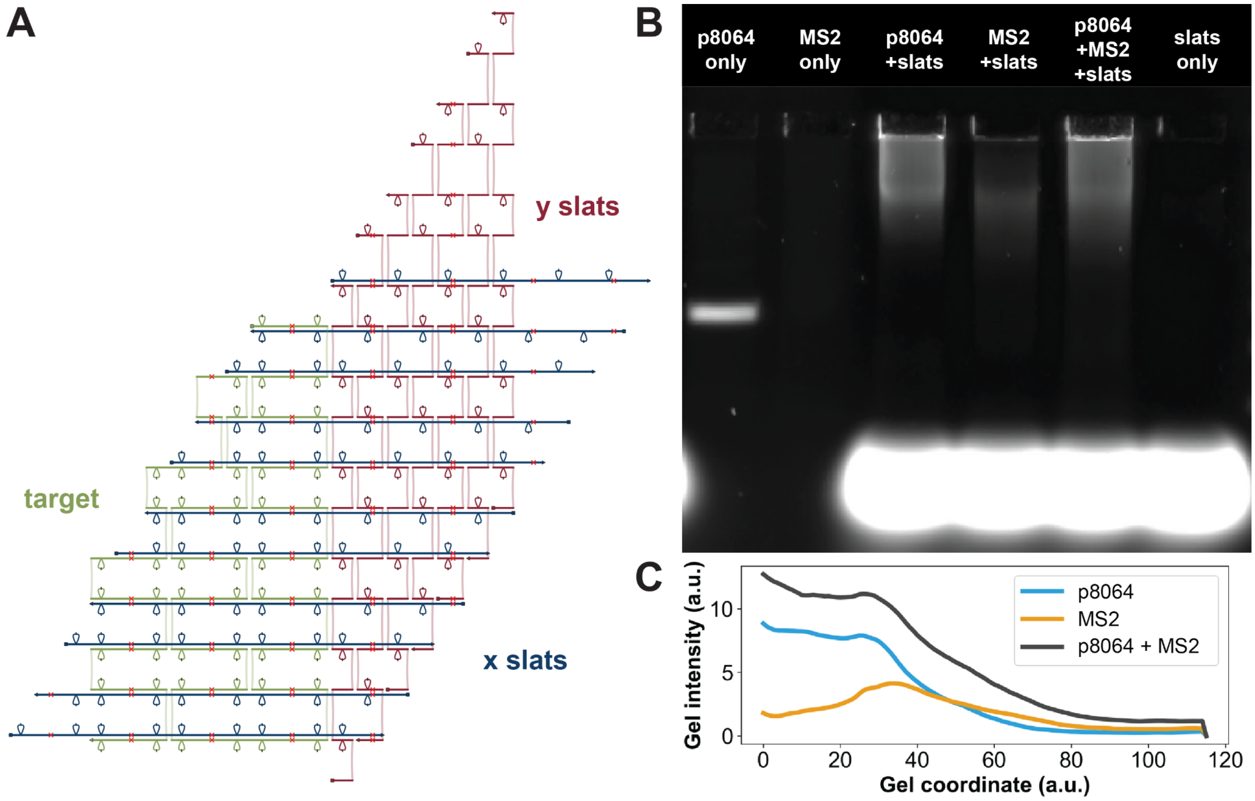

Supplementary Figure 34: Alternative nanoseed design and experimental conditions gives higher efficiency of RNA detection. **A**, scadnano design of nanoseed design for detecting a 408 nt single-stranded target. **B**, linear crisscross detection of 1 nM p8064 ssDNA, 1 nM MS2 ssRNA, or both 1 nM p8064 ssDNA and MS2 ssRNA in a one-pot reaction. Reactions were performed at 16 mM Mg2+, 10 mM MES, 1 mM EDTA, 0.01% Tween-20, pH 6.0, 65 °C for 5 minutes then 46 °C overnight using 0.5 µM per slat. **C**, gel densitometry of p8064 and MS2 detection, showing an additive signal for the detection of both targets in a one-pot reaction. The analysis was performed using the Python skimage.measure.profile_line function with linewidth set to approximately one third of the lane width centered at a homogeneous region of each gel band.

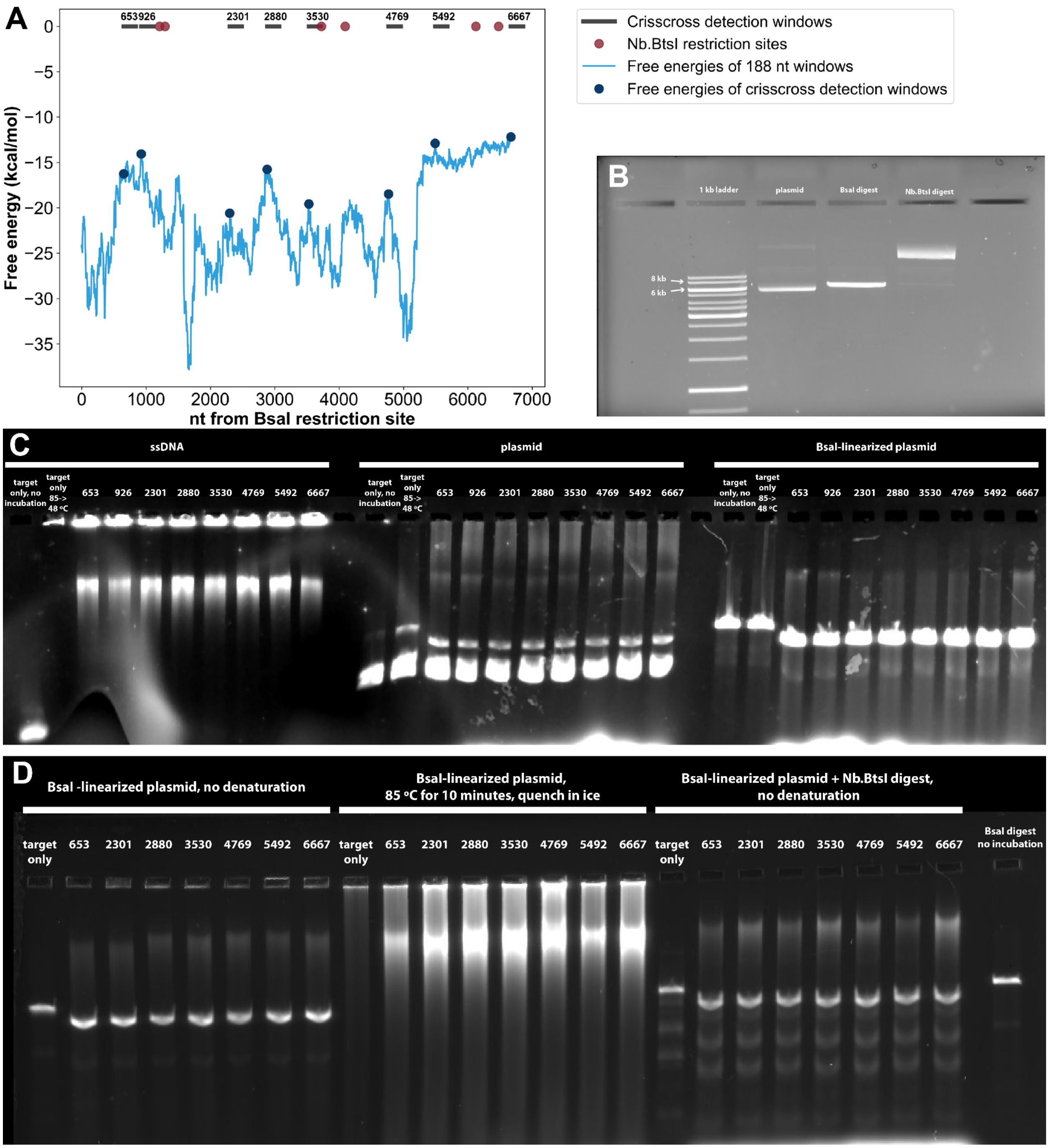

Supplementary Figure 35: Induction of kinetically trapped states increases dsDNA detection efficiency for a synthetic sequence (6851 bp plasmid with a single BsaI restriction site). Linear growth rather than exponential growth was assessed for this experiment. **A**, free energy of 188-nt regions of plasmid as calculated by NUPACK. Regions spaced out throughout the length of the plasmid with the lowest free energies of self-folding were chosen as crisscross detection windows. **B**, agarose gel validating expected plasmid length and linearization following BsaI digestion. The plasmid was prepared by inoculation of pET vector stock in 25 ml of sterile 2XYT media followed by shaking overnight at 200 rpm at 37 °C. Bacterial cells were centrifuged at 4200 rpm for 20 minutes, and the plasmid was isolated using a Qiagen mini-prep kit. Digestion

with BsaI-HFv2 (New England Biolabs) and Nb.BtsI was performed at 37 °C for 1 hour in rCutSmart buffer and purified using the New England Biolabs Monarch PCR and DNA clean-up kit. **C**, agarose gel comparing detection of ssDNA, dsDNA plasmid, and linearized dsDNA plasmid of the same target sequences. Reactions were performed at 85 °C for 5 minutes then 48 °C overnight using 12 mM Mg^{2+}, 3.5 mM Tris, 0.7 mM EDTA, pH 8.0, 0.01% Tween-20, using 0.5 μM/slat and 0.5 nM target. Detection uses a dense crossover nanoseed against both forward and reverse strands for dsDNA, and only forward for ssDNA. The ssDNA version of the plasmid sequence was produced by phagemid rescue with VCSM13 phage followed by ethanol precipitation. **D**, agarose gel demonstrating poor detection of dsDNA plasmid without heat-denaturation that is rescued by denaturation at 85 °C for 5 minutes followed by rapid cooling on ice. We can also see that the addition of nicks in the plasmid using Nb.BtsI does not sufficiently improve nanoseed formation. Reactions were performed at 12 mM Mg^{2+}, 3.5 mM Tris, 0.7 mM EDTA, pH 8.0, 0.01% Tween-20, using 0.5 μM/slat and 0.5 nM target. Reactions without the targets were denatured at 85 °C for 5 minutes prior to spiking in of the targets, followed by incubation at 48 °C overnight. "Target only" refers to reactions that only contain the target (e.g. plasmid) and buffer but no slats, and treated identically to the +slats conditions (e.g. heating to 85 ºC, quenching in ice, and then 48 ºC overnight).

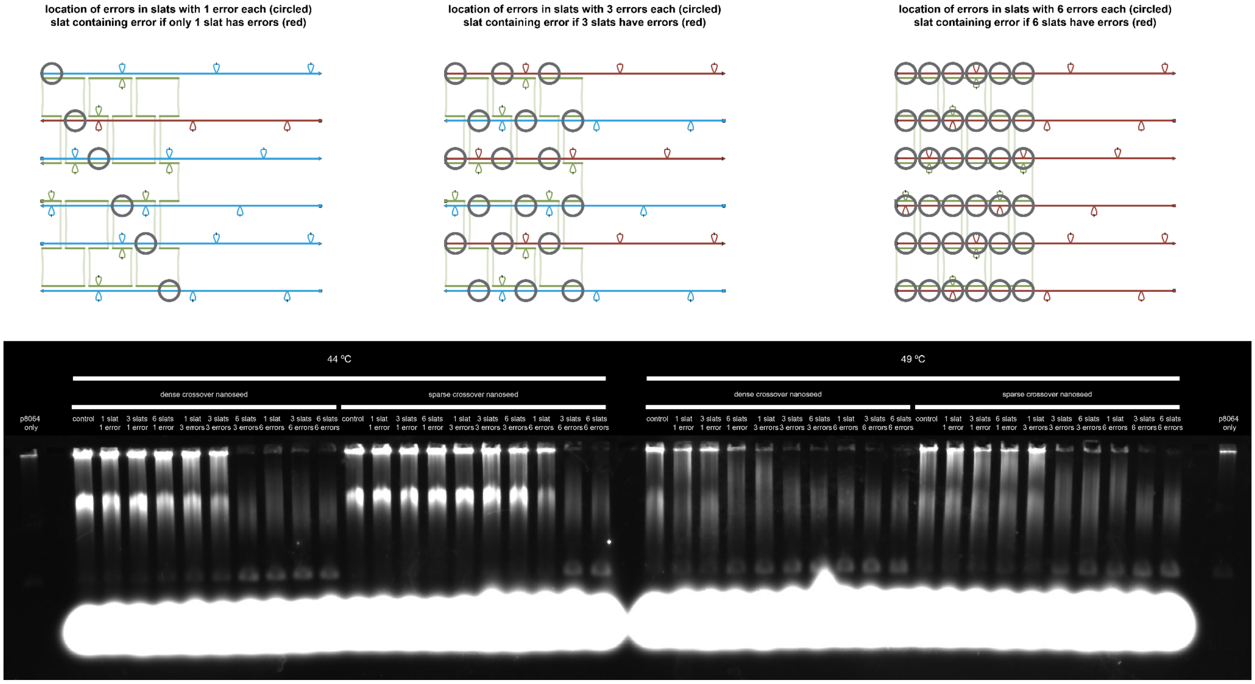

Supplementary Figure 36: Tolerance of nanoseeds to the presence of sequence mismatches, detected using v6.1 linear growth. Top: representation of mismatch positions in capture nuc-x slats for 1, 3, or 6 errors per slat and 1, 3, or 6 slats containing errors. Mismatches were chosen using NUPACK to make the 5/6 nt binding site as orthogonal as possible to correct binding with just a single nucleotide change. The same error pattern was used for both the sparse and dense crossover nanoseeds. Bottom: agarose gel using v6.1 linear growth[1] demonstrating higher tolerance to mismatches for the sparse crossover nanoseed, and higher tolerance at lower temperatures for both dense and sparse crossover nanoseeds. Reactions were performed at 12 mM Mg^{2+}, 3.5 mM Tris, 0.7 mM EDTA, pH 8.0, 0.01% Tween-20, 85 °C for 5 minutes then 44/49 °C overnight for ~17 hours using 0.5 µM/slat and 1 nM p8064 target.

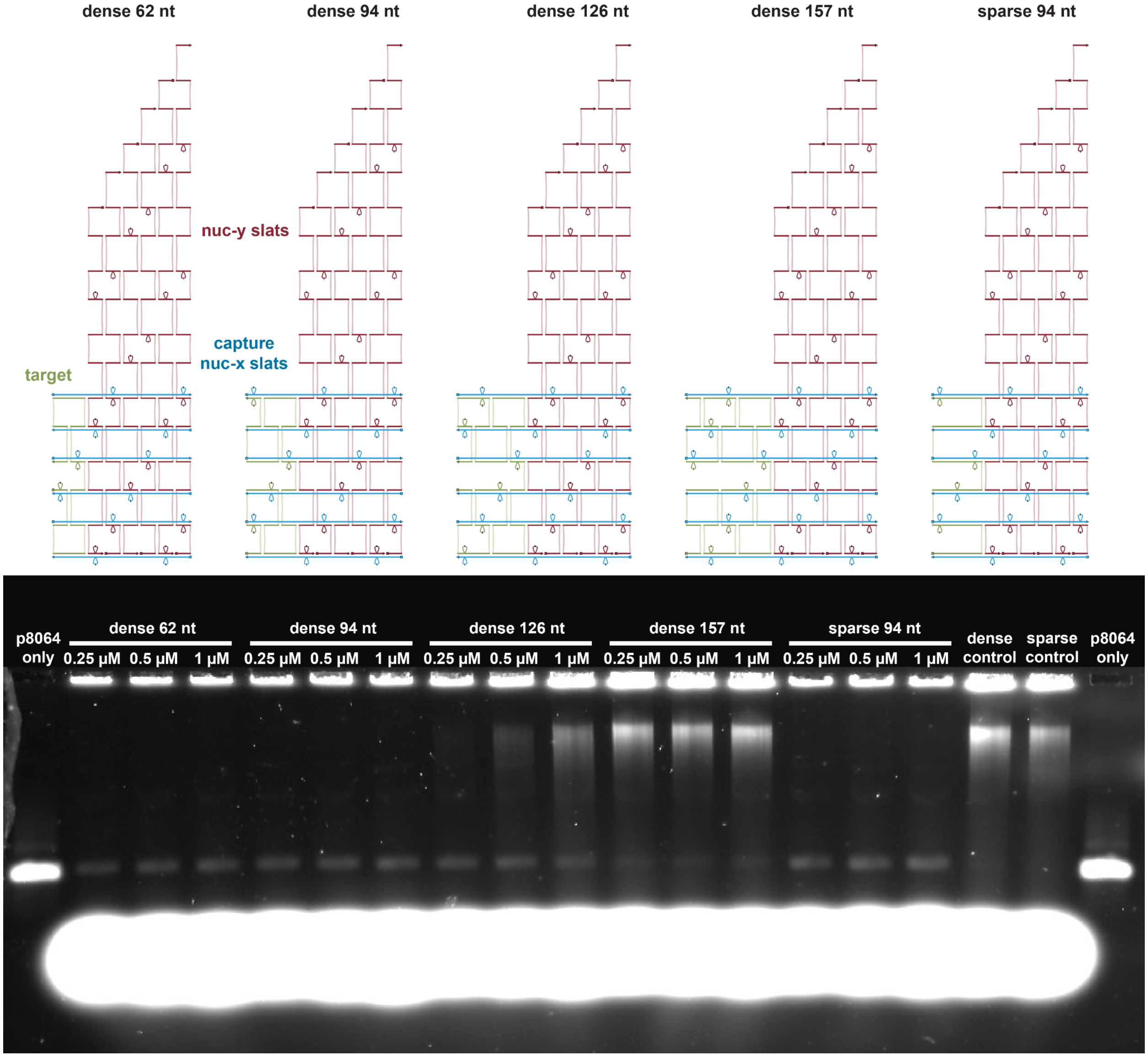

Supplementary Figure 37: Nanoseed formation of shorter target sequences by shortening capture nuc-x slats through the removal of the left-most binding sites in Supplementary Figure 29 and v6.1 linear growth (scadnano designs have growth-slats omitted for clarity). Reactions were performed at 14 mM Mg^{2+}, 3.5 mM Tris, 0.7 mM EDTA, pH 8.0, 0.01% Tween-20, 85 °C for 5 minutes then 46 °C overnight using 0.2 µM/growth- and nuc-y slat, variable nuc-x slat concentrations (as noted), and 1 nM p8064 target. Note: “p8064 only” lanes are stock 1 nM p8064 that was not incubated at reaction conditions.

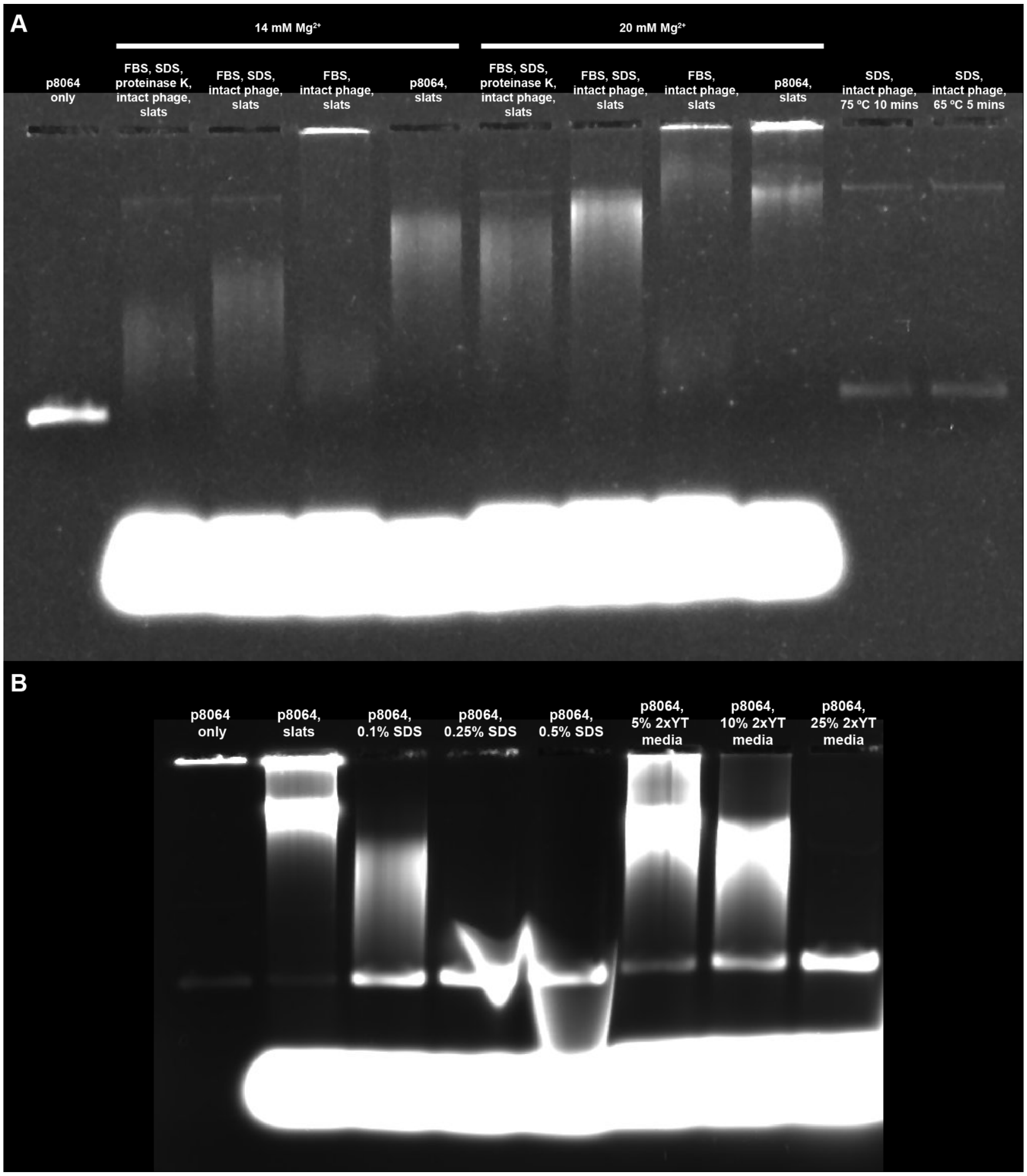

Supplementary Figure 38: A, Nanoseed detection with v6.1 linear growth of p8064 target from intact phage particles in the presence of 10% fetal bovine serum, and optionally 0.1% sodium dodecyl sulfate (SDS) and 1 mg/mL proteinase K. Reactions were performed at 14/20 mM Mg^{2+}, 3.5 mM Tris, 0.7 mM EDTA, pH 8.0, 0.01% Tween-20, 75 °C for 10 minutes then 50 °C overnight using 0.2 μM/slat and 0.1 nM p8064 target (as elsewhere in this work) or ~1 nM intact phage. Note: "p8064 only" lane is stock 1 nM p8064 that was not incubated at reaction conditions. **B**, Nanoseed detection with v6.1 linear growth in the presence of variable SDS or 2xYT microbial growth culture media. Reactions were performed at 14 mM Mg^{2+}, 3.5 mM Tris, 0.7 mM EDTA, pH 8.0, 0.01% Tween-20, 85 °C for 5 minutes then 50 °C overnight using 0.5 μM/slat and 1 nM p8064 target (in this case "p8064 only" was incubated at the reaction conditions as elsewhere in this work).

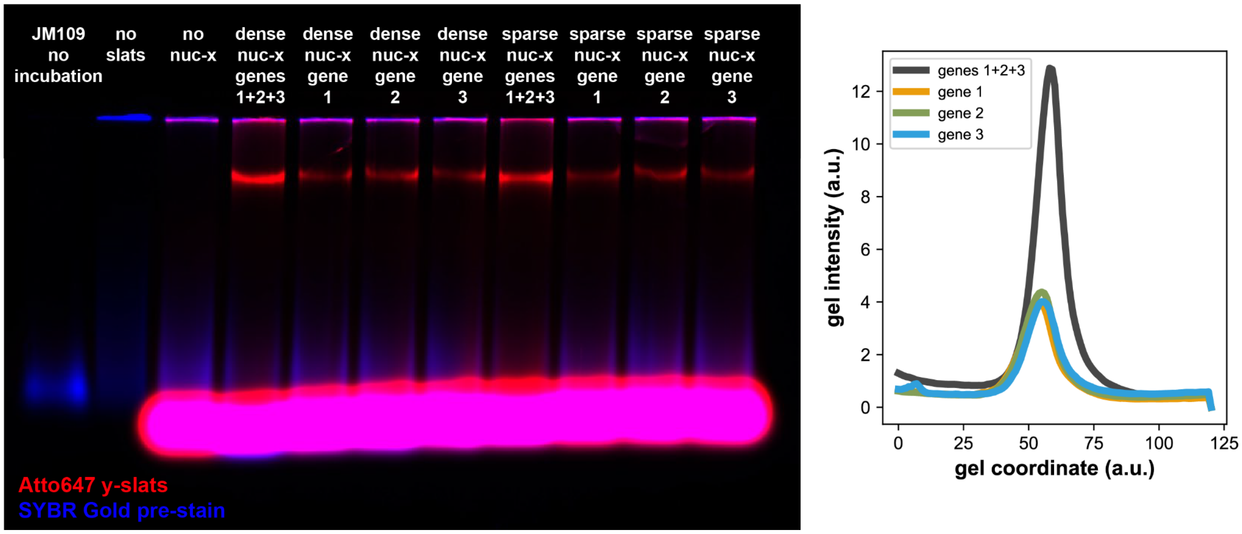

Supplementary Figure 39: Left: v6.1 linear crisscross detection of ~0.3 nM JM109 *E. coli* gDNA using both sparse and dense crossover nanoseed designs from Supplementary Figure 29. Reactions were performed at 12 mM Mg^{2+},3.5 mM Tris, 0.7 mM EDTA, pH 8.0, 0.01% Tween-20, 85 °C for 5 minutes then 46 °C overnight using 0.5 µM per growth and nuc-x slat, 0.25 µM/nuc-y slat and ~0.3 nM purified JM109 gDNA with capture nuc-x slats against both forward and reverse strands. Right: Gel densitometry of dense crossover Atto647 signal showing additive effects of detecting multiple genomic regions. The analysis was performed using the Python skimage.measure.profile_line function with linewidth set to approximately one third of the lane width centered at a homogeneous region of each gel band.

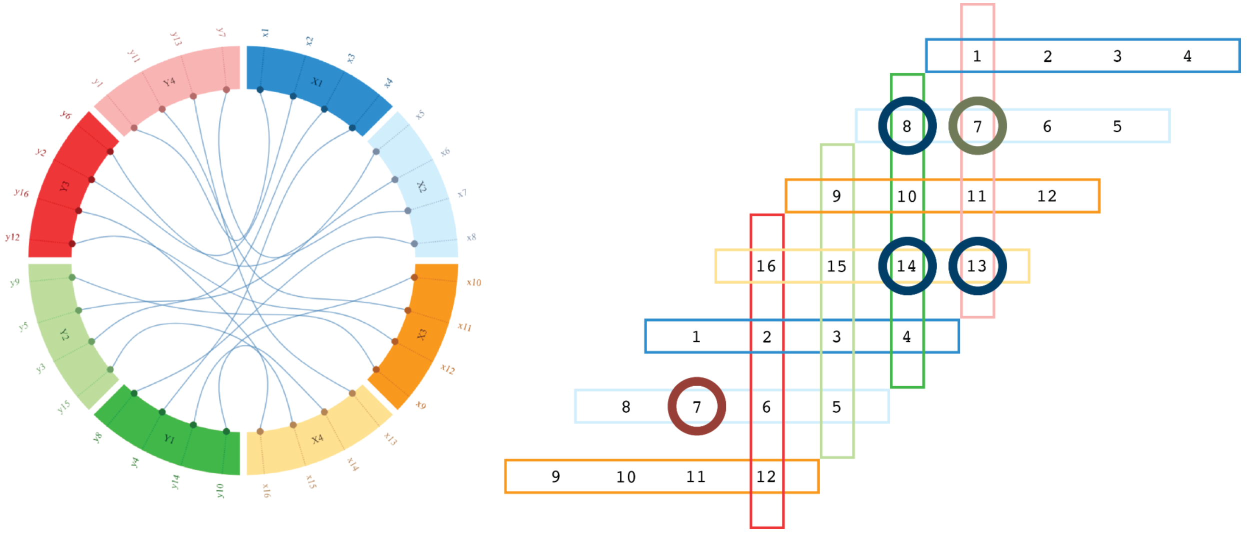

Supplementary Figure 40: v2 (core slat length 4) ribbon demonstrating 3-point constraint used to generate the context of rules in Kappa simulations. Left: contact map of 4 x-slats and 4 y-slats and the bonds between them (each unique bond numbered from 1 to 16). Right: example ribbon fragment based on the contact map. When considering whether bond 7 for slat Y4 (pink) can form intra-molecularly, we need to allow for the one circled in green to form, but not the one circled in red. The rule context checks whether Y4 is connected to the x-slat in question within 2 slats, e.g. by bonds 8, 14, and 13 (circled in blue).

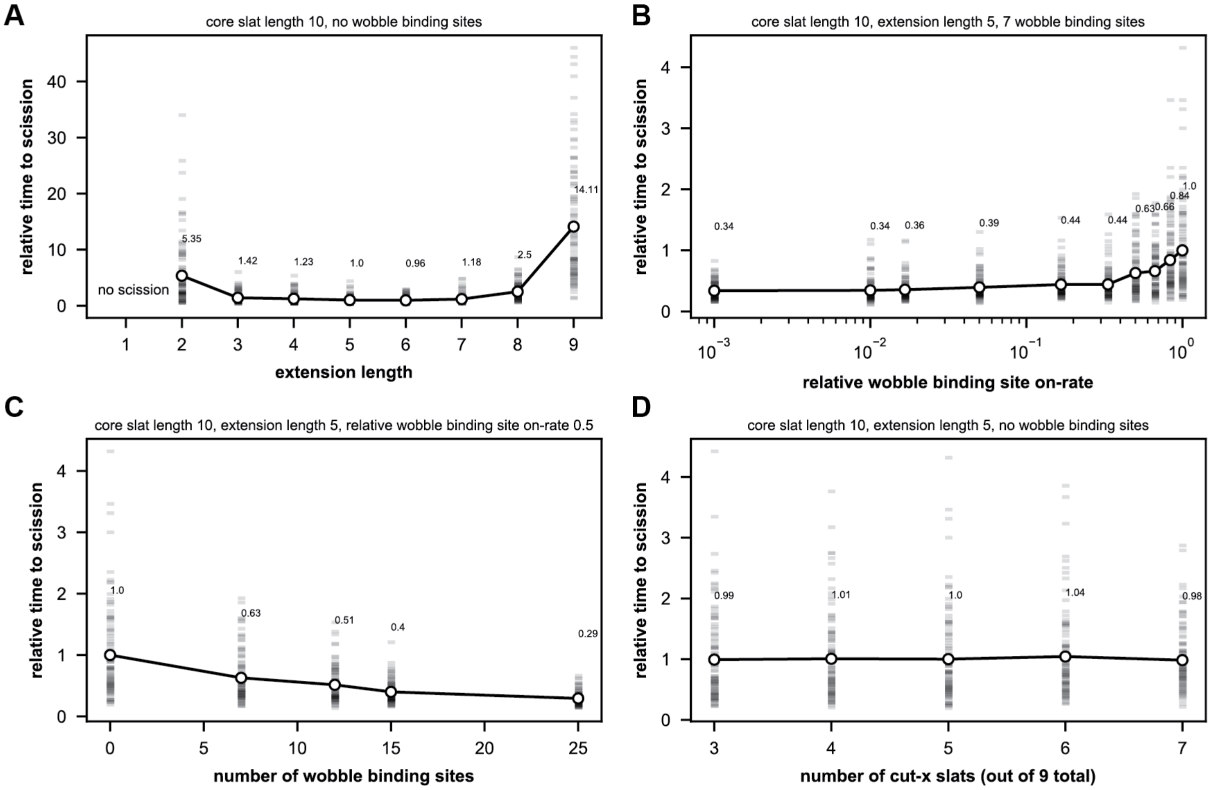

Supplementary Figure 41: Kappa simulations without pre-bound cut-slats and same default parameters and normalization as in Figure 4A and 4B. **A**, Effect of extension length (i.e. number of binding sites in the toehold) on ribbon scission rate. Increasing toehold length aided cut-slat recruitment and therefore increased scission rate; however longer toeholds allowed for the simultaneous recruitment of multiple cut-slats to the same set of extensions, thereby trapping the extensions from on-pathway interaction with any given cut-slat (see discussion in Method “Kappa simulation implementation” for discussion of how the choice of intra- and inter-complex on-rates affects the relative importance of extension length). **B** and **C**, Increasing the strength and number of wobble binding sites increased the rate of scission. **D**, Changing relative contributions of scission in the x- vs the y- direction does not significantly affect scission rate. Thus the number of cut-y slats is 9 minus the number of cut-x slats. All data-points are the mean of 100 simulations.

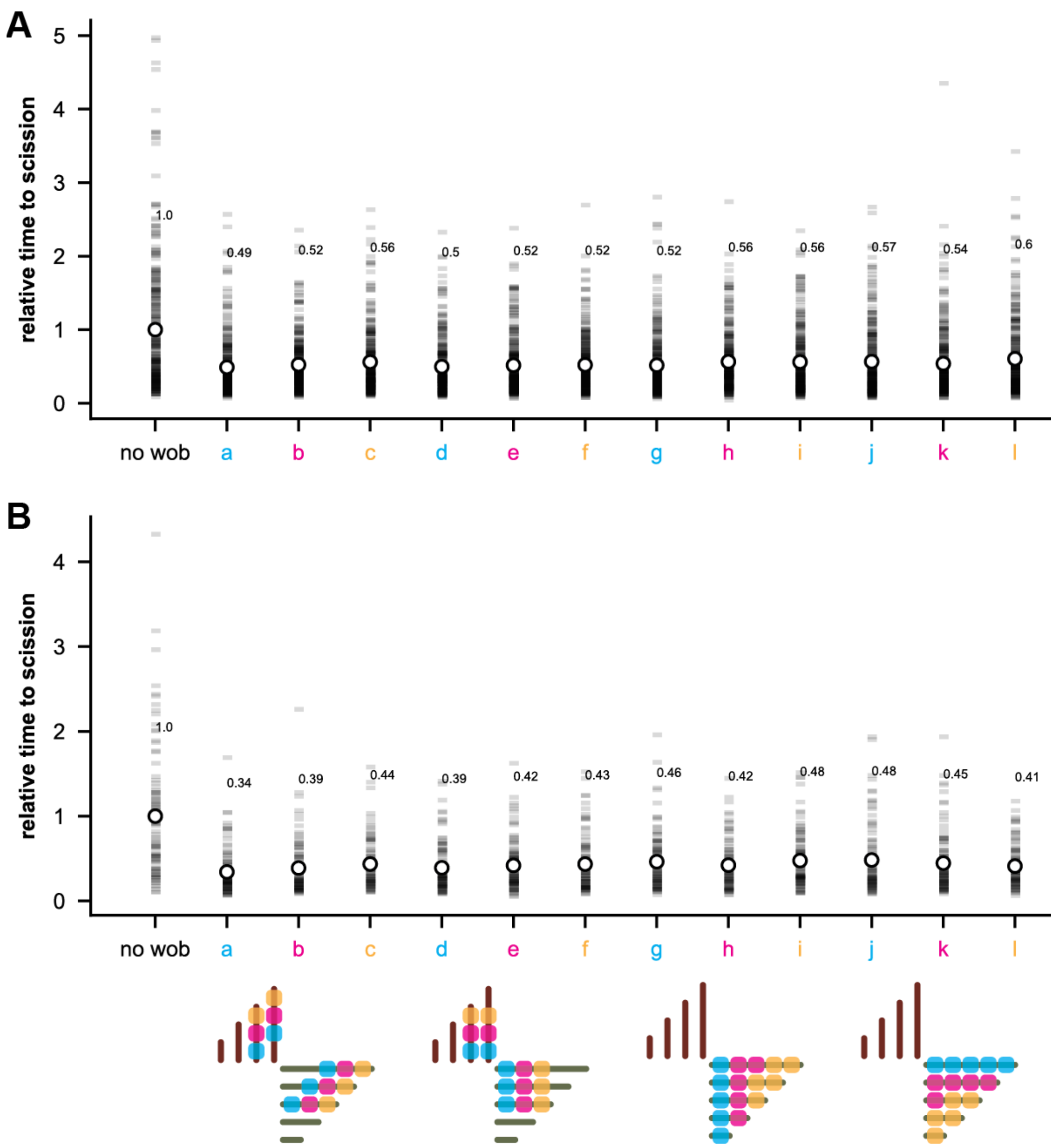

Supplementary Figure 42: Kappa simulations of different arrangements of 5 wobbles with cut-slats pre-bound. Colors of each letter on the x-axis corresponding to the positions in the diagrams directly below (a, b, c are the ones shown in Figure 4C). **A**, Using the same parameters as in Figure 4C (inter-complex on-rate 0.05, inter-complex off-rate 0.8, wobble on-rate scaling ⅔, wobble off-rate scaling 1.5, 300 simulations per condition). **B**, With different parameters displaying similar trends (inter-complex on-rate 0.04, inter-complex off-rate 1, wobble on-rate scaling ½, wobble off-rate scaling 2, 100 simulations per condition). While the effect size of different wobble arrangements on scission rate is not particularly significant, depending on the monomer design the effect on growth may be more profound, with wobbles closer to the outside potentially being better tolerated through introducing a smaller structural defect.

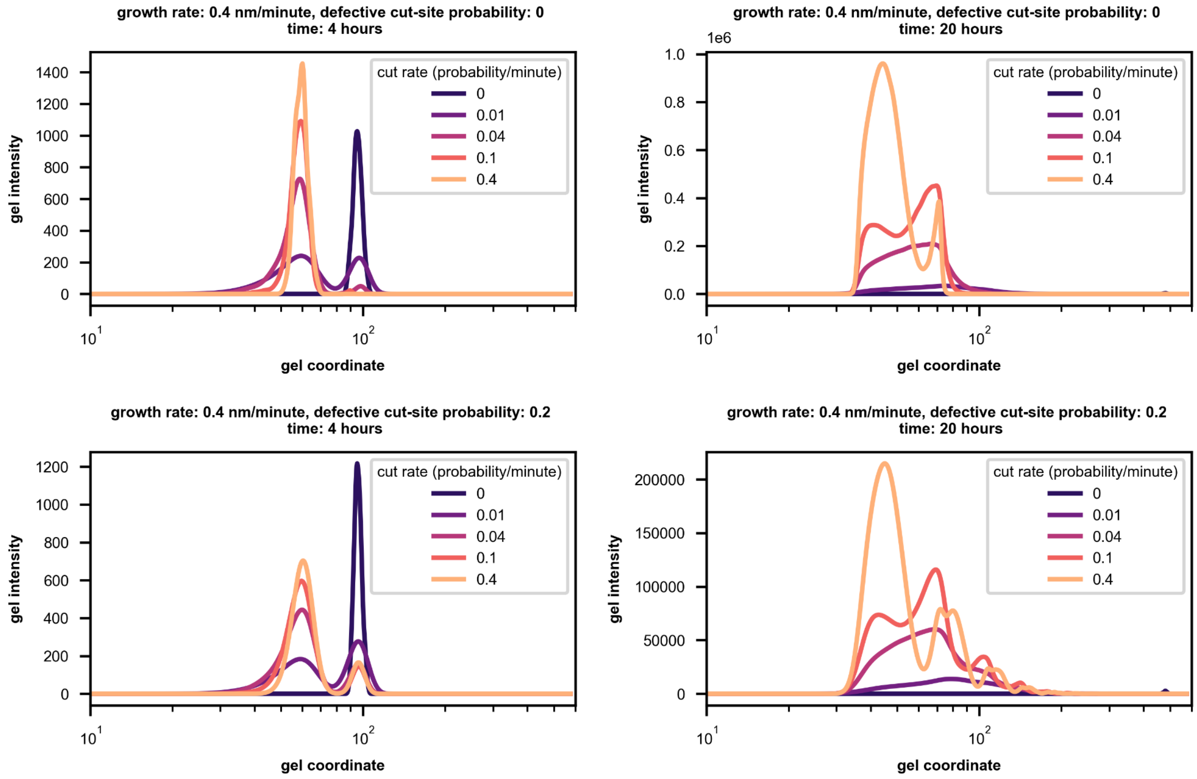

Supplementary Figure 43: Simple stochastic simulations purpose-built to predict gel-intensity profiles (i.e. not using Kappa) for ribbons growing at a rate of 0.4 nm/minute at different cutting rates. Simulations assume a repeat length of 36 nm (i.e. scission can only occur at 36 nm intervals). Simulations were initialized with 100 ribbons of length zero. At every 1-minute timestep, growth of each ribbon fragment occurs as a Poisson process with a mean of 0.4 nm, and cutting between each repeat unit occurs with a fixed probability equal to the specified cut rate. To model whether defects in toehold formation (e.g. if a given growth-slat is skipped during assembly), we assigned a fixed probability upon formation that a cut-site is incapable of being cut (e.g. 20%). To generate expected gel-intensities, a Gaussian kernel density estimate based on the distribution of ribbon lengths at the end of each simulation was multiplied by the number of ribbon fragments and the magnitude of each length evaluated to mimic the mass-dependent signal intensity of dye intercalation. Simulations were written in Python 3 and run locally on a 2019 iMac.

Earlier in the reaction process (e.g. 4 hours), even at higher cut-rates many ribbons are still full-length as the growth rate is rate limiting. After a longer reaction time (e.g. 20 hours), we primarily see peaks corresponding to ribbons of one and two repeats in length respectively, with the peaks being sharper for higher scission rates. Given that scission and growth is a continuous process, the presence of two distinct peaks and the variability in their intensities can be thought of as a single distribution split at the point of scission. Furthermore, we can see that if a fraction of the cut-sites are incapable of growth, we can reproduce some of the slower-migrating peaks seen experimentally.

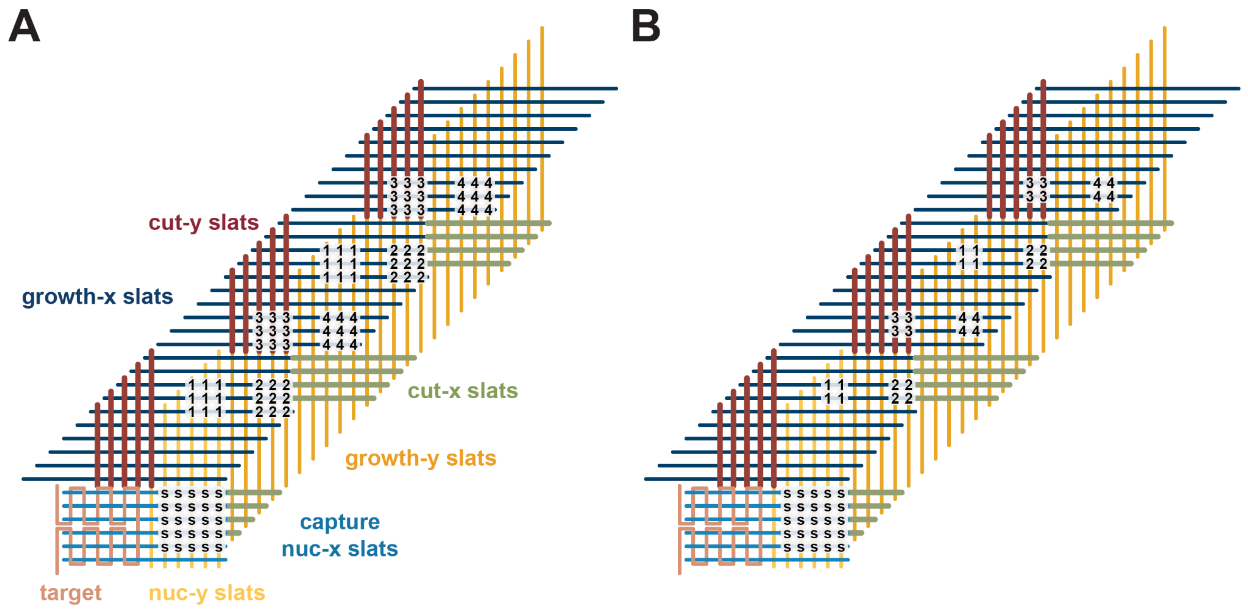

Supplementary Figure 44: Example of how algorithmic behavior (e.g. bit-copying) could be encoded in the current 3CR architecture (v5 growth with 5seg extensions and a repeat of 10 growth-x, 10 growth-y, 4 cut-x and 5 cut-y slats shown). **A**, 3×3 encoding block. Each variable segment, labeled with s or 1–4, can be occupied by one of two distinct sequences. Then different seeds, each encoding one of up to 2^9 or 512 varieties, recruit a corresponding distinct set of nuc-y slats, resulting in different binding-site sequences in the seed region "s" shown. Each of the 512 sets of nuc-x slats recruits a distinct set of nuc-y slats with corresponding sequences in binding sites at block "1". Growth-x slats with binding sites complementary to those in block "1" are recruited, resulting in translation to distinct corresponding sequences for binding sites in block "2". The same logical operation repeats for binding sites labeled with "3" and "4", and then back to "1". Thus the information is propagated cyclically via serial translation through the distinct representations in the four stages of development (i.e. blocks 1–4). In order to copy information in this way, there need to be 2^3 variants of 6 growth-y, 6 growth-x, and 3 cut-y slats, i.e. 105 additional slats needed to encode information provided by 512 possible seeds. In actual practice with this design, error accumulation would be very high. Therefore, proofreading would be needed for faithful propagation of information. If the 3×3 block is treated as a proofreading block (i.e. additional redundancy to copy information encoded by only two instead of 512 possible seeds, thereby exponentially reducing error rates[2]), then only 15 additional slats are needed, c.f. a repeat-unit of 29 slats for periodic growth with no algorithmic behavior. **B**, 2×2 encoding block. If every one of the labeled binding sites has two possible sequence values (i.e. binary encoding), there will be 2^2 variants of 4 growth-y, 4 growth-x, and 4 cut-y slats resulting in 2^4 possible encoded combinations, i.e. 30 additional slats needed to encode information provided by 16 possible seeds. If the 2×2 block is treated as a proofreading block, 10 additional slats are needed, c.f. a repeat-unit of 29 slats for periodic growth with no algorithmic behavior. Variations in which binding sites are used as logical bits and the relations between the encoding blocks could allow for encoding different algorithmic behaviors. Furthermore, changes in slat architecture could enable more compact encoding, for example as described by the Slat Assembly Model[3].

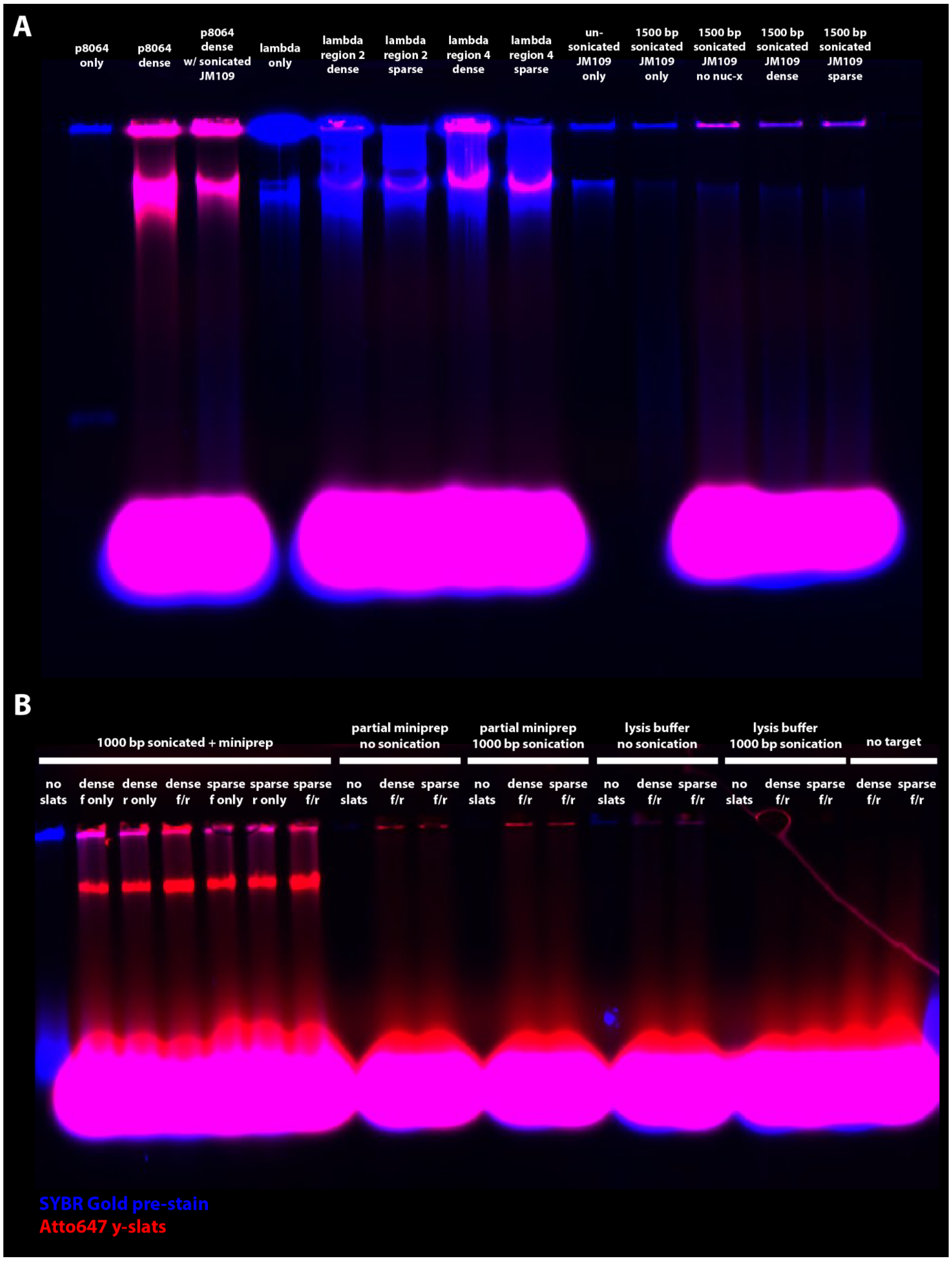

Supplementary Figure 45: Optimization of dsDNA detection with v6.1 linear growth. **A**, Detection of JM109 sample without miniprep is not possible, even with sonication. However, the presence of the JM109 sample does not appear to inhibit crisscross assembly, given that detection of p8064 is still possible. Reactions were performed at 12 mM Mg^{2+},3.5 mM Tris, 0.7 mM EDTA, pH 8.0, 0.01% Tween-20, 85 °C for 5 minutes then 46 °C overnight using 0.5 µM per slat, and ~1 nM target, with the JM109 sample (in 2xYT media occupying) ~31% of the reaction volume. **B**, The full miniprep with sonication seems to be necessary for JM109 detection. “Partial miniprep” skips the final spin step. Reactions were performed at 12 mM Mg^{2+},3.5 mM Tris, 0.7 mM EDTA, pH 8.0, 0.01% Tween-20, 85 °C for 5 minutes then 46 °C overnight using 0.5 µM per slat, and 5% of sample by volume.

Supplementary references

(1) Minev, D.; Wintersinger, C. M.; Ershova, A.; Shih, W. M. Robust Nucleation Control via Crisscross Polymerization of Highly Coordinated DNA Slats. *Nat. Commun.* **2021**, *12* (1), 1741. https://doi.org/10.1038/s41467-021-21755-7.

(2) Winfree, E.; Bekbolatov, R. Proofreading Tile Sets: Error Correction for Algorithmic Self-Assembly. In *DNA Computing*; Chen, J., Reif, J., Eds.; Lecture Notes in Computer Science; Springer: Berlin, Heidelberg, 2004; pp 126–144. https://doi.org/10.1007/978-3-540-24628-2_13.

(3) *Abstract Slat Assembly Model (aSAM) - self-assembly wiki*. http://self-assembly.net/wiki/index.php?title=Abstract_Slat_Assembly_Model_(aSAM) (accessed 2023-05-30).
